# Response Theory for Locally Gapped Systems

**DOI:** 10.1007/s10955-026-03703-1

**Published:** 2026-10-12

**Authors:** Joscha Henheik, Tom Wessel

**Affiliations:** 1 Mathematical Institute, University of Oxford, Radcliffe Observatory Quarter, Woodstock Road, OX2 6GG Oxford, United Kingdom; 2 Fachbereich Mathematik, Universität Tübingen, Auf der Morgenstelle 10, 72076 Tübingen, Germany

## Abstract

We introduce a notion of a *local gap* for interacting many-body quantum lattice systems and prove the validity of response theory and Kubo’s formula for localized perturbations in such settings. On a high level, our result shows that the usual spectral gap condition, which concerns the system as a whole, is not a necessary condition for understanding local properties of the system. More precisely, we say that an equilibrium state $\rho _0$ of a Hamiltonian $H_0$ is locally gapped in ${\varLambda ^{\textrm{gap}}}\subset \varLambda$, whenever the Liouvillian $- \textrm{i}\, [H_0, \, \cdot \, ]$ is almost invertible on local observables supported in ${\varLambda ^{\textrm{gap}}}$ when tested in $\rho _0$. To put this into context, we provide other alternative notions of a local gap and discuss their relations. The validity of response theory is based on the construction of *non-equilibrium almost stationary states* (NEASSs). By controlling locality properties of the NEASS construction, we show that response theory holds to any order, whenever the perturbation εV acts in a region which is further than $|\log \varepsilon |$ away from the non-gapped region $\varLambda \backslash \varLambda \textrm{gap}$.

## Introduction

Spectral gaps lie at the heart of many areas of physics and mathematics [4, 11, 18, 19, 39]. In particular, questions concerning the spectral gap lie at the center of several of the most challenging problems in physics, e.g. the Berry–Tabor [5] and Bohigas–Giannoni–Schmit [6] conjectures in quantum chaos, the Yang–Mills mass gap problem, or Haldane’s conjecture on integer valued antiferromagnetic Heisenberg chains [20, 21].

The great interest in spectral gaps, far beyond these famous conjectures, is rooted in the fact that their existence has tremendous effects on fundamental properties of the system. For example, in quantum many-body physics, the existence of a spectral gap above the ground state eigenvalue has far-reaching consequences for entanglement properties [22, 43] and ground state correlations [23]. On the other hand, the *closing* of a spectral gap is related to the occurrence of a (topological) quantum phase transition [1, 44]. Finally, the assumption of a spectral gap played a crucial role in recent proofs of adiabatic theory and linear response for many-body systems; see Sect. 1.1 for a detailed discussion.

However, an overall drawback in all of the above examples is that the desired spectral property is of *global* nature, i.e. involving the studied system as a whole, and thus seldom compatible with a notion of *locality* in an underlying physical space. Therefore, a natural question to ask is:

How can one express that a system is *locally* gapped, and which consequences that one has for globally gapped systems persist?

In this paper, we study this question in the setting of locally interacting many-body quantum spin lattice systems; see Sect. 2 for precise definitions. More precisely, in the above spirit of our guiding question, this paper has two main goals:

(i) We propose a notion of a *local gap* via a dynamical characterization and exemplarily prove that local perturbations of Hamiltonians with a frustration free product ground state satisfy this condition. Moreover, we study possible alternative notions of local gaps and their relations among each other; see Sects. 1.2 and 3.
(ii) As an application to a physically relevant problem, we show that for Hamiltonians with a local gap, response theory approximately holds to any order and thus justify *Kubo’s formula*; see Sects. 1.3 and 4.1.

Within the adiabatic many-body setting considered here, there are only a few works in the literature studying quantum systems under a non-standard gap condition, i.e. one differing from the clean separation of eigenvalues: In [14], the authors derive Kubo’s formula for two-dimensional disordered systems having only a *mobility gap*. Another recent paper dealing with spectral gaps in presence of disorder is [13]. Together with Teufel, one of us proved [26, Theorem 4.1] that finite systems, whose thermodynamic limit has a usual spectral gap, approximately obey an adiabatic theorem. In another recent work [52], the authors developed a theory of metastable states, characterized by the requirement that local operators raise the energy of such a state by a certain minimal amount. Their condition is similar to an alternative notion of a local gap given in (35) below. Moreover, several months after the first posting of this article the authors of [10] proposed a notion of a local spectral gap in the context of topological materials, modeled by one-body Hamiltonians. Finally, we remark that, in the context of Lie group theory, the notion of a “local gap” has recently been introduced [8] and proved itself to have profound consequences [7, 8].

Next, in Sect. 1.1, we discuss the problem of justifying linear (and higher order) response theory and Kubo’s formula based on adiabatic theory. Afterwards, in Sect. 1.2, we introduce our *local dynamical gap condition* $(\textrm{LDG}_{\textrm{informal}})$. Finally, in Sect. 1.3, we discuss our main result on response theory.

### Response Theory in Many-Body Quantum Systems

The purpose of response theory is to express how quantum expectation values change, after a small perturbation is slowly turned on. More precisely, one considers an unperturbed Hamiltonian $H_0$ with equilibrium state (usually a ground state) $\rho _{0}$ and slowly (adiabatically) turns on a small additive perturbation εV. Denoting by $\rho _\varepsilon$ the state of the system after εV has been turned on, one aims to understand, how the expectation value of an observable B changes, i.e. determine

1 $$\begin{aligned} \langle B \rangle _{\rho _\varepsilon } - \langle B \rangle _{\rho _0} = \varepsilon \, \sigma _B + o(\varepsilon ) \end{aligned}$$

at least to leading order in the strength ε.

A central piece of response theory is *Kubo’s formula* [36], which provides a simple expression for the so-called linear response coefficient $\sigma _B$ in (1). Despite the simplicity and empirical success of Kubo’s formula, the problem of justifying it in a very general framework has so far escaped rigorous treatment. The fundamental difficulty lies in the fact that the state $\rho _\varepsilon$ is no longer an equilibrium state in general, and therefore determining it is outside the powerful realm of equilibrium statistical mechanics. This problem has prominently been pointed out by Simon [48] in 1984 in his “Fifteen problems in mathematical physics”, containing a rigorous justification of Kubo’s formula from first principles as problem (4B).

However, in the particular setting of many-body lattice systems with a spectral gap at zero temperature, it has recently been possible [3, 25, 40, 49] to actually prove Kubo’s formula and justify the applicability of linear response theory to compute the change in expectation values (1). The more general underlying results establish *generalized super-adiabatic theorems*^1^ for short-range interacting Hamiltonians, which can be written as a sum of local terms, and are hence called *SLT operators* [26, 27, 29].

The recent breakthrough, which paved the way for these results, was achieved by Bachmann et al. [3], who proved the first adiabatic theorem for extended (but finite) quantum lattice systems; see [40] for an adaptation to fermionic systems. One key difficulty is that, for macroscopic systems, typical operator norm bounds in adiabatic theory deteriorate due to the *orthogonality catastrophe* and one instead has to formulate the result in a weaker topology by testing against local observables. On a high level, the main ingredient for their proof are the well-known *Lieb-Robinson bounds* (LRBs) [37], which ensure a finite speed of propagation and prevent build-up of long-range entanglement. These LRBs enabled to prove that the generator of the *spectral flow*, introduced by Hastings and Wen [24], is in fact an SLT operator and hence maintains good locality properties [1, 24] (showing so-called *automorphic equivalence* of ground states).

However, the work [3] had the limitation that the spectral gap of $H_0$ is assumed to remain open after adding the perturbation εV. To allow εV to close the gap, Teufel [49] combined ideas from space-time adiabatic perturbation theory [46, 47] with locality estimates from [3]. The underlying perturbative scheme is an iterative application of locality preserving Schrieffer–Wolff transformations (a.k.a. Lie–Schwinger block diagonalization [17]), which proved to be a powerful approach in several rather recent works in that direction [3, 13, 26, 27, 49, 51, 52]. In this paper, we carefully exploit locality properties of the operations involved in the perturbative scheme, which allows dealing with *locally* gapped systems; see Sect. 1.2 below.

**Fig. 1:**
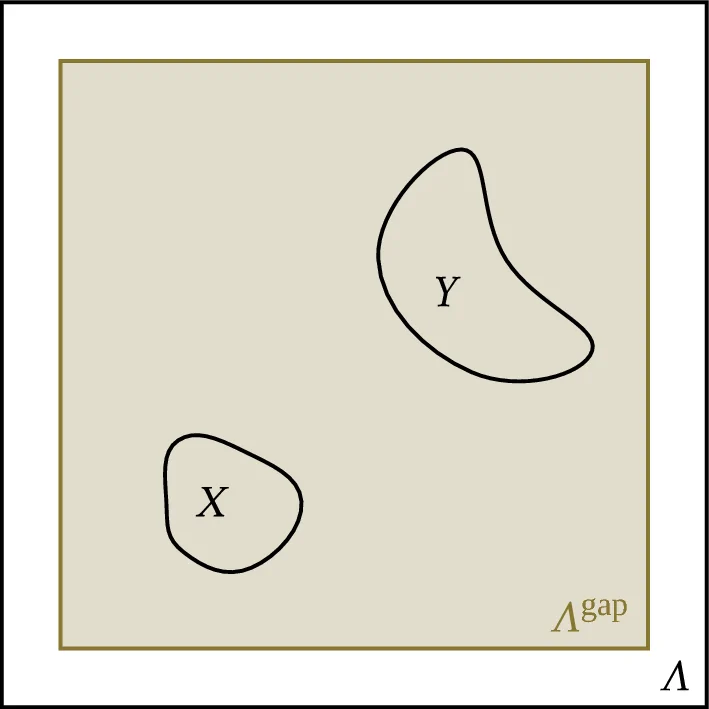
Illustrated is the local dynamical gap condition $(\textrm{LDG}_{\textrm{informal}})$ in the case where the system is gapped in the bulk of Λ, e.g. due to gap closing edge modes. If the observables $A \in \mathcal {A}_X$ and $B \in \mathcal {A}_Y$ are supported well inside ${\varLambda ^{\textrm{gap}}}$, the RHS of (4) is small, i.e. the Liouvillian is locally almost invertible

The rough physical picture underlying the works [46, 47, 49] is that a gap that is locally intact after adding the perturbation should be sufficient for adiabatic theory to be valid (cf. [49, Figure 1]). The results from [3, 40, 49] for large but finite systems were subsequently extended to the thermodynamic limit [26, 27], building on an extension of the spectral flow techniques to infinite systems by Moon and Ogata [41]. We point out that, contrary to [27], the papers [26, 41] assumed a spectral gap *only* for the GNS Hamiltonian of the infinite system (a *gap in the bulk*). More comprehensive reviews of the developments discussed in this section are given in [25, 29].

Let us stress that mathematical linear response theory extends well beyond the adiabatic-type theory of gapped many-body quantum lattice systems reviewed above. Early works already studied linear response in the framework of KMS states [45], and Kubo formulas and similar relations have been established in algebraic quantum statistical mechanics and for open quantum systems in [30–32]. Another line of results concerns random Schrödinger operators and the Anderson model: Kubo-type formulae for the ac-conductivity were derived without an a priori spectral gap assumption [33] (see [16, 34, 35] for further developments). We also mention the algebraic perturbative approach of [9], where response expansions are constructed using Lieb-Robinson bounds for multi-commutators, as well as the analytic-algebraic framework of [12]. These results are of a different nature from the adiabatic lattice-system approach discussed here, and we do not attempt to review this broad literature. Rather, our focus is on weakening the gap assumptions in the adiabatic response theory of many-body quantum lattice systems.

In fact, in view of the linear response problem and the second of our principal goals, the contribution of this paper is to extend the previous results for uniformly or bulk gapped systems to systems where $H_0$ is locally gapped. This important extension allows to rigorously treat systems with impurities or gap-closing edge modes; see the discussions in Sects. 1.2–1.3 below. Technically, our contribution is to control operations on SLT operators, which are localized on a subregion of the whole lattice; see Sect. 2.2 and Appendix A.

Lastly, we remark that we only consider finite-dimensional spaces and bounded operators for simplicity of the presentation.

### A Local Dynamical Gap Condition

All the previous results on linear response and adiabatic theory mentioned in Sect. 1.1 above, heavily rely on the range of the initial state $\rho \equiv \rho _0$ being contained in a gapped part of the spectrum of the unperturbed Hamiltonian $H \equiv H_0$.^2^ More precisely, these results assume that the spectrum of H can be decomposed as

2 $$\begin{aligned} {{\,\textrm{spec}\,}}(H) = \sigma _1 \cup \sigma _2 \qquad \text {with} \qquad d( \sigma _1, \sigma _2 ) \ge g \end{aligned}$$

for some gap size g>0 such that $\sigma _1 \subset I \subset \mathbb {R} {\setminus } \sigma _2$ for some compact interval I. Then, they require $P \, \rho \, P = \rho$, where P denotes the spectral projection^3^ associated to H onto $\sigma _1$.

On a technical level, in all of the works [3, 26, 27, 40, 49], the crucial importance of the gap of H lies in the local invertibility of the Liouvillian. That is, there exists an explicit, locality preserving map $\mathcal {I}_{H, g} = \mathcal {I}_{H, g}\llbracket \,\cdot \,\rrbracket$, which is often called *quasi-local inverse of the Liouvillian* on the observable algebra, depending on the SLT Hamiltonian H and the gap size g, which inverts the Liouvillian $\mathcal {L}_{H}\llbracket \,\cdot \,\rrbracket := -\textrm{i}\, [H, \,\cdot \,]$ in the projection $\langle \,\cdot \,\rangle _{P} = {{\,\textrm{tr}\,}}\bigl ( P \,\cdot \,\bigr )$ onto $\sigma _1$. More precisely, for all local observables A,B it holds that

3 $$\begin{aligned} \bigl \langle [\mathcal {L}_{H} \circ \mathcal {I}_{H, g} \llbracket A \rrbracket - A , B ] \bigr \rangle _{P} = 0 . \end{aligned}$$

Surprisingly, the Hamiltonian H having a spectral gap is actually *equivalent* to the invertibility of the Liouvillian in the above sense, see Proposition 3.1.

The goal of this article is to relax the requirement of a globally spectrally gapped Hamiltonian H and instead work with a so-called *local dynamical gap condition*. This condition roughly asserts that, the Hamiltonian H behaves as if it had a gap in a spatially localized region, i.e. that the Liouvillian can (almost) be locally inverted in that region. A more formal version of $(\textrm{LDG}_{\textrm{informal}})$ is formulated in Assumption $(\textrm{LDG}_{\textrm{main}})$ in Sect. 3.1.

**(LDG**_**informal**_**) Local dynamical gap condition** (informal version) *Let* H *be an SLT Hamiltonian and* ρ *an equilibrium state of* H, *i.e.* [H,ρ]=0. *We say that* ρ *is* locally dynamically gapped *of size at least* g>0 *in a region* ${\varLambda ^{\textrm{gap}}}\subset \varLambda$ *if and only if for all observables*^4^$A \in \mathcal {A}_X$ *and* $B \in \mathcal {A}_Y$ *localized in* $X\subset \varLambda$ *and* $Y \subset \varLambda$, *it holds that*4*for some fixed* $\ell \in \mathbb {N} _0$ *and constants* C, q>0, *independent of the sizes* |Λ| *and* $|{\varLambda ^{\textrm{gap}}}|$. ⋄

In a nutshell, this means that, within ${\varLambda ^{\textrm{gap}}}$, the Hamiltonian H approximately behaves as if it were spectrally gapped—up to an error vanishing (stretched) exponentially fast in the distance to $\varLambda {\setminus } {\varLambda ^{\textrm{gap}}}$. On the physical level, one might think of $\varLambda {\setminus } {\varLambda ^{\textrm{gap}}}$ as some impurity region causing the global spectral gap to close, or the boundary of Λ and hence allowing for gap-closing edge modes; see Fig. 1. Indeed, as we will show in Sect. 3.5, the local gap condition $(\textrm{LDG}_{\textrm{informal}})$ is satisfied for ground states of locally in $\varLambda {\setminus } {\varLambda ^{\textrm{gap}}}$ (but arbitrarily strongly) perturbed Hamiltonians of certain quantum spin systems, which have a globally gapped ground state.

#### Verifying the Local Dynamical Gap Condition

Despite the supportive examples above, our concrete formulation of a local gap condition $(\textrm{LDG}_{\textrm{informal}})$ might still seem a bit *ad hoc* at the moment. Therefore, we will outline several alternative ways to formulate such a condition and discuss their respective features and relations in Sect. 3. In particular, in Proposition 3.2 we show the following (the constants q,C,ℓ have the same meaning as in (4) but might take different values):

(i) Let the SLT Hamiltonian H with ground state ρ be obtained from a globally gapped SLT Hamiltonian $H_*$ with ground state $\rho _*$ by an SLT perturbation J localized in $\varLambda {\setminus } {\varLambda ^{\textrm{gap}}}$, i.e. $H = H_* + J$. If the difference $\rho - \rho _*$ is locally small in the sense that
  $$\begin{aligned} \Vert (\rho - \rho _*) \, A \Vert _{{{\,\textrm{tr}\,}}} + \Vert A \, (\rho - \rho _*) \Vert _{{{\,\textrm{tr}\,}}} \le C \, \Vert A \Vert \, \bigl ( 1+{{\,\textrm{diam}\,}}(X) \bigr )^\ell \exp \bigl ( -d( X, \varLambda \setminus {\varLambda ^{\textrm{gap}}})^{q} \bigr ) \end{aligned}$$
  for all $X\subset \varLambda$ and $A\in \mathcal {A}_X$, then ρ is locally dynamically gapped.
(ii) In the same setting as in (i) the following holds: If there exists a norm-preserving automorphism τ on the observable algebra such that $\langle \,\cdot \,\rangle _\rho = \langle \tau \llbracket \,\cdot \,\rrbracket \rangle _{\rho _*}$ and
  $$\begin{aligned} \bigl \Vert ( \tau - \textbf{1})\llbracket A \rrbracket \bigr \Vert \le C \, \Vert A \Vert \, \bigl ( 1+{{\,\textrm{diam}\,}}(X) \bigr )^\ell \, \exp \bigl ( -d( X, \varLambda \setminus {\varLambda ^{\textrm{gap}}})^{q} \bigr ) \end{aligned}$$
  for all $X\subset \varLambda$ and $A\in \mathcal {A}_X$, then ρ is locally dynamically gapped.
(iii) Let H be an SLT Hamiltonian and $\rho = |\psi \rangle \langle \psi |$ its pure *product ground state*. Then, if ρ is effectively gapped well inside ${\varLambda ^{\textrm{gap}}}$ in the sense that^5^
  $$\begin{aligned} \textrm{i}\, \bigl \langle A^* \mathcal {L}\llbracket A \rrbracket \bigr \rangle _\rho \!\ge \! g \, \Bigl ( 1\!-\! C \, \bigl ( 1\!+\!{{\,\textrm{diam}\,}}(X) \bigr )^\ell \, \exp \bigl ( \!-\!d( X, \varLambda \setminus {\varLambda ^{\textrm{gap}}})^{q} \bigr ) \Bigr ) \, \Bigl ( \langle A^* A \rangle _\rho \! -\! \bigl |\langle A \rangle _\rho \bigr |^2 \Bigr ) \end{aligned}$$
  for all $X\subset \varLambda$ and $A \in \mathcal {A}_X$, then ρ is locally dynamically gapped.

Items (i) and (ii) will be used in Sect. 3.5.2, to show that ground states of perturbations of gapped frustration free Hamiltonians have a local dynamical gap.

### Discussion of Our Main Result

**Fig. 2:**
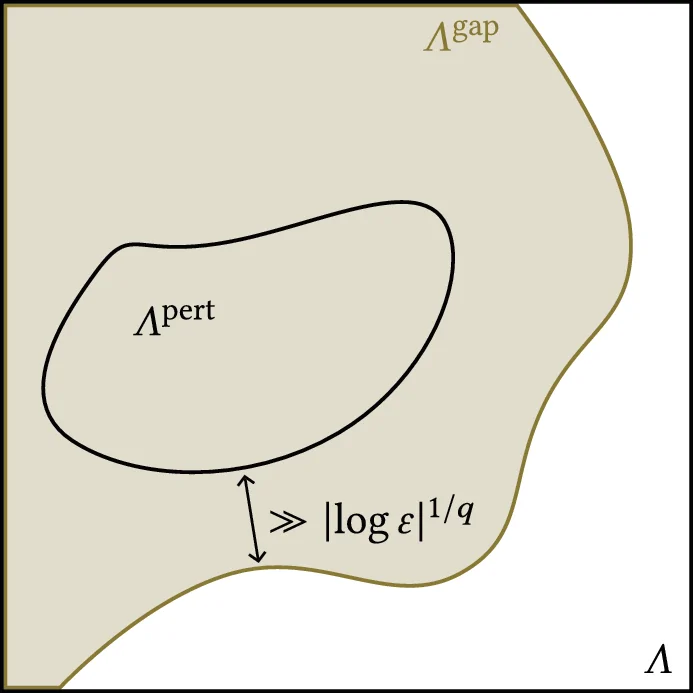
Let $H_0$ be locally dynamically gapped in ${\varLambda ^{\textrm{gap}}}$ and consider a small $\varLambda ^{\textrm{pert}}$-localized perturbation εV, which is adiabatically turned on. Then, if the distance between $\varLambda ^{\textrm{pert}}$ and $\varLambda {\setminus } {\varLambda ^{\textrm{gap}}}$ is large compared to $|\log \varepsilon |^{1/q}$, response theory (8) holds to any order

We can now formulate an informal version of our main result as a showcase application of our local gap condition $(\textrm{LDG}_{\textrm{informal}})$ to a physically relevant problem—the validity of response theory. In a nutshell, it says that, even after relaxing the usual condition of a global gap to $(\textrm{LDG}_{\textrm{informal}})$, we have *response theory to all orders* for a perturbation localized in ${\varLambda ^{\textrm{pert}}}$—provided that $d( {\varLambda ^{\textrm{pert}}}, \varLambda {\setminus } {\varLambda ^{\textrm{gap}}})$ is sufficiently large compared to $|\log (\varepsilon ) |^{1/q}$, where ε>0 is the strength of the perturbation and q∈(0,1) is some small constant; see (10) and Fig. 2.

More precisely, let $H_0$ be an SLT Hamiltonian and $\rho _0$ an equilibrium state of $H_0$ that is locally dynamically gapped in ${\varLambda ^{\textrm{gap}}}$ according to Assumption $(\textrm{LDG}_{\textrm{informal}})$. Let V be a ${\varLambda ^{\textrm{pert}}}$-localized perturbation by an SLT Hamiltonian as defined in (18), $f: \mathbb {R} \rightarrow [ 0,1 ]$ a smooth switching function with f(t)=0 for t≤-1 and f(t)=1 for t≥0, and define

5 $$\begin{aligned} H_\varepsilon (t) := H_0 + \varepsilon f(t) V . \end{aligned}$$

Moreover, let $\rho ^{\varepsilon , \eta , f}(t)$ be the solution of the time-dependent adiabatic Schrödinger equation

6 $$\begin{aligned} \textrm{i}\, \eta \, \frac{\textrm{d}}{\textrm{d}t} \rho ^{\varepsilon , \eta , f}(t) = \bigl [ H_\varepsilon (t) , \rho ^{\varepsilon , \eta , f}(t) \bigr ] \end{aligned}$$

with adiabatic parameter $\eta \in (0,1]$ and initial datum $\rho ^{\varepsilon , \eta , f}(t) = \rho _0$ for all t≤-1. Finally, for an observable $B \in \mathcal {A}_{Y}$ define the response to the perturbation as

7 $$\begin{aligned} \varSigma _B^{\varepsilon , \eta , f}(t) := \langle B \rangle _{\rho ^{\varepsilon , \eta , f}(t)} - \langle B \rangle _{\rho _0} . \end{aligned}$$

#### Main Result

(see Theorem 4.1) For every $j \in \mathbb {N}$ there exists a response coefficient $\sigma _{B,j}$, independent of $\varepsilon , \eta$ and f, such that the following holds: There exists a constant q∈(0,1) and for every $n,m \in \mathbb {N}$ and T>0, there exists a constant C>0, independent of ε, such that for every t∈[0,T] we have that

8 $$\begin{aligned} \begin{aligned} \sup _{\eta \in \bigl [ \varepsilon ^m , \varepsilon ^{\frac{1}{m}} \bigr ]} \biggl |\varSigma _B^{\varepsilon , \eta , f}(t) - \sum _{j=1}^{n} \varepsilon ^j \sigma _{B,j} \biggr |\le C \, {\left| \left| \left| B \right| \right| \right| } \, \varepsilon ^{n+1} \, \Bigl ( 1+ \textrm{e}^{-d( {\varLambda ^{\textrm{pert}}}, \varLambda \setminus {\varLambda ^{\textrm{gap}}})^{q} - C \, \log (\varepsilon )} \Bigr ) , \end{aligned} \end{aligned}$$

where ${\left| \left| \left| B \right| \right| \right| }$ measures the norm of B and its support Y.

The first order coefficient is given by *Kubo’s formula*

9 $$\begin{aligned} \sigma _{B,1} = - \textrm{i}\, \bigl \langle \bigl [ \mathcal {I}_{H_0,g}\llbracket V \rrbracket , B \bigr ] \bigr \rangle _{\rho _0} . \end{aligned}$$

When ${\varLambda ^{\textrm{gap}}}= \varLambda$, the equilibrium state $\rho _0$ of $H_0$ is *globally* dynamically gapped and the exponential in (8) is absent since $d( {\varLambda ^{\textrm{pert}}}, \varnothing ):= \infty$. This special case of our result (when $\rho _0 = P_0/\dim {{\,\textrm{rank}\,}}P_0$ and $P_0$ projects onto a gapped spectral patch) has already been proven in [49] with extensions to infinite systems in [26, 27]; see also Remark 4.2 below. Moreover, observe that, whenever

10 $$\begin{aligned} d( {\varLambda ^{\textrm{pert}}}, \varLambda \setminus {\varLambda ^{\textrm{gap}}}) \gg |\log (\varepsilon ) |^{1/q}, \end{aligned}$$

with q from our main result, the exponential in (8) is small compared to 1. In particular, this is the case, if $d( {\varLambda ^{\textrm{pert}}}, \varLambda {\setminus } {\varLambda ^{\textrm{gap}}}) \gg \varepsilon ^{-\delta }$ for some (arbitrarily small) δ>0. Hence, our main result gives an effective condition on the distance from the perturbation region, ${\varLambda ^{\textrm{pert}}}$, to the non-gapped region, $\varLambda {\setminus } {\varLambda ^{\textrm{gap}}}$, in comparison to the perturbation strength ε, which ensures that response theory to all orders is valid; see Fig. 2.

It is interesting to compare our main result to the adiabatic theorem [26, Theorem 4.1] for finite systems with a gap in the bulk, which are *locally gapped* in some sense. By an argument as in [49, Proof of Theorem 4.1], [26, Theorem 4.1] yields the analog of (8) again with an *additional error term*: Instead of the exponential in (8), one obtains^6^$\mathcal {O}\bigl ( \bigl ( \varepsilon ^C d( Y, \varLambda {\setminus } {\varLambda ^{\textrm{gap}}}) \bigr )^{-\infty } \bigr )$. Therefore, in order to have response theory to all orders in this setting, one needs that

11 $$\begin{aligned} d( Y, \varLambda \setminus {\varLambda ^{\textrm{gap}}}) \gg \varepsilon ^{-C} . \end{aligned}$$

We point out the following two differences between the conditions in (10) and (11): First, in (10), the relevant distance is between ‘where the perturbation V acts’, i.e. ${\varLambda ^{\textrm{pert}}}$, and ‘where we do not have a gap’, i.e. $\varLambda {\setminus } {\varLambda ^{\textrm{gap}}}$. In contrast to that, the relevant distance in (11) is between ‘where the observable B tests’, i.e. Y, and ‘where we do not have a gap’, i.e. $\varLambda {\setminus } {\varLambda ^{\textrm{gap}}}$. Second, while in (10) the distance must be much bigger than a power of the *logarithm* of the perturbation strength ε, in (11), the distance must be much bigger than a large *inverse power* of ε.

### Structure of the Paper

The rest of this paper is structured as follows. We begin by introducing the mathematical framework, in particular the underlying space and the concept of locality of SLT operators, in Sect. 2. Afterwards, in Sect. 3 we discuss the problem of formulating a local gap condition, formulate different variants and explain their connections, and, moreover, show certain exemplary systems to have a local dynamical gap. In Sect. 4, we precisely formulate our main result, Theorem 4.1, and also give its proof based on the NEASS construction. Proofs concerning the formulation of the local gap condition are given in Sect. 5. Afterwards, in Sect. 6, we perform the NEASS construction and prove the necessary inputs for Theorem 4.1; several technical lemmata and auxiliary results are deferred to Appendix A.

## Mathematical Framework

In this section, we briefly introduce the (standard) mathematical framework used in the formulation of the adiabatic theorems. For similar setups see [3, 26, 27, 49].

### Spatial Structure and Algebra of Observables

We consider a quantum spin system on a finite graph Λ equipped with the graph distance $d(\,\cdot \,, \,\cdot \,)$. Let $B_r(x):= \{ \, y \in \varLambda \,\vert \, d(x,y) \le r \, \}$ be the ball of radius r centered at $x \in \varLambda$. The graph is assumed to have dimension (at most) d>0, i.e. there exists a constant $C_{\textrm{vol}}> 0$ such that for all r≥0

12 $$\begin{aligned} \sup _{x \in \varLambda } |B_r(x) |\le 1 + C_{\textrm{vol}}\, r^d , \end{aligned}$$

where |X| denotes the number of sites in $X \subset \varLambda$. The set of all such graphs Λ is denoted by

13 $$\begin{aligned} \mathcal {G}(d, C_{\textrm{vol}}) := \Bigl \{ \, \varLambda \text { finite graph} \,\Big \vert \, \sup _{x \in \varLambda } \, |B_r(x) |\le 1 + C_{\textrm{vol}}\, r^d \text { for all } r > 0 \, \Bigr \} . \end{aligned}$$

To each vertex $$x \in \varLambda $$, we associate a single-particle Hilbert space $$\mathcal {H}_x$$, which we assume to be of finite dimension, $$\sup _{x \in \varLambda } \dim \mathcal {H}_x < \infty $$. Moreover, for each $$X\subset \varLambda $$ let $$\mathcal {H}_X:= \bigotimes _{x \in X} \mathcal {H}_x$$ be the many-particle Hilbert space on X and denote the associated $$C^*$$-algebra of observables by $$\mathcal {A}_{X}:= \mathcal {L}(\mathcal {H}_X)$$. Whenever $X\subset X^{\prime }$, then $$\mathcal {A}_{X}$$ is naturally embedded as a subalgebra of $$\mathcal {A}_{X'}$$ and we set $$\mathcal {A}:= \mathcal {A}_\varLambda $$.

Since a very similar construction is common for fermionic lattice systems (see e.g. [29, 42]), all the results almost immediately translate to this setting.

### Interactions and SLT Operators

An *interaction* is a map

14 $$\begin{aligned} \varPhi :\{ \, X \subset \varLambda \, \} \rightarrow \mathcal {A}, \quad X \mapsto \varPhi (X) = \varPhi (X)^*\in \mathcal {A}_X . \end{aligned}$$

With any interaction, one associates a *sum of local terms (SLT) operator* A via

15 $$\begin{aligned} A := \sum _{X \subset \varLambda } \varPhi (X) \in \mathcal {A}. \end{aligned}$$

Note that, while every interaction defines a unique operator, there are multiple interactions realizing the same operator, i.e. the assignment $\varPhi \mapsto A(\varPhi )$ is not invertible. Note that all interactions and SLT operators are by definition always self-adjoint.

For any b>0 and p∈(0,1], we consider the stretched exponential function

16 $$\begin{aligned} \chi _{b,p} :[0,\infty ) \rightarrow (0,1], \quad x \mapsto \textrm{e}^{-bx^p} \end{aligned}$$

as *localization functions* and define the associated *SLT interaction norm*17on interactions. The quality of the localization for an interaction will be expressed by the finiteness of a norm $\Vert \varPhi \Vert _{b,p}$, *independent* of the (size of the) graph Λ. Operators with this property will often be referred to as (b,p)-*localized SLT operators*.

Moreover, in order to further quantify, how well an interaction is localized in a region $\varOmega \subset \varLambda$, we introduce the *localized SLT interaction norm*18and refer to operators with the property that $\Vert \varPhi \Vert _{b,p; \varOmega } \le C$ as (b,p,Ω)*-localized SLT operators*. Whenever it is clear from the context, or irrelevant for the discussion, we will often also omit the arguments (b,p), and simply refer to Ω*-localized SLT operators*. Finally, observe that $\Vert \varPhi \Vert _{b,p; \varLambda } = \Vert \varPhi \Vert _{b,p}$.

The following simple lemma, whose proof is given in Appendix A.4, relates conceptually easier notions of locality of SLT operators to boundedness of the norm (18).

#### Lemma 2.1

Let A be an SLT operator stemming from an interaction Φ, for which we assume that $\Vert \varPhi \Vert _{b,p} \le C$ for some constants C>0, b>0 and p∈(0,1], and let $\varOmega \subset \varLambda$.

(i) Let A be *strictly* Ω-*localized*, which means Φ(Z)=0 whenever $Z\not \subset \varOmega$. Then it holds that $\Vert \varPhi \Vert _{b,p; \varOmega } \le C$.
(ii) Let A be *strongly* Ω-*localized*, which means Φ(Z)=0 whenever $Z \cap \varOmega = \varnothing$. Then it holds that $\Vert \varPhi \Vert _{b/2,p; \varOmega } \le C$.

## How to Formulate a Local Gap Condition?

Formulating a (i) physically *and* mathematically transparent, (ii) practically applicable, and (iii) reasonably restrictive condition of a *local gap* for a Hamiltonian H is a non-trivial task. In this section, we discuss several different possible approaches to do so. In principle, there are two main ways: In the *extrinsic formulations* we compare H to a globally gapped reference Hamiltonian $H_*$, while the *intrinsic formulations* only involve the original Hamiltonian H alone.

In Sect. 3.1 we introduce the local dynamical gap condition $(\textrm{LDG}_{\textrm{main}})$, that we use for the response theory. It is an intrinsic formulation and allows inverting the Liouvillian in a suitable sense. Afterwards, we list different variants of extrinsic formulations in Sect. 3.2 and other intrinsic formulations in Sect. 3.3 in view of the above listed three requirements. In Sect. 3.4 we explain the relations among the formulations. Finally, in Sect. 3.5 we discuss two exemplary systems, which we prove to have a local dynamical gap in the sense of Assumption $(\textrm{LDG}_{\textrm{main}})$.

Throughout the entire section (unless stated differently), we will use C,ℓ,b,p as generic constants satisfying C>0, $\ell \ge 0$, b>0, and p∈(0,1]. Their precise values might change from line to line and only depend on the model parameters, i.e. the interaction norms (17)–(18) of the involved SLT Hamiltonians, the lattice parameters in (13), or the gap size g>0 of a reference Hamiltonian.

### Local Dynamical Gap

The—at least in view of our application—most important property of an SLT Hamiltonian with a gapped part of its spectrum is that its Liouvillian can be *locally* inverted on the off-diagonal part with respect to its gapped part. To explain this in detail, let us first assume that H is globally spectrally gapped, i.e.

19 $$\begin{aligned} {{\,\textrm{spec}\,}}(H) = \sigma _1 \cup \sigma _2 \qquad \text {with} \qquad d( \sigma _1, \sigma _2 ) \ge g \end{aligned}$$

for some gap size g>0 such that $\sigma _1 \subset I \subset \mathbb {R} {\setminus } \sigma _2$ for some compact interval I. We denote with P the spectral projection associated to H onto $\sigma _1$. Then, there exists an explicit, locality preserving map $\mathcal {I}_{H, g} = \mathcal {I}_{H, g}\llbracket \,\cdot \,\rrbracket :\mathcal {A}\rightarrow \mathcal {A}$, which inverts the Liouvillian $\mathcal {L}_{H}\llbracket \,\cdot \,\rrbracket := -\textrm{i}\, [H, \,\cdot \,]$ in the projection $\langle \,\cdot \,\rangle _{P} = {{\,\textrm{tr}\,}}\bigl ( P \,\cdot \,\bigr )$ onto $\sigma _1$. It is often called *quasi-local inverse of the Liouvillian* and depends on the SLT Hamiltonian H and the gap size g. More precisely, for all A, $B\in \mathcal {A}_\varLambda$ it holds that

20 $$\begin{aligned} \bigl \langle [\mathcal {L}_{H} \circ \mathcal {I}_{H, g} \llbracket A \rrbracket - A , B ] \bigr \rangle _{P} = 0 . \end{aligned}$$

Note that such a map cannot be uniquely characterized as a “weak” inverse of $\mathcal {L}_{H}$, and is thus clearly not unique. However, mostly for concreteness, we will always work with an *explicitly* constructed [1, 24] variant

21 $$\begin{aligned} \mathcal {I}_{H, g}\llbracket A \rrbracket := \int _ \mathbb {R} \textrm{d}t \, w_g(t) \int _0^t \textrm{d}s \, \textrm{e}^{\textrm{i}Hs} \, A \, \textrm{e}^{-\textrm{i}Hs} , \end{aligned}$$

and henceforth called the *quasi-local inverse of the Liouvillian*. See Remarks A.10 and A.12 for relaxed, rather abstract conditions on $\mathcal {I}$. The *filter function* $w_g \in L^1( \mathbb {R} )$ is positive, normalized to $\int w_g = 1$, and required to have Fourier transform^7^$\widehat{w_g}$ with support

22 $$\begin{aligned} {{\,\textrm{supp}\,}}(\widehat{w_g}) \subset [-g,g] . \end{aligned}$$

Moreover, for the explicitly constructed $w_g$, we additionally have the bound

23 $$\begin{aligned} |w_g(t) |\le C \, \textrm{e}^{-|t |^q} \quad \text {for all} \quad q<1 ; \end{aligned}$$

see Lemma A.9 in Appendix A.3. This estimate (23) together with classical Lieb-Robinson bounds [37] for the dynamics generated by H ensure that $\mathcal {I}_{H, g}$ acts as a quasi-local operator. In Appendix A.3 we will briefly recall the construction of $\mathcal {I}_{H,g}$ and report on its properties in more detail.

Interestingly, the spectral gap can be characterized *dynamically* via the quasi-local inverse of the Liouvillian as shown in the next proposition. The proof is given in Sect. 5.1.

#### Proposition 3.1

(Dynamical characterization of a spectral gap) Let H be a self-adjoint operator on a finite-dimensional Hilbert space $\mathcal {H}$. Let g>0, $w_g \in L^1( \mathbb {R} )$ be positive, normalized to $\int w_g = 1$ and satisfy (22) with $\widehat{w_g}\vert _{(-g,g)} > 0$. Decompose the spectrum of H as  and let P be the spectral projection onto $\sigma _1$. Then, we have that

24 $$\begin{aligned} \bigl \langle [\mathcal {L}_{H} \circ \mathcal {I}_{H, g} \llbracket A \rrbracket - A , B ] \bigr \rangle _{P} = 0 \quad \forall A,B \in \mathcal {B}(\mathcal {H}) \quad \iff \quad d( \sigma _1, \sigma _2 ) \ge g . \end{aligned}$$

In many applications the local invertibility of the Liouvillian guarantees that the effect of perturbations remains local. More precisely, it allowed to prove automorphic equivalence of gapped ground states [1, 41] and adiabatic theorems in cases where the perturbation is not allowed to close the gap [3, 40] and later also for gap closing perturbations [25–27, 49].

This motivates the definition of a local gap by weakening (20).

**(LDG**_**main**_**) Local dynamical gap condition** *We say that an equilibrium state* ρ *of the SLT-operator* H, *i.e.* [H,ρ]=0, *is* locally dynamically gapped *of size at least* g>0 *in a region* ${\varLambda ^{\textrm{gap}}}\subset \varLambda$ *with respect to* $C_\textrm{gap}$, b, p>0 *and* $\ell \in \mathbb {N} _0$, *if and only if for all* $X, Y\subset \varLambda$ *and* $A \in \mathcal {A}_X$ *and* $B \in \mathcal {A}_Y$, *it holds that*25⋄

In the special case of ℓ=0, by taking a supremum over all observables B with $\Vert B \Vert \le 1$ in (25), we find that our local dynamical gap condition (25) is actually *equivalent* to

$$\begin{aligned} \bigl \Vert \bigl [ \mathcal {L}_H \circ \mathcal {I}_{H,g}\llbracket A \rrbracket - A, \rho \bigr ] \bigr \Vert _{{{\,\textrm{tr}\,}}} \le C \, \Vert A \Vert \, \chi _{b,p}\bigl ( d( X, \varLambda \setminus {\varLambda ^{\textrm{gap}}}) \bigr ) . \end{aligned}$$

Here, we additionally used cyclicity of the trace together with $\sup _{\Vert D \Vert \le 1} |{{\,\textrm{tr}\,}}( CD ) |= \Vert C \Vert _{{{\,\textrm{tr}\,}}}$.

Moreover, we point out that, while the RHS of (25) is obviously symmetric in A and B, the LHS is as well. This follows by rewriting

26 $$\begin{aligned} \mathcal {L}_H \circ \mathcal {I}_{H,g} \llbracket A \rrbracket - A = - \int _{ \mathbb {R} } \textrm{d}t \, w_g(t) \, \textrm{e}^{\textrm{i}H t}\,A\,\textrm{e}^{-\textrm{i}H t} =: \mathcal {J}_{H,g}\llbracket A \rrbracket \end{aligned}$$

and using [H,ρ]=0 together with the symmetry $w_g(t) = w_g(-t)$.

For the proofs we will use the following slightly weakened, asymmetric version of (25). It can be obtained from $(\textrm{LDG}_{\textrm{main}})$ by absorbing ${{\,\textrm{diam}\,}}(X)$ with the decay in $d( X,\varLambda {\setminus }{\varLambda ^{\textrm{gap}}})$.

**(LDG**_**weak**_**) Local dynamical gap condition—weakened version** *We say that an equilibrium state* ρ *of an SLT operator* H, *i.e. with* [H,ρ]=0, *is* weakly locally dynamically gapped *of size at least* g>0 *in a region* ${\varLambda ^{\textrm{gap}}}\subset \varLambda$ *with respect to* $C_\textrm{gap}$, b, p, β>0 *and* $\ell \in \mathbb {N} _0$, *if and only if the following holds: For all* $X\subset \varLambda$ *satisfying* ${{\,\textrm{diam}\,}}(X) \le d( X, \varLambda {\setminus }{\varLambda ^{\textrm{gap}}})^\beta$ *and observables* $A \in \mathcal {A}_X$, *and* $Y \subset \varLambda$ *and observables* $B \in \mathcal {A}_Y$, *it holds that*

27 $$\begin{aligned} \Bigl |\bigl \langle \bigl [ \mathcal {L}_{H} \circ \mathcal {I}_{H,g}\llbracket A \rrbracket - A , B \bigr ] \bigr \rangle _{\rho } \Bigr |\le C_\textrm{gap}\, \Vert A \Vert \, \Vert B \Vert \, \bigl ( 1+{{\,\textrm{diam}\,}}(Y) \bigr )^\ell \, \chi _{b,p}\bigl ( d( X, \varLambda \setminus {\varLambda ^{\textrm{gap}}}) \bigr ) . \end{aligned}$$

⋄

Finally, we remark that another symmetric (in A and B) bound of the LHS of (25) would be to replace the sum of the distances in the exponent in (25) by $d( X \cup Y, \varLambda {\setminus } {\varLambda ^{\textrm{gap}}})$ (i.e. take the minimum of the distances to $\varLambda {\setminus } {\varLambda ^{\textrm{gap}}}$ instead of their maximum). Using quasi-locality estimates for $\mathcal {J}_{H,g}\llbracket \,\cdot \,\rrbracket$ defined in (26) (see e.g. [44, Lemma 5.1]), this bound can be proven as a consequence of the trace LPPL stated in Sect. 3.2.1, provided that there is a globally spectrally gapped *parent Hamiltonian* (cf. Sect. 3.2) $H_*$ for H. However, as explained there, this is not enough to prove our result with the decay in $d( {\varLambda ^{\textrm{pert}}}, \varLambda {\setminus } {\varLambda ^{\textrm{gap}}})$.

### Extrinsic Local Gap Conditions

In this section, we describe several ways of *extrinsically* expressing that an equilibrium state ρ (e.g. the ground state) of a Hamiltonian H is locally gapped. We call these formulations of a local gap condition *extrinsic*, since the Hamiltonian H and equilibrium state ρ of interest are compared to another reference Hamiltonian $H_*$, called the *parent Hamiltonian*, with equilibrium state $\rho _*$, which is globally gapped. Throughout this section, for simplicity of the presentation, we will assume that both ρ and $\rho _*$ are non-degenerate ground states.

The common core of all the different ways to extrinsically formulate a local gap condition, is to assume that H and $H_*$ differ only locally: That is, $H_*$ and H are related as

28 $$\begin{aligned} H = H_* + J , \end{aligned}$$

where J is some SLT operator, which is localized in $\varLambda {\setminus } {\varLambda ^{\textrm{gap}}}$ (in one of the senses mentioned in Lemma 2.1). One then assumes that also the equilibrium states ρ and $\rho _*$ only differ locally in a suitable sense. Whenever this is the case, we say that ρ and $\rho _*$ satisfy LPPL. This is a slight abuse of nomenclature, as it is usually a property of the Hamiltonian $H_*$, which satisfies the *local perturbations perturb locally (LPPL)* principle if the above holds for a broad class of local perturbations J, which has been shown to be the case for *ground states* in several contexts [2, 15, 28]. In Sect. 3.5 we will also encounter the usual definition, since there we consider $H_*$ and allow the addition of any suitable perturbation J to obtain $H = H_* + J$. But since we only need the relation between two states ρ and $\rho _*$ with Hamiltonians H and $H_*$ for all the following implications, we do not require $H_*$ to satisfy the more general usual LPPL.

#### Trace LPPL

The simplest way to formulate an *extrinsic* local gap condition for H given by (28) with ground state ρ, is to assume that the parent Hamiltonian $H_*$ is globally gapped and that local expectation values in the ground states ρ and $\rho _*$ are almost the same within the gapped region. More precisely, this means that for all $X\subset \varLambda$ and $A \in \mathcal {A}_{X}$

29 $$\begin{aligned} \bigl |\langle A \rangle _{\rho } - \langle A \rangle _{\rho _*} \bigr |\le C \, \Vert A \Vert \, \bigl ( 1+{{\,\textrm{diam}\,}}(X) \bigr )^\ell \, \chi _{b,p}\bigl ( d( X, \varLambda \setminus {\varLambda ^{\textrm{gap}}}) \bigr ) . \end{aligned}$$

Because $|\langle A \rangle _{\rho } - \langle A \rangle _{\rho _*} |= |{{\,\textrm{tr}\,}}( (\rho -\rho _*) \, A ) |$, we call this *trace LPPL*. An estimate of this form has been shown to hold for weakly interacting spin systems in [28] (based on ideas of [50]) and in [2]. Moreover, in [26, Theorem 4.1], such type of assumption was used to formulate an adiabatic theorem for large but finite systems with a gap in the bulk. In this case (cf. [26, eq. (4.1)]), the additional error term (compared to the case of having a global gap) in the response theory expansion (8) becomes $\mathcal {O}\bigl ( \bigl ( \varepsilon ^{C} \, d( X, \varLambda {\setminus } {\varLambda ^{\textrm{gap}}}) \bigr )^{-\infty } \bigr )$. Moreover, using that $H_*$ and J are SLT operators, (29) implies that a condition similar to $(\textrm{LDG}_{\textrm{main}})$ holds *but* with the argument of $\chi _{b,p}$ in (25) being the *minimum* of the distances (or the distance of the *union* X∪Y to $\varLambda {\setminus } {\varLambda ^{\textrm{gap}}}$) instead of their sum (i.e. their maximum). Hence, the decay in $d( {\varLambda ^{\textrm{pert}}}, \varLambda {\setminus } {\varLambda ^{\textrm{gap}}})$ from our main result, emerging in the course of proving Proposition 4.4, will eventually be lost.

In conclusion, although this way of saying that H has a local gap is quite simple, the resulting error terms are considerably bad and, moreover, it does not quite allow for tracking ${d( {\varLambda ^{\textrm{pert}}}, \varLambda {\setminus } {\varLambda ^{\textrm{gap}}})}$-decays.

#### Trace Norm LPPL and Commutator Trace Norm LPPL

One can strengthen the trace LPPL assumption (29) to a *trace norm LPPL*. This means that ρ and $\rho _*$ are not only close in the $\textrm{weak}^*$ sense (29), but instead

30 $$\begin{aligned} \Vert (\rho -\rho _*) \, A \Vert _{\textrm{tr}} + \Vert A \, (\rho -\rho _*) \Vert _{\textrm{tr}} \le C \, \Vert A \Vert \, \bigl ( 1+{{\,\textrm{diam}\,}}(X) \bigr )^\ell \, \chi _{b,p}\bigl ( d( X, \varLambda \setminus {\varLambda ^{\textrm{gap}}}) \bigr ) \end{aligned}$$

for all $X\subset \varLambda$ and $A\in \mathcal {A}_X$, where $\Vert \,\cdot \,\Vert _{\textrm{tr}}:= {{\,\textrm{tr}\,}}( |\,\cdot \,|)$ denotes the trace norm. We remark, that surely (30) implies (29). A somewhat weakened version of (30) is to ask that only the trace norm of the *commutator* $[\rho -\rho _*, A]$ is small

31 $$\begin{aligned} \Vert [\rho -\rho _*, A] \Vert _{\textrm{tr}} \le C \, \Vert A \Vert \, \bigl ( 1+{{\,\textrm{diam}\,}}(X) \bigr )^\ell \, \chi _{b,p}\bigl ( d( X, \varLambda \setminus {\varLambda ^{\textrm{gap}}}) \bigr ) , \end{aligned}$$

**Fig. 3:**
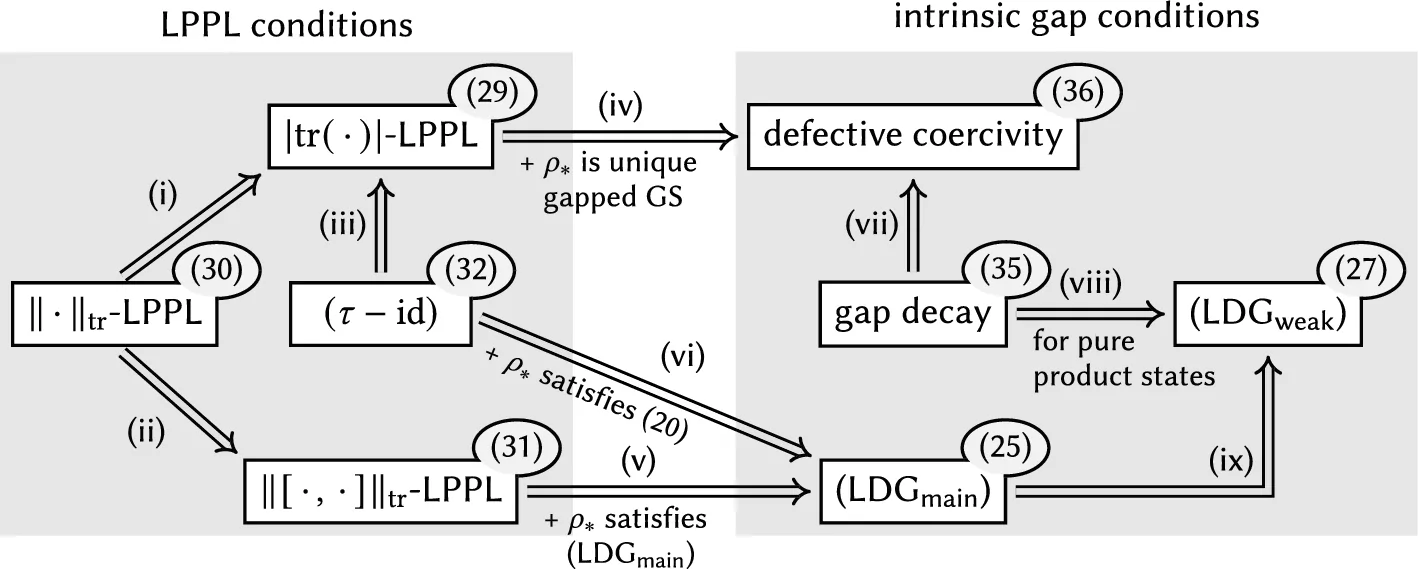
Implications among the various local gap conditions from Sects. 3.1–3.3. The numbering refers to the precise statements in Proposition 3.2

As we show in Proposition 3.2 (see also Fig. 3), both these ways, (30) and (31), of saying that H has a local gap are sufficient to prove that H has a local gap in the sense of Assumption $(\textrm{LDG}_{\textrm{main}})$.^8^ However, it is a quite restrictive condition that ρ and $\rho _*$ are close in such strong topology as (30) or (31).

#### Intertwining Automorphism

Another way of saying that ρ and $\rho _*$ are close to each other, is to assume that there exists an intertwining norm-preserving ∗-automorphism τ on $\mathcal {A}$, i.e. $\langle \,\cdot \,\rangle _\rho = \langle \tau \llbracket \,\cdot \,\rrbracket \rangle _{\rho _*}$, which satisfies

32 $$\begin{aligned} \bigl \Vert ( \tau - {{\,\textrm{id}\,}})\llbracket A \rrbracket \bigr \Vert \le C \, \Vert A \Vert \, \bigl ( 1+{{\,\textrm{diam}\,}}(X) \bigr )^\ell \, \chi _{b,p}\bigl ( d( X,\varLambda \setminus {\varLambda ^{\textrm{gap}}}) \bigr ) , \end{aligned}$$

where ${{\,\textrm{id}\,}}$ is the identity map. Similarly to (30), (32) implies (29). Moreover, (32) is a sufficient condition for having $(\textrm{LDG}_{\textrm{main}})$; see Proposition 3.2. This fact will be used for proving that the examples studied in Sect. 3.5 satisfy $(\textrm{LDG}_{\textrm{main}})$.

### Intrinsic Local Gap Conditions for Ground States

In this section, we discuss other ways of *intrinsically* expressing that a ground state ρ of a Hamiltonian H is locally gapped, in contrast to the local dynamical gap conditions, introduced in Sect. 3.1, they only work for ground states.

In case of ρ being the (unique) *ground state*, a very natural way to connect the notion of locality with spectral analysis is to require that a variational condition characterizing the spectral gap is tested only locally. Generally speaking, assume that $\psi _0$ is the unique ground state with eigenvalue $E_0$ of a local Hamiltonian H in some underlying physical space Λ. Then, a spectral gap above $E_0$ of size (at least) g>0 is characterized by

33 $$\begin{aligned} \inf _{\psi \perp \psi _0} \frac{\langle \psi , (H-E_0) \, \psi \rangle }{\langle \psi , \psi \rangle } \ge g . \end{aligned}$$

If the minimization in (33) is restricted to a smaller set of ψ’s, i.e. those which are localized to a region, say, ${\varLambda ^{\textrm{gap}}}\subset \varLambda$ in some appropriate sense, one could say that H is locally gapped in ${\varLambda ^{\textrm{gap}}}$. Alternatively, for every fixed ψ, the gap size g could be assumed to be non-constant but dependent on the distance of the support of ψ to the region ${\varLambda ^{\textrm{gap}}}$.

For quantum spin systems on the graph Λ, it can easily be checked that the analog of (33) for an SLT Hamiltonian H with unique ground state ρ is

34 $$\begin{aligned} \textrm{i}\, \langle A^* \mathcal {L}_H\llbracket A \rrbracket \rangle _\rho \ge g \, \Bigl ( \langle A^* A \rangle _\rho - \bigl |\langle A \rangle _\rho \bigr |^2 \Bigr ) , \end{aligned}$$

for all observables $A \in \mathcal {A}$, where $\mathcal {L}_H \llbracket \,\cdot \,\rrbracket := -\textrm{i}\, [H, \,\cdot \,]$ denotes the Liouvillian. We now give two options to turn (34) into a local gap condition.

#### Gap Decay

As a first option, one could require that the *gap size* g *decays* as the support X of the observable $A \in \mathcal {A}_X$ approaches the complement of ${\varLambda ^{\textrm{gap}}}$, e.g. as

35 $$\begin{aligned} \textrm{i}\, \langle A^* \mathcal {L}_H\llbracket A \rrbracket \rangle _\rho \ge g \, \Bigl ( 1- C \, \bigl ( 1+{{\,\textrm{diam}\,}}(X) \bigr )^\ell \, \chi _{b,p}\bigl ( d( X, \varLambda \setminus {\varLambda ^{\textrm{gap}}}) \bigr ) \Bigr ) \, \Bigl ( \langle A^* A \rangle _\rho - \bigl |\langle A \rangle _\rho \bigr |^2 \Bigr ) . \end{aligned}$$

In case that $\rho = |\psi \rangle \langle \psi |$ with ψ being a *product state*, variants of Proposition 14 and Lemma 15 in [52] can be used to show the following: The gap decay condition (35) implies the local dynamical gap condition $(\textrm{LDG}_{\textrm{weak}})$; see Proposition 3.2 (viii).

We further remark that, if one is interested in *taking the thermodynamic limit*, $\varLambda \nearrow \varGamma$ for some infinite graph Γ, and ${\varLambda ^{\textrm{gap}}}\nearrow \varGamma$ in this limit, e.g. in the scenario of gap-closing edge modes, then (35) yields a *gap in the bulk* of the naturally associated infinite system [29, Remark 4].^9^

#### Defective Coercivity

Another option is to include a separate *additive* error term on the RHS of (34), e.g. as

36 $$\begin{aligned} \textrm{i}\, \langle A^* \mathcal {L}_H\llbracket A \rrbracket \rangle _\rho \ge g \, \Bigl ( \langle A^* A \rangle _\rho - \bigl |\langle A \rangle _\rho \bigr |^2 \Bigr ) - C \, \Vert A \Vert ^2 \, \bigl ( 1+{{\,\textrm{diam}\,}}(X) \bigr )^\ell \, \chi _{b,p} \bigl ( d( X, \varLambda \setminus {\varLambda ^{\textrm{gap}}}) \bigr ) , \end{aligned}$$

which we call *defective coercivity* for the following reason.

The global gap characterization can equivalently be rewritten as

$$\begin{aligned} \textrm{i}\, \langle \tilde{A}^* \mathcal {L}_H\llbracket \tilde{A} \rrbracket \rangle _\rho \ge g \, \langle \tilde{A}^* \tilde{A} \rangle _\rho \quad \text {with} \quad \tilde{A} := A - \langle A \rangle _\rho , \end{aligned}$$

which means that on $\mathcal {A}^\perp := \{ \, \rho \, \}^\perp = \{ \, A - \langle A \rangle _\rho \,\vert \, A \in \mathcal {A}\, \} \subset \mathcal {A}$ the bounded sesquilinear form

$$\begin{aligned} \mathcal {B}:\mathcal {A}^\perp \times \mathcal {A}^\perp \rightarrow \mathbb {C},\ (A,B) \mapsto \textrm{i}\, \langle A^* \mathcal {L}_H\llbracket B \rrbracket \rangle _\rho \end{aligned}$$

is *coercive* with respect to the semi-norm ${\left| \left| \left| A \right| \right| \right| }:= \sqrt{\langle A^* A \rangle _\rho }$ on $\mathcal {A}^\perp$. Hence, (36) expresses some defect in the original coercivity of (34). We remark that, at least morally, one could use the Lax-Milgram theorem to deduce existence of an inverse of the Liouvillian $\mathcal {L}_H$ given such a coercivity estimate. However, the problem is that the inverse obtained in this way does not necessarily have any nice locality properties, which are crucially used for practical purposes.

Finally, we point out that the defective coercivity (36) for ρ is implied, if there exists a globally gapped parent Hamiltonian $H_*$ with ground state $\rho _*$ that satisfies trace LPPL (29).

### Summary and Comparison

In the previous three sections we described several different ways of expressing that a Hamiltonian is locally gapped, distinguishing between *extrinsic* and *intrinsic* formulations.

While the extrinsic conditions are easy to formulate, they rely on a reference (parent) Hamiltonian with a global gap satisfying some form of the LPPL principle. Since for a system of interest, it is not guaranteed to have such a well-understood parent Hamiltonian available, it is conceptually more desirable to formulate a local gap condition in an intrinsic way. Or, in other words, saying that a system is (or behaves as if it were) *locally* gapped should not only make sense relative to another *globally* gapped system.

In the intrinsic category, we formulated two local *spectral* gap conditions (35) and (36), which are (i) only meaningful for (non-degenerate) ground states and (ii) although mathematically clean, hardly applicable in physical problems in a direct way (apart from the connection in Proposition 3.2 (viii)). These two issues are then resolved by our (intrinsic) local dynamical gap condition (LDG) in (25) and its weakened version in (27).

The following proposition formulates implications between the local gap conditions introduced in the previous sections. The implications are also depicted in Fig. 3. The proof is given in Sect. 5.2.

#### Proposition 3.2

(Relations among the local gap conditions) Fix g>0, $d \in \mathbb {N}$, $C_{\textrm{vol}}> 0$, $\tilde{b} > 0$, $\tilde{p} \in (0,1]$, $C_{\textrm{int}}> 0$. Let $\varLambda \in \mathcal {G}(d, C_{\textrm{vol}})$, ${\varLambda ^{\textrm{gap}}}\subset \varLambda$ and let $H_*$ and J be SLT-operators with corresponding interactions satisfying $\Vert \varPhi _{H_*} \Vert _{\tilde{b},\tilde{p}} < C_{\textrm{int}}$ and $\Vert \varPhi _J \Vert _{\tilde{b},\tilde{p};\varLambda {\setminus } {\varLambda ^{\textrm{gap}}}} < C_{\textrm{int}}$. Let $\rho _*$ be an equilibrium state of $H_*$ and ρ an equilibrium state of $H:= H_* + J$.

Then the following statements hold modulo adjusting the constants C,ℓ,b, and p in a way which only depends on $g,d, C_{\textrm{vol}}, \tilde{b}, \tilde{p}$, and $C_{\textrm{int}}$.

Among the LPPL relations, the following implications hold:

(i) Trace norm LPPL in the sense of (30) implies trace LPPL in the sense of (29).
(ii) Trace norm LPPL in the sense of (30) implies commutator trace norm LPPL in the sense of (31).
(iii) If there exists an intertwining automorphism between ρ and $\rho _*$ such that (32) holds, then ρ and $\rho _*$ satisfy trace LPPL in the sense of (29).

If ρ and $\rho _*$ are non-degenerate ground states and $\rho _*$ satisfies an additional gap condition, then the LPPL conditions imply intrinsic gap conditions:

(iv) If $\rho _*$ is the unique ground state of $H_*$ with a spectral gap of size at least g>0 above its ground state (see (34)) and if $H_*$ satisfies the usual LPPL in the sense of (29), then ρ satisfies defective coercivity (36).
(v) If $\rho _*$ satisfies $(\textrm{LDG}_{\textrm{main}})$ and if $H_*$ satisfies commutator trace norm LPPL in the sense of (31), then ρ also satisfies $(\textrm{LDG}_{\textrm{main}})$. The first assumption is satisfied, for example, if $\rho _*$ is a normalized projection onto a gapped spectral patch of $H_*$; see Proposition 3.1.
(vi) If $\rho _*$ has a dynamical gap in the sense that $\langle [\mathcal {L}_{H_*} \circ \mathcal {I}_{H_*,g}\llbracket A \rrbracket - A,B] \rangle _{\rho _*} = 0$ for all $A,B \in \mathcal {A}$ and if there exists an intertwining automorphism for $\rho _*$ and ρ which satisfies (32), then ρ satisfies $(\textrm{LDG}_{\textrm{main}})$. The first assumption is satisfied, for example, if $\rho _*$ is a normalized projection onto a gapped spectral patch of $H_*$; see Proposition 3.1.

Moreover, the following relations hold among the intrinsic gap conditions:

(vii) A decaying gap size in the sense of (35) implies defective coercivity in the sense of (36).
(viii) If $\rho = |\psi \rangle \langle \psi |$ with ψ a product state and ρ has a decaying gap size in the sense of (35), then ρ satisfies Assumption $(\textrm{LDG}_{\textrm{weak}})$ for any β>0.
(ix) If ρ satisfies Assumption $(\textrm{LDG}_{\textrm{main}})$, then it also satisfies Assumption $(\textrm{LDG}_{\textrm{weak}})$.

### Two Exemplary Systems with a Local Dynamical Gap

In this section, we discuss two exemplary systems, which we show to satisfy the local dynamical gap conditions $(\textrm{LDG}_{\textrm{main}})$ and $(\textrm{LDG}_{\textrm{weak}})$ by means of Proposition 3.2 (vi) and (v), respectively. The first example in Sect. 3.5.1 is concerned with a local perturbation of a classical Ising model. It is contained in a whole class of examples considered in Sect. 3.5.2, which are studied based on [2]. We nevertheless discuss it separately, as proving it to satisfy the local dynamical gap condition $(\textrm{LDG}_{\textrm{main}})$ is elementary, in particular not relying on [2]. The actual proofs for the two examples are given in Sect. 5.3.

#### Perturbations of the Classical Ising Model with Weak Interaction

As the first example we consider the classical Ising model on $\varLambda \subset \mathbb {Z} ^d$

37 $$\begin{aligned} H_* = \sum _{x \in \varLambda } \sigma _x^3 \, + \, \frac{1}{2}\sum _{x, y \in \varLambda } \lambda (x-y) \, \sigma _x^3 \sigma _y^3 , \end{aligned}$$

where $\sigma _x^i$ is the i^th^ Pauli matrix $\sigma ^i$ acting only on the spin on site $x \in \varLambda$. More precisely,

38a $$\begin{aligned} \sigma _x^i = \textbf{1}\otimes \cdots \otimes \textbf{1}\otimes {\underbrace{\sigma ^i}_{\text {site } x }} \otimes \textbf{1}\otimes \cdots \otimes \textbf{1}\in \mathcal {B}\bigl ( \otimes _{z \in \varLambda } \mathcal {H}_z \bigr ) , \end{aligned}$$

where, as usual,

38b $$\begin{aligned} \sigma ^1 := \begin{pmatrix} 0 &  1 \\ 1 &  0 \end{pmatrix} , \quad \sigma ^2 := \begin{pmatrix} 0 &  \textrm{i}\\ - \textrm{i}&  0 \end{pmatrix} , \quad \sigma ^3 := \begin{pmatrix} 1 &  0 \\ 0 & - 1 \end{pmatrix} . \end{aligned}$$

We assume that the coupling function $\lambda :{ \mathbb {Z} ^d}\rightarrow \mathbb {R}$ is symmetric, of finite range, i.e. there exists some R>0 such that λ(x)=0 for |x|>R, and satisfies $\Vert \lambda \Vert _1:= \sum _x |\lambda (x) |< 1$. Clearly, for any p∈(0,1] and b>0, the canonical interaction $\varPhi _{H_*}$ has bounded interaction norm $\Vert \varPhi _{H_*} \Vert _{b,p} \le C_*$ uniformly in Λ.

For these systems we prove the following in Sect. 5.3.1: Let J be a strictly $\varLambda {\setminus } {\varLambda ^{\textrm{gap}}}$-localized SLT perturbation, i.e. J is given by some interaction $\varPhi _J$ satisfying $\Vert \varPhi _J \Vert _{b,p} \le C_J$ for some p∈(0,1], b, $C_J > 0$ and $\varPhi _J(Z)=0$ unless $Z\subset \varLambda {\setminus }{\varLambda ^{\textrm{gap}}}$. Then every ground state ρ of $H = H_* + J$ has a local dynamical gap in the sense of Assumption $(\textrm{LDG}_{\textrm{main}})$.

The example (37) can be generalized to an arbitrary graph $\varLambda \in \mathcal {G}(d, C_{\textrm{vol}})$. Moreover, our above assertions remain valid for any Hamiltonian with gapped on-site terms and sufficiently weak mutually commuting finite range interactions that can be simultaneously diagonalized with the on-site terms (i.e. for *classical* system).

#### Perturbations of Frustration Free Product States

As the second basic example, we consider an SLT Hamiltonian $H_*$ of the form

39 $$\begin{aligned} H_* = \sum _{Z \subset \varLambda } \varPhi (Z) , \end{aligned}$$

for which there exist p∈(0,1] and b>0 and a constant $C_* > 0$ such that $\Vert \varPhi \Vert _{b,p} \le C_*$. Moreover, $\varLambda \in \mathcal {G}(d, C_{\textrm{vol}})$ in (39) is a finite graph as described in Sect. 2. Apart from the locality of the interaction Φ, we will impose the following further conditions (cf. [2]).

**(A1) Frustration-free ground state** *All terms in the SLT Hamiltonian* (39) *are non-negative, i.e.* $\varPhi (Z) \ge 0$ *for all* $Z \subset \varLambda$. *There exists a unique (up to a phase) normalized vector* $\psi _* \in \mathcal {H}$ *such that* $\psi _* \in \ker \varPhi (Z)$ *for all* $Z \subset \varLambda$. *The corresponding ground state (projection) is denoted by* $\rho _* = |\psi _* \rangle \langle \psi _* |$. ⋄

We note that the frustration free assumption depends on the explicit way (39) the Hamiltonian is written, i.e. on the interaction Φ.

**(A2) Product property and regularity** *The vector* $|\psi _* \rangle \in \mathcal {H}$ *factorizes as* $|\psi _* \rangle = \otimes _{z \in \varLambda } |\psi _{*,z} \rangle$ *and for every* $\varOmega \subset \varLambda$, *the unique ground state vector of*

40 $$\begin{aligned} H_*\big \vert _{\varOmega } = \sum _{Z \subset \varOmega } \varPhi (Z) \end{aligned}$$

*is given by* $|\psi _*\vert _{\varOmega } \rangle = \otimes _{z \in \varOmega } |\psi _{*,z} \rangle$. ⋄

The latter condition can be thought of as a strong variant of the common *local topological quantum order (LTQO)*. Moreover, it is possible to relax both, the product property of $|\psi _* \rangle$ and the strong LTQO condition, in the following way: Instead of the product property, we could only assume that $|\psi _* \rangle$ (possibly upon adjoining an auxiliary state after doubling the Hilbert space, see Assumption 4 in [2]) is unitarily conjugate with SLT-generator to a product state. Instead of the strong LTQO property, we could only assume that, upon adjoining a suitable state $|\psi _*\vert _{\varLambda {\setminus }\varOmega } \rangle \in \otimes _{z \in \varLambda {\setminus } \varOmega } \mathcal {H}_z$, the unique ground state $|\psi _*\vert _{\varOmega } \rangle$ of (40) is unitarily conjugate to $|\psi _* \rangle$ in the sense that $|\psi _*\vert _{\varOmega } \rangle \otimes |\psi _*\vert _{\varLambda {\setminus }\varOmega } \rangle = \textrm{e}^{\textrm{i}F} |\psi _* \rangle$, where F is an SLT operator localized at the boundary of Ω. However, we refrain from going into this further generalization of our exemplary system (39) for simplicity.

The final assumption on (39) concerns the spectral gaps

$$\begin{aligned} \gamma := \inf \bigl ( {{\,\textrm{spec}\,}}(H_*) \setminus \{0\} \bigr ) \qquad \text {and} \qquad \gamma (\varOmega ) := \inf \bigl ( {{\,\textrm{spec}\,}}(H_*\big \vert _{\varOmega }) \setminus \{0\} \bigr ) \end{aligned}$$

of $H_*$ and its restrictions $H_*\big \vert _{\varOmega }$ to some $\varOmega \subset \varLambda$, respectively.

**(A3) Gap condition** *The SLT Hamiltonian* $H_*$ *from* (39) *has a spectral gap, i.e.* γ>0. *Moreover, the gap of restrictions* (40) *of* $H_*$ *to* balls $\varOmega = B_r(x)= \{ \, y \in \varLambda \,\vert \, d(x,y) \le r \, \}$ *in* Λ *shrink at most polynomially with the radius, i.e. there exist* $C_\gamma , d_\gamma > 0$ *such that for every* $x \in \varLambda$ *it holds that*

41 $$\begin{aligned} \frac{1}{\gamma (B_r(x))} \le C_\gamma r^{d_\gamma } . \end{aligned}$$

⋄

For these systems we prove the following in Sect. 5.3.2 building on [2]: Let J be a strongly $\varLambda {\setminus } {\varLambda ^{\textrm{gap}}}$-localized SLT perturbation, i.e. J is given by some interaction $\varPhi _J$ satisfying $\Vert \varPhi _J \Vert _{b,p} \le C_J$ for some p∈(0,1], b, $C_J > 0$ and $\varPhi _J(Z)=0$ whenever $Z \cap \bigl ( \varLambda {\setminus } {\varLambda ^{\textrm{gap}}}\bigr ) = \varnothing$. Then every ground state ρ of $H = H_* + J$ has a local dynamical gap in the sense of Assumption $(\textrm{LDG}_{\textrm{weak}})$.

An exemplary system satisfying all of the above assumptions (up to a constant energy shift) is given by the Heisenberg XXZ model for small enough nearest neighbor interactions $|\lambda _1 |$, $|\lambda _3 |$ (depending on the dimension d and the constant $C_{\textrm{vol}}$). The corresponding Hamiltonian is given bywhere E(Λ) denote the edges of Λ and we recall the notations (38).

## Main Result: Response Theory for Locally Gapped Systems

In this section, we formulate our main result, the validity of response theory to any order, Theorem 4.1. Its proof, based on the NEASS construction, is given in Sect. 4.2.

### Response Theory

We recall the assumption $(\textrm{LDG}_{\textrm{main}})$ from Sect. 3.1, which we use to formulate our main result. In a nutshell, it says the following: We have validity of *response theory to all orders* under the assumption of $(\textrm{LDG}_{\textrm{main}})$ for a perturbation localized in ${\varLambda ^{\textrm{pert}}}$—provided that the distance to $\varLambda {\setminus } {\varLambda ^{\textrm{gap}}}$ is sufficiently large compared to $|\log (\varepsilon ) |^{1/q}$, where ε>0 is the strength of the perturbation and q>0 is some small constant.

The formulation of the statement is relatively long in favor of being mathematically precise: We first fix a set of model parameters and expansion orders n, $m \in \mathbb {N}$. Then there exists a constant $C_{n,m}$, depending only on the model parameters and n, $m \in \mathbb {N}$ but being independent of the concrete lattice and Hamiltonian obeying certain bounds in terms of these model parameters. In this way one can for example easily take the thermodynamic limit along a sequence of lattices; see Remark 4.2. A similar philosophy applies to Proposition 4.3 below, too.

The proof of Theorem 4.1 is given at the end of Sect. 4.2.

#### Theorem 4.1

(Response theory to all orders) Let $d\in \mathbb {N}$, $C_{\textrm{vol}}>0$, b>0, p∈(0,1], $C_{\textrm{int}}>0$, g>0, $C_\textrm{gap}>0$, $\ell \in \mathbb {N} _0$, $C_{\textrm{switch}} > 0$, q∈(0,p) and $n,m \in \mathbb {N}$. Then there exists a constant $C_{n,m}> 0$ such that the following holds:

Let $\varLambda \in \mathcal {G}(d,C_{\textrm{vol}})$ (recall (13)), ${\varLambda ^{\textrm{gap}}}$, ${\varLambda ^{\textrm{pert}}}\subset \varLambda$ and $H_0$ and V be SLT-operators, with corresponding interactions that satisfy $\Vert \varPhi _{H_0} \Vert _{b,p}<C_{\textrm{int}}$ and $\Vert \varPhi _{V} \Vert _{b,p;{\varLambda ^{\textrm{pert}}}}<C_{\textrm{int}}$, respectively. Moreover, assume that the equilibrium state $\rho _0$ of $H_0$ is locally dynamically gapped in ${\varLambda ^{\textrm{gap}}}$ of size at least g and with respect to $C_\textrm{gap}$, b, p and ℓ according to Assumption $(\textrm{LDG}_{\textrm{main}})$.

Then, for every $Y\subset \varLambda$ and $B\in \mathcal {A}_Y$ there exist response coefficients $\sigma _{B,j}$ in the following sense: For every ε>0 and smooth switching function $f: \mathbb {R} \rightarrow [ 0,1 ]$ satisfying f(t)=0 for t≤-1, f(t)=1 for t≥0, and $\Vert f \Vert _{C^{\mathfrak {C}_{n,m}}( \mathbb {R} )}<C_{\textrm{switch}}$ with $\mathfrak {C}_{n,m}:= \lceil m(n + 1 +(2d+\ell )/p) \rceil$, consider the time-dependent Hamiltonian

42 $$\begin{aligned} H_\varepsilon (t) := H_0 + \varepsilon \, f(t) \, V . \end{aligned}$$

Let $\rho ^{\varepsilon , \eta , f}(t)$ be the solution of the time-dependent adiabatic Schrödinger equation

43 $$\begin{aligned} \textrm{i}\, \eta \, \frac{\textrm{d}}{\textrm{d}t} \rho ^{\varepsilon , \eta , f}(t) = \bigl [ H_\varepsilon (t) , \rho ^{\varepsilon , \eta , f}(t) \bigr ] \end{aligned}$$

with adiabatic parameter $\eta \in (0,1]$ and initial datum $\rho ^{\varepsilon , \eta , f}(t) = \rho _0$ for all t≤-1.

Then, the response to the perturbation, $\varSigma _B^{\varepsilon , \eta , f}(t):= \langle B \rangle _{\rho ^{\varepsilon , \eta , f}(t)} - \langle B \rangle _{\rho _0}$, satisfies

44 $$\begin{aligned}&\sup _{\eta \in \bigl [ \varepsilon ^m , \varepsilon ^{1/m} \bigr ]} \biggl |\varSigma _B^{\varepsilon , \eta , f}(t) - \sum _{j=1}^{n} \varepsilon ^j \sigma _{B,j} \biggr |\nonumber \\&\quad \le C_{n,m} \, \Vert B \Vert \, \bigl ( 1+{{\,\textrm{diam}\,}}(Y) \bigr )^{(3+n)d+ \ell } \, \bigl ( 1 + t \bigr )^{(2d+\ell )/p + 1} \, \varepsilon ^{n+1}\nonumber \\&\qquad \times \Bigl ( 1 + \textrm{e}^{-d( {\varLambda ^{\textrm{pert}}}, \varLambda \setminus {\varLambda ^{\textrm{gap}}})^{q} - (\mathfrak {C}_{n,m} + 1) \log (\varepsilon )} \Bigr ), \end{aligned}$$

for all t≥0. The first order coefficient is given by *Kubo’s formula* (9).

We chose to formulate our main result (and the three Propositions 4.3–4.5 below, which it relies on) under the slightly stronger but symmetric condition $(\textrm{LDG}_{\textrm{main}})$, although all statements naturally hold under the weakened version $(\textrm{LDG}_{\textrm{weak}})$ as well; see Theorem 6.2 below.

Theorem 4.1 can also be extended to infinite systems.

#### Remark 4.2

*(Extension to infinite systems)* Following the arguments from [27], it is reasonably straightforward to extend our result to the case of infinite systems. More precisely, in order to do so, we need to

- consider Λ to be part of a sequence of graphs exhausting an infinite graph Γ, e.g. $\varLambda \equiv \varLambda _k:= \{-k,\dotsc ,k\}^d \subset \mathbb {Z} ^d$ and $\varGamma := { \mathbb {Z} ^d}$;
- assume that the interactions associated to $H_0$ and V *have a thermodynamic limit* (see [27, Definition 2.1]);
- require that the sequence of equilibrium states $\rho _0 \equiv \rho _0^{\varLambda }$ satisfies the local dynamical gap condition $(\textrm{LDG}_{\textrm{main}})$ with constants independent of Λ and converges (in the $\textrm{weak}^*$ sense in the dual to the algebra of quasi-local observables, see [27, Section 2.5]) as $\varLambda \nearrow \varGamma$;
- and suppose that also the perturbation region ${\varLambda ^{\textrm{pert}}}$ as well as the gapped region ${\varLambda ^{\textrm{gap}}}$ *converge* (in a suitable sense) to some $\varGamma ^{\textrm{pert}}, \varGamma ^\textrm{gap}\subset \varGamma$, respectively, at least ensuring that $d( {\varLambda ^{\textrm{pert}}}, \varLambda {\setminus } {\varLambda ^{\textrm{gap}}}) \rightarrow d( \varGamma ^{\textrm{pert}}, \varGamma {\setminus } \varGamma ^\textrm{gap})$ as $\varLambda \nearrow \varGamma$.

Then, (44) also holds for the infinite system but with $d( \varGamma ^{\textrm{pert}}, \varGamma {\setminus } \varGamma ^\textrm{gap})$ in the exponential.

In the special case, where the gapped region exhausts the entire graph, i.e. ${\varLambda ^{\textrm{gap}}}\nearrow \varGamma$, we find (44) *without* the additional exponential error term. This corresponds to $H_0$ having a *gap in the bulk* (see [26, 29] and also the discussion around (35) in Sect. 3), where in the finite systems $H_0$ could have gap closing edge modes (see Fig. 1).

### Non-equilibrium Almost Stationary States and Proof of Theorem 4.1

The main underlying idea of our justification of linear response theory is the construction of so-called *non-equilibrium almost-stationary states (NEASS)* [25–27, 40, 49] for the dynamics of the perturbed Hamiltonian

45 $$\begin{aligned} H_\varepsilon := H_0 + \varepsilon V . \end{aligned}$$

The proof of Theorem 4.1 is based on three propositions: Proposition 4.3 provides existence of the NEASS, which is a local dressing of the equilibrium state $\rho _0$ of $H_0$, and its almost stationarity under the dynamics generated by (45). Proposition 4.4 shows that adiabatically switching on the perturbation as in (42) drives the equilibrium state $\rho _0$ to a state very close to the NEASS. Finally, Proposition 4.5 provides an explicit formula to approximate expectation values of local operators in the NEASS by expectation values of modified operators in the equilibrium state $\rho _0$.

More precisely, for every $n \in \mathbb {N}$ Proposition 4.3 provides existence of a state $\varPi _n^\varepsilon$, which is obtained from the equilibrium state $\rho _0$ of $H_0$ by a unitary transformation with a small SLT generator, in such a way, that it is almost stationary under the dynamics generated by (45) up to order^10^$\varepsilon ^{n+1}$. This result is the local gap version of [49, Theorem 3.1] and the proof is given in Sect. 6.2.

#### Proposition 4.3

(Non-equilibrium almost-stationary states) Let $d\in \mathbb {N}$, $C_{\textrm{vol}}>0$, b>0, p∈(0,1], $C_{\textrm{int}}>0$ and g>0, $C_\textrm{gap}>0$, $\ell \in \mathbb {N} _0$, q∈(0,p) and $n\in \mathbb {N}$. Then there exists a constant $C_{n}> 0$ such that the following holds:

Let $\varLambda \in \mathcal {G}(d,C_{\textrm{vol}})$ (recall (13)), ${\varLambda ^{\textrm{gap}}}$, ${\varLambda ^{\textrm{pert}}}\subset \varLambda$ and $H_0$ and V be SLT-operators, with corresponding interactions that satisfy $\Vert \varPhi _{H_0} \Vert _{b,p}<C_{\textrm{int}}$ and $\Vert \varPhi _{V} \Vert _{b,p;{\varLambda ^{\textrm{pert}}}}<C_{\textrm{int}}$, respectively. Moreover, assume that the equilibrium state $\rho _0$ of $H_0$ is locally dynamically gapped in ${\varLambda ^{\textrm{gap}}}$ of size at least g and with respect to $C_\textrm{gap}$, b, p and ℓ according to Assumption $(\textrm{LDG}_{\textrm{main}})$.

Then, there exists a sequence $$(A_\mu )_{\mu \in \mathbb {N} }$$ of SLT operators, which are $$(1, p', \varLambda ^{\textrm{pert}})$$-localized for any $p^{\prime }<p$, such that the state

46 $$\begin{aligned} \varPi _n^\varepsilon := \textrm{e}^{\textrm{i}S_n^\varepsilon }\,\rho _0\,\textrm{e}^{-\textrm{i}S_n^\varepsilon } \quad \text {with} \quad S_n^\varepsilon := \sum _{\mu = 1}^n \varepsilon ^{\mu } A_\mu \end{aligned}$$

is almost-stationary for the dynamics generated by $H_\varepsilon = H_0 + \varepsilon V$ in the following sense: Let $\rho ^\varepsilon (t)$ be the solution to the Schrödinger equation

47 $$\begin{aligned} \textrm{i}\frac{\textrm{d}}{\textrm{d}t} \rho ^\varepsilon (t) = \bigl [ H_\varepsilon , \rho ^\varepsilon (t) \bigr ] \quad \text {with} \quad \rho ^\varepsilon (0) = \varPi _n^\varepsilon . \end{aligned}$$

For all $B\in \mathcal {A}_Y$ with $Y\subset \varLambda$ and t≥0, it holds that

48 $$\begin{aligned} \bigl |\langle B \rangle _{\rho ^\varepsilon (t)} - \langle B \rangle _{\varPi _n^\varepsilon } \bigr |&\le C_{n} \, \Vert B \Vert \bigl ( 1+{{\,\textrm{diam}\,}}(Y) \bigr )^{3d+\ell } \, |t |\bigl ( 1 + |t |^{(2d+\ell )/p} \bigr ) \, \varepsilon ^{n+1}\nonumber \\&\quad \times \Bigl ( 1 + \textrm{e}^{-d( {\varLambda ^{\textrm{pert}}}, \varLambda \setminus {\varLambda ^{\textrm{gap}}})^{q} - (n+1) \log (\varepsilon )} \Bigr ). \end{aligned}$$

We point out that, similarly to [49, Theorem 3.1], one can improve the bound in (48) by rescaling every t by $\varepsilon ^m$ for some $m \in \mathbb {N}$, at the cost of increasing the constant in front of $\log (\varepsilon )$.

Next, Proposition 4.4 states that the NEASS constructed in Proposition 4.3 is approached under the dynamics generated by the time-dependent Hamiltonian (42) whose perturbation V is turned on by the switching function f on the adiabatic timescale 1/η as in (43). It is the local gap version of [49, Proposition 3.2] and the proof is given in Sect. 6.2.

#### Proposition 4.4

(Adiabatic switching and the NEASS) Under the assumptions of Theorem 4.1 (in particular recalling (43)) it holds that

49 $$\begin{aligned} \bigl |\langle B \rangle _{\rho ^{\varepsilon , \eta , f}(t)} - \langle B \rangle _{\varPi _n^\varepsilon } \bigr |&\le C \, \Vert B \Vert \bigl ( 1+{{\,\textrm{diam}\,}}(Y) \bigr )^{3d+\ell } \bigl ( 1 + t \bigr )^{(2d+\ell )/p+1} \, \varepsilon \, \frac{\varepsilon ^{n} + \eta ^{n}}{\eta ^{(2d+\ell )/p+1}}\nonumber \\&\quad \times \biggl ( 1 + \textrm{e}^{-d( {\varLambda ^{\textrm{pert}}}, \varLambda \setminus {\varLambda ^{\textrm{gap}}})^{q} - (n+1) \log (\varepsilon )} \biggr ), \end{aligned}$$

for t≥0, where $\varPi _n^\varepsilon$ is the NEASS (46) constructed in Proposition 4.3.

For our application to response theory, it is important to have an explicit expansion of expectation values in the NEASS in powers of ε with coefficients given by expectations in the unperturbed equilibrium state, the linear term constituting the celebrated *Kubo formula*. This is the content of the following proposition, which is the local gap version of [49, Proposition 3.1], and whose proof is given in Sect. 6.2.

#### Proposition 4.5

(Asymptotic expansion of the NEASS) Under the assumptions of Proposition 4.3, there exist linear maps $\mathcal {K}_j :\mathcal {A}\rightarrow \mathcal {A}$, $j \in \mathbb {N}$, given by nested commutators with the $A_\mu$’s in (46), such that for any m≤n it holds that

50 $$\begin{aligned} \biggl |\langle B \rangle _{\varPi _n^\varepsilon } - \sum _{j=0}^{m} \varepsilon ^j \langle \mathcal {K}_j\llbracket B \rrbracket \rangle _{\rho _0} \biggr |\le C_{n} \, \varepsilon ^{m+1} \, \Vert B \Vert \, |Y |^{m+1} \, \chi _{1,p'}\bigl ( d( {\varLambda ^{\textrm{pert}}},Y ) \bigr ) , \end{aligned}$$

with $p^{\prime }$ the parameter of the localization of the $$A_\mu $$ given in Proposition 4.3. The first two orders of the expansion (50) are explicitly given by

51 $$\begin{aligned} \langle \mathcal {K}_0\llbracket B \rrbracket \rangle _{\rho _0} = \langle B \rangle _{\rho _0} \quad \text {and}\quad \langle \mathcal {K}_1\llbracket B \rrbracket \rangle _{\rho _0} = - \textrm{i}\left\langle \bigl [ \mathcal {I}_{H_0,g}\llbracket V \rrbracket , B \bigr ] \right\rangle _{\rho _0} , \end{aligned}$$

where $\mathcal {I}_{H_0,g}\llbracket \,\cdot \,\rrbracket$ is the inverse Liouvillian from (21).

We can finally give the proof of Theorem 4.1.

#### Proof of Theorem 4.1

Armed with Propositions 4.4–4.5, the proof of Theorem 4.1 follows by a simple application of the triangle inequality when applying Proposition 4.4 with $n \rightarrow \mathfrak {C}_{n,m}$ and Proposition 4.5 for $n \rightarrow \mathfrak {C}_{n,m}$ and m→n as in [49, Theorem 4.1]. □

## Local Gap Conditions: Proofs for Sect. 3

This section collects several proofs concerning the local dynamical gap condition $(\textrm{LDG}_{\textrm{main}})$ from Sect. 3.1, which were skipped in earlier sections. More precisely, we will prove the dynamical characterization of a spectral gap in Proposition 3.1 and the relations among the various local gap conditions formulated in Proposition 3.2. Finally, we show that the examples from Sect. 3.5 satisfy $(\textrm{LDG}_{\textrm{main}})$ by means of Proposition 3.2.

### Dynamical Characterization of a Spectral Gap: Proof of Proposition 3.1

Deriving the LHS from the RHS is standard material, see e.g. [44, Lemma 6.8 and Proposition 6.9]. For the other direction, recall (26) and suppose that for all $A,B \in \mathcal {B}(\mathcal {H})$

$$\begin{aligned} 0 = \bigl \langle \bigl [ \mathcal {L}_H \circ \mathcal {I}_{H,g}\llbracket A \rrbracket - A, B \bigr ] \bigr \rangle _P = \bigl \langle \bigl [ \mathcal {J}_{H,g}\llbracket A \rrbracket ,B \bigr ] \bigr \rangle _{P} = - {{\,\textrm{tr}\,}}\bigl ( \bigl [ \mathcal {J}_{H,g}\llbracket A \rrbracket , P \bigr ] \, B \bigr ) . \end{aligned}$$

Since B is arbitrary, this means $[\mathcal {J}_{H,g}\llbracket A \rrbracket , P] = 0$. Moreover, inserting the spectral decomposition $H = \sum _{n} E_n \, P_n$, this can be written as52For *contradiction*, we now assume that $d( \sigma _1, \sigma _2 ) < g$. Then, since $\widehat{w_g}\vert _{(-g,g)} > 0$, one can easily construct an observable A, which violates (52), e.g. $A = |\phi _{n_*} \rangle \langle \phi _{m_*} |$ where $\phi _{n_*}$ and $\phi _{m_*}$ are eigenvectors of H with eigenvalues $E_{n_*} \in \sigma _1$, $E_{m_*} \in \sigma _2$, respectively, satisfying $|E_{n_*} - E_{m_*} |= d( \sigma _1, \sigma _2 ) < g$.

### Relations Among Local Gap Conditions: Proof of Proposition 3.2

We prove the nine implications gathered in Proposition 3.2 one by one. Unless differently stated, we will use the constants C,ℓ,b and p from the formulation of Proposition 3.2 generically, i.e. their precise value might change from line to line. Some technical details are kept brief in this section, more detailed proofs using similar arguments are given in Appendix A.

Proofs of (ii), (i), (iii): All of these are obvious, by application of the estimates

$$\begin{aligned} \Vert [\rho -\rho _*, A] \Vert _{\textrm{tr}}&\le \Vert (\rho -\rho _*) \, A \Vert _{\textrm{tr}} + \Vert A \, (\rho -\rho _*) \Vert _{\textrm{tr}}, \\ \bigl |{{\,\textrm{tr}\,}}\bigl ( (\rho -\rho _*)A \bigr ) \bigr |&\le \Vert (\rho -\rho _*) A \Vert _{\textrm{tr}}, \end{aligned}$$

and

$$\begin{aligned} \bigl |{{\,\textrm{tr}\,}}\bigl ( \rho _* ( \tau - {{\,\textrm{id}\,}})\llbracket A \rrbracket \bigr ) \bigr |&\le \bigl \Vert ( \tau - {{\,\textrm{id}\,}})\llbracket A \rrbracket \bigr \Vert , \end{aligned}$$

for (ii), (i) and (iii), respectively.

Proof of (iv): By assumption, it holds that

53 $$\begin{aligned} \textrm{i}\, \langle A^* \mathcal {L}_{H_*}\llbracket A \rrbracket \rangle _{\rho _*} \ge g \, \Bigl ( \langle A^* A \rangle _{\rho _*} - \bigl |\langle A \rangle _{\rho _*} \bigr |^2 \Bigr ) \end{aligned}$$

for all observables $A \in \mathcal {A}$, where $\mathcal {L}_{H_*} \llbracket \,\cdot \,\rrbracket := -\textrm{i}\, [H_*, \,\cdot \,]$ denotes the Liouvillian of $H_*$. The idea is now to replace $H_* \rightarrow H$ and $\rho _* \rightarrow \rho$ in (53) and estimate the resulting error in such a way that we arrive at (36).

First, by application of (29), we replace $\bigl ( \langle A^* A \rangle _{\rho _*} - |\langle A \rangle _{\rho _*} |^2 \bigr )$ by $\bigl ( \langle A^* A \rangle _{\rho } - |\langle A \rangle _{\rho } |^2 \bigr )$ on the RHS of (53) at the cost of an error bounded by $C \, \Vert A \Vert ^2 \bigl ( 1+{{\,\textrm{diam}\,}}(X) \bigr )^\ell \, \chi _{b,p}\bigl ( d( X, \varLambda {\setminus } {\varLambda ^{\textrm{gap}}}) \bigr )$.

For the LHS of (53), we estimate

54 $$\begin{aligned} \begin{aligned} \hspace{2em}&\hspace{-2em} \Bigl |\bigl \langle A^* \mathcal {L}_{H_*}\llbracket A \rrbracket \bigr \rangle _{\rho _*} - \bigl \langle A^* \mathcal {L}_{H}\llbracket A \rrbracket \bigr \rangle _{\rho } \Bigr |\\&\le \Bigl |\bigl \langle A^* \bigl ( \mathcal {L}_{H_*}\llbracket A \rrbracket - \mathcal {L}_H\llbracket A \rrbracket \bigr ) \bigr \rangle _{\rho _*} \Bigr |+ \bigl |{{\,\textrm{tr}\,}}\bigl ( (\rho _* - \rho ) A^* \mathcal {L}_{H}\llbracket A \rrbracket \bigr ) \bigr |\end{aligned} \end{aligned}$$

by means of the triangle inequality. The first term on the RHS of (54) can now be bounded as (recall that $H = H_* + J$, J is $\varLambda {\setminus } {\varLambda ^{\textrm{gap}}}$-localized, and $A \in \mathcal {A}_X$)

55 $$\begin{aligned} \Bigl |\bigl \langle A^* \bigl ( \mathcal {L}_{H_*}\llbracket A \rrbracket - \mathcal {L}_H\llbracket A \rrbracket \bigr ) \bigr \rangle _{\rho _*} \Bigr |\le 2 \, \Vert \varPhi _J \Vert _{b,p;\varLambda \setminus {\varLambda ^{\textrm{gap}}}} \, \Vert A \Vert ^2 \, \bigl ( 1+{{\,\textrm{diam}\,}}(X) \bigr )^d \, \chi _{b,p}\bigl ( d( X, \varLambda \setminus {\varLambda ^{\textrm{gap}}}) \bigr ) \end{aligned}$$

by application of (91) in Lemma A.4.

For the second term on the RHS of (54), we write $\mathcal {L}_H\llbracket A \rrbracket = \mathbb {E}_{X_n} \mathcal {L}_H\llbracket A \rrbracket + ({{\,\textrm{id}\,}}- \mathbb {E}_{X_n} ) \mathcal {L}_H\llbracket A \rrbracket$ for some n to be chosen below, where $X_n:= \{ \, x \in \varLambda \,\vert \, d( x,X ) \le n \, \}$ denotes the n-fattening of the set $X \subset \varLambda$. We now estimate the two terms separately. For the first term, we employ (29) to bound

56 $$\begin{aligned} \bigl |{{\,\textrm{tr}\,}}\bigl ( (\rho _* - \rho ) \, A^* \, \mathbb {E}_{X_n} \mathcal {L}_H\llbracket A \rrbracket \bigr ) \bigr |\le C \, \Vert A \Vert ^2 \bigl ( 1+{{\,\textrm{diam}\,}}(X) + n \bigr )^\ell \chi _{b,p}\bigl ( d( X_n, \varLambda \setminus {\varLambda ^{\textrm{gap}}}) \bigr ) \end{aligned}$$

where we used that $\mathbb {E}_{X_n} \mathcal {L}_H\llbracket A \rrbracket \in \mathcal {A}_{X_n}$ (by definition) and $\Vert \mathbb {E}_{X_n} \mathcal {L}_H\llbracket A \rrbracket \Vert \le C \, |X |\, \Vert A \Vert$ (by (91) from Lemma A.4 and Lemma A.6 (c)). For the second term, we use (92) from Lemma A.4 and Lemma A.6 (e) for estimating the difference $\Vert ({{\,\textrm{id}\,}}- \mathbb {E}_{X_n} ) \mathcal {L}_H\llbracket A \rrbracket \Vert$ to get

57 $$\begin{aligned} \bigl |{{\,\textrm{tr}\,}}\bigl ( (\rho _* - \rho ) \, A^* \, ({{\,\textrm{id}\,}}- \mathbb {E}_{X_n} ) \, \mathcal {L}_H\llbracket A \rrbracket \bigr ) \bigr |\le C \, \Vert A \Vert ^2 \, \bigl ( 1+{{\,\textrm{diam}\,}}(X) \bigr )^\ell \, \chi _{b,p}(n) . \end{aligned}$$

Using $d( X_n, \varLambda {\setminus } {\varLambda ^{\textrm{gap}}}) \ge d( X, \varLambda {\setminus } {\varLambda ^{\textrm{gap}}}) - n$ for (56), we can pick $n = d( X, \varLambda {\setminus } {\varLambda ^{\textrm{gap}}})/2$, say, to estimate

$$\begin{aligned} {(56)} + {(57)} \le C \, \Vert A \Vert ^2 \, \bigl ( 1+{{\,\textrm{diam}\,}}(X) \bigr )^\ell \, \chi _{b/2,p}\bigl ( d( X, \varLambda \setminus {\varLambda ^{\textrm{gap}}}) \bigr ) . \end{aligned}$$

Combining this with (55), we estimate (54) by $C \, \Vert A \Vert ^2 \bigl ( 1+{{\,\textrm{diam}\,}}(X) \bigr )^\ell \, \chi _{b,p}\bigl ( d( X, \varLambda {\setminus } {\varLambda ^{\textrm{gap}}}) \bigr )$ and we thus arrive at (36).

Proof of (vii): This is obvious, because $\bigl |\langle A^* A \rangle _{\rho } - \bigl |\langle A \rangle _{\rho } \bigr |^2 \bigr |\le 2 \Vert A \Vert ^2$.

Proof of (v): By assumption (25), it holds that, for all observables $A \in \mathcal {A}_X$ and $B \in \mathcal {A}_Y$ localized in $X\subset \varLambda$ and $Y \subset \varLambda$ (recall (26) for the definition of $\mathcal {J}_{H_*,g}$)58Similarly to the proof of (iv), the idea is now to replace $H_* \rightarrow H$ and $\rho _* \rightarrow \rho$ on the LHS of (58) at the price of an error that is bounded in terms of the RHS of (58). In order to do so, we will heavily exploit the symmetry of (58) in A and B (recall the discussion around (26)). That is, we will prove the bound first only with $d( X, \varLambda {\setminus } {\varLambda ^{\textrm{gap}}})$ in the argument of $\chi _{b,p}$ and later obtain their sum (like on the RHS of (58)) by symmetry (modulo changing b→b/2).

To begin with, by the triangle inequality, we have

59 $$\begin{aligned} \begin{aligned} \hspace{2em}&\hspace{-2em} \Bigl |\bigl \langle \bigl [ \mathcal {J}_{H_*,g}\llbracket A \rrbracket , B \bigr ] \bigr \rangle _{\rho _*} - \bigl \langle \bigl [ \mathcal {J}_{H,g}\llbracket A \rrbracket , B \bigr ] \bigr \rangle _{\rho } \Bigr |\\  &\le \Bigl |\bigl \langle \bigl [ ( \mathcal {J}_{H_*,g} - \mathcal {J}_{H,g} )\llbracket A \rrbracket , B \bigr ] \bigr \rangle _{\rho } \Bigr |+ \bigl |{{\,\textrm{tr}\,}}\bigl ( (\rho - \rho _*) \bigl [ B , \mathcal {J}_{H_*,g}\llbracket A \rrbracket \bigr ] \bigr ) \bigr |. \end{aligned} \end{aligned}$$

For the first term on the RHS of (59), we estimate

60 $$\begin{aligned} \bigl \Vert ( \mathcal {J}_{H_*,g} - \mathcal {J}_{H,g} )\llbracket A \rrbracket \bigr \Vert \le \int _{ \mathbb {R} } \textrm{d}t \, w_g(t) \, \Vert \textrm{e}^{\textrm{i}t H_*} A \textrm{e}^{-\textrm{i}t H_*} - \textrm{e}^{\textrm{i}t H} A \textrm{e}^{-\textrm{i}t H} \Vert . \end{aligned}$$

Recalling $H = H_* + J$, the difference between the two time evolutions can be written as

$$\begin{aligned} \textrm{e}^{\textrm{i}t H_*} \, A \, \textrm{e}^{-\textrm{i}t H_*} - \textrm{e}^{\textrm{i}t H} \, A \, \textrm{e}^{-\textrm{i}t H} = - \textrm{i}\int _0^t \textrm{d}s \, \textrm{e}^{\textrm{i}t H} \, \bigl [ J , \textrm{e}^{\textrm{i}s H_*} \, A \, \textrm{e}^{-\textrm{i}s H_*} \bigr ] \, \textrm{e}^{-\textrm{i}t H} . \end{aligned}$$

We thus find that

61 $$\begin{aligned} \begin{aligned} {(60)}&\le \int _{ \mathbb {R} } \textrm{d}t \, w_g(t) \, |t |\, \sup _{s\in [ 0,t ]} \bigl \Vert \bigl [ J , \textrm{e}^{\textrm{i}s H_*} \, A \, \textrm{e}^{-\textrm{i}s H_*} \bigr ] \bigr \Vert \\  &\le C \, \Vert A \Vert \, \bigl ( 1+{{\,\textrm{diam}\,}}(X) \bigr )^{2d} \begin{aligned} \biggl (&\chi _{b,p}\bigl ( d( X, \varLambda \setminus {\varLambda ^{\textrm{gap}}}) \bigr ) \int _{I} \textrm{d}t \, w_g(t) \, |t |\, \bigl ( 1 + |t |\bigr )^{d/p} \\  &+ \int _{ \mathbb {R} \setminus I} \textrm{d}t \, w_g(t) \, |t |\, \bigl ( 1 + |t |\bigr )^{d/p} \biggr ) \end{aligned} \\  &\le C \, \Vert A \Vert \, \bigl ( 1+{{\,\textrm{diam}\,}}(X) \bigr )^{2d} \, \chi _{b,p}\bigl ( d( X, \varLambda \setminus {\varLambda ^{\textrm{gap}}}) \bigr ) , \end{aligned} \end{aligned}$$

where we denoted $I:= \bigl \{ \, t \in \mathbb {R} \,\big \vert \, |t |\le \bigl ( d( X, \varLambda {\setminus } {\varLambda ^{\textrm{gap}}})/(2v) \bigr )^p/2 \, \bigr \}$. Here, v is the Lieb-Robinson velocity from Lemma A.7, which we employed in the second step. In the final step, we used the stretched exponential decay of $w_g$ from (23) and possibly adjusted the constants C, b, and p.

The second term on the RHS of (59) can be estimated by means of (31), since

$$\begin{aligned} \bigl |{{\,\textrm{tr}\,}}\bigl ( (\rho - \rho _*) \, \bigl [ B , \mathcal {J}_{H_*,g}\llbracket A \rrbracket \bigr ] \bigr ) \bigr |\le \Vert B \Vert \, \bigl \Vert \bigl [ \rho - \rho _* , \mathcal {J}_{H_*,g}\llbracket A \rrbracket \bigr ] \bigr \Vert _{{{\,\textrm{tr}\,}}} . \end{aligned}$$

For $\mathcal {J}_{H_*,g}\llbracket A \rrbracket$ we now apply the local decomposition technique, analogously to the arguments around (56)–(57). More precisely, taking $A \in \mathcal {A}_X$, we now write $\mathcal {J}_{H_*,g}\llbracket A \rrbracket = \mathbb {E}_{X_n} \mathcal {J}_{H_*,g}\llbracket A \rrbracket + ({{\,\textrm{id}\,}}- \mathbb {E}_{X_n} ) \mathcal {J}_{H_*,g}\llbracket A \rrbracket$ for some n to be chosen below, where $X_n:= \{ \, x \in \varLambda \,\vert \, d( x,X ) \le n \, \}$ denotes the n-fattening of the set $X \subset \varLambda$. We now estimate the two terms separately. For the first term, we employ (31) to bound

62 $$\begin{aligned} \bigl \Vert \bigl [ \rho - \rho _* , \mathbb {E}_{X_n}\mathcal {J}_{H_*,g}\llbracket A \rrbracket \bigr ] \bigr \Vert _{{{\,\textrm{tr}\,}}} \le C \, \Vert A \Vert \, \bigl ( 1+{{\,\textrm{diam}\,}}(X) + n \bigr )^\ell \, \chi _{b,p}\bigl ( d( X_n, \varLambda \setminus {\varLambda ^{\textrm{gap}}}) \bigr ) \end{aligned}$$

where we used that $\mathbb {E}_{X_n} \mathcal {J}_{H_*,g}\llbracket A \rrbracket \in \mathcal {A}_{X_n}$ (by definition) and $\Vert \mathbb {E}_{X_n} \mathcal {J}_{H_*,g}\llbracket A \rrbracket \Vert \le \Vert A \Vert$. For the second term, we simply use Lemma A.6 together with (100) applied with $\mathcal {I}\rightarrow \mathcal {J}$ for estimating the difference $\Vert ({{\,\textrm{id}\,}}- \mathbb {E}_{X_n} ) \mathcal {J}_{H_*,g}\llbracket A \rrbracket \Vert$ to get

63 $$\begin{aligned} \begin{aligned} \hspace{2em}&\hspace{-2em} \bigl \Vert \bigl [ \rho - \rho _* , ({{\,\textrm{id}\,}}- \mathbb {E}_{X_n})\mathcal {J}_{H_*,g}\llbracket A \rrbracket \bigr ] \bigr \Vert _{{{\,\textrm{tr}\,}}} \\  &\le 2\Vert \rho - \rho _* \Vert _{{{\,\textrm{tr}\,}}} \, \Vert ({{\,\textrm{id}\,}}- \mathbb {E}_{X_n})\mathcal {J}_{H_*,g}\llbracket A \rrbracket \Vert \le C \, \Vert A \Vert \, \bigl ( 1+{{\,\textrm{diam}\,}}(X) \bigr )^\ell \, \chi _{b,p}(n) . \end{aligned} \end{aligned}$$

Using $d( X_n, \varLambda {\setminus } {\varLambda ^{\textrm{gap}}}) \ge d( X, \varLambda {\setminus } {\varLambda ^{\textrm{gap}}}) - n$ for (62), we can pick $n = d( X, \varLambda {\setminus } {\varLambda ^{\textrm{gap}}})/2$, say, to estimate

$$\begin{aligned} {(62)} + {(63)} \le C \, \Vert A \Vert \, \bigl ( 1+{{\,\textrm{diam}\,}}(X) \bigr )^\ell \, \chi _{b/2,p}\bigl ( d( X, \varLambda \setminus {\varLambda ^{\textrm{gap}}}) \bigr ) . \end{aligned}$$

Finally, as mentioned above, interchanging the roles of A and B (by symmetry of the original expression; recall the discussion around (26)), we thus arrive at a bound on $\bigl |\bigl \langle \bigl [ \mathcal {J}_{H,g}\llbracket A \rrbracket , B \bigr ] \bigr \rangle _{\rho } \bigr |$ of the form given by the RHS of (58) (modulo changing b→b/2). Therefore, combining (58) with (59), and (60)–(61) as well as (62)–(63), we conclude the desired.

Proof of (vi): Instead of (58), we start with (by assumption)

64 $$\begin{aligned} \bigl \langle \bigl [ \mathcal {J}_{H_*,g}\llbracket A \rrbracket , B \bigr ] \bigr \rangle _{\rho _*} = 0 \qquad \text {for all} \qquad A,B \in \mathcal {A}. \end{aligned}$$

Apart from this, the idea is identical to (iv) and (v). Hence, by means of the triangle inequality, we obtain the same two terms from (59). The first term can be estimated in exactly the same way as in (60)–(61). The second term in (59) has to be treated a bit differently as in (62)–(63), since we now assumed (32) instead of (31).

In fact, for this term, using $\langle \,\cdot \,\rangle _\rho = \langle \tau \llbracket \,\cdot \,\rrbracket \rangle _{\rho _*}$, that τ is a ∗-automorphism, and (64), we get

$$\begin{aligned} \bigl |{{\,\textrm{tr}\,}}\bigl ( (\rho - \rho _*) \bigl [ B , \mathcal {J}_{H_*,g}\llbracket A \rrbracket \bigr ] \bigr ) \bigr |&= \bigl |{{\,\textrm{tr}\,}}\bigl ( \rho _* \bigl [ \tau \llbracket B \rrbracket , (\tau -{{\,\textrm{id}\,}})\circ \mathcal {J}_{H_*,g}\llbracket A \rrbracket \bigr ] \bigr ) \bigr |\\&\le 2 \, \Vert B \Vert \, \Vert (\tau -{{\,\textrm{id}\,}})\circ \mathcal {J}_{H_*,g}\llbracket A \rrbracket \Vert . \end{aligned}$$

This can now be treated exactly as done in the argument around (62)–(63) (i.e. taking $A \in \mathcal {A}_X$, writing $\mathcal {J}_{H_*,g}\llbracket A \rrbracket = \mathbb {E}_{X_n} \mathcal {J}_{H_*,g}\llbracket A \rrbracket + ({{\,\textrm{id}\,}}- \mathbb {E}_{X_n} ) \mathcal {J}_{H_*,g}\llbracket A \rrbracket$, and estimating the two terms separately while optimizing in n).

Proof of (viii): Without loss of generality, we may assume that $X \subset \varLambda$ in (35) is such that $C \, \bigl ( 1+{{\,\textrm{diam}\,}}(X) \bigr )^\ell \chi _{b, p}\bigl ( d( X, \varLambda {\setminus } {\varLambda ^{\textrm{gap}}}) \bigr ) < 1/2$, say (otherwise there is nothing to prove), and ${{\,\textrm{diam}\,}}(X)\le d( X, \varLambda {\setminus } {\varLambda ^{\textrm{gap}}})^\beta$ for some β>0. By assumption, for such $X \subset \varLambda$ and $A \in \mathcal {A}_X$, we have

65 $$\begin{aligned} \textrm{i}\langle \psi , A^* \mathcal {L}_H\llbracket A \rrbracket \psi \rangle \ge \frac{g}{2} \, \Bigl ( \langle \psi , A^* A \psi \rangle - \bigl |\langle \psi , A \psi \rangle \bigr |^2 \Bigr ) \quad \text {with}\quad |\psi \rangle = \otimes _{x \in \varLambda } |\psi _x \rangle , \ \Vert \psi _x \Vert = 1 . \end{aligned}$$

Our claim will be a consequence of the following lemma.

#### Lemma 5.1

(cf. Proposition 14 in [52]) Take a Hermitian $A \in \mathcal {A}_X$ as above and assume (65). Denote the n-fattening of X by $X_n:= \{ \, z \in \varLambda \,\vert \, d( z, X ) \le n \, \}$. Then, there exists a Hermitian operator $\widetilde{A} \in \mathcal {A}_{X_n}$ with $n:= \lfloor d( X, \varLambda {\setminus } {\varLambda ^{\textrm{gap}}}) /2 \rfloor$ such that ψ is an eigenvector of $\widetilde{A}$, i.e. $\widetilde{A} \psi = \widetilde{E} \psi$ for some $\widetilde{E} \in \mathbb {R}$, and we have the bound

66 $$\begin{aligned} \bigl \Vert \mathbb {E}_{X_n} \mathcal {J}_{H,g}\llbracket A \rrbracket - \widetilde{A} \bigr \Vert \le C \, \Vert A \Vert \chi _{b,p}\bigl ( d( X, \varLambda \setminus {\varLambda ^{\textrm{gap}}}) \bigr ) \end{aligned}$$

with $\mathcal {J}_{H,g}$ being defined in (26).

Armed with Lemma 5.1, we now turn to estimating the LHS of (27), which is given by $\langle [\mathcal {J}_{H,g} \llbracket A \rrbracket , B] \rangle _\rho = \langle \psi , [\mathcal {J}_{H,g} \llbracket A \rrbracket , B] \psi \rangle$. By the triangle inequality, we have

67 $$\begin{aligned} \begin{aligned} \hspace{2em}&\hspace{-2em} \bigl |\big \langle \psi , [\mathcal {J}_{H,g}\llbracket A \rrbracket , B] \psi \big \rangle \bigr |\\  &\le \bigl |\big \langle \psi , [({{\,\textrm{id}\,}}- \mathbb {E}_{X_n})\mathcal {J}_{H,g}\llbracket A \rrbracket , B] \psi \big \rangle \bigr |+ \bigl |\big \langle \psi , [\mathbb {E}_{X_n}\mathcal {J}_{H,g}\llbracket A \rrbracket - \widetilde{A}, B] \psi \big \rangle \bigr |+ \bigl |\big \langle \psi , [\widetilde{A}, B] \psi \big \rangle \bigr |\end{aligned} \end{aligned}$$

and estimate the three terms separately. The first term in (67) can be treated as in (63), yielding the bound

68 $$\begin{aligned} C \, \Vert A \Vert \, \Vert B \Vert \, \chi _{b,p}\bigl ( d( X, \varLambda \setminus {\varLambda ^{\textrm{gap}}}) \bigr ) , \end{aligned}$$

where we additionally used that ${{\,\textrm{diam}\,}}(X)\le d( X, \varLambda {\setminus } {\varLambda ^{\textrm{gap}}})^\beta$. For the second term, we employ (66), yielding the same bound as for the first term. The third term in (67) vanishes since $\widetilde{A} \psi = \widetilde{E} \psi$. Therefore, (67) is bounded by (68) and we have proven Proposition 3.2 (viii).

It thus remains to give the proof of Lemma 5.1.

#### Proof of Lemma 5.1

The principal idea is similar to [52, Proposition 14]. To start with, we assume w.l.o.g. that Hψ=0, i.e. ψ is an eigenvector to the eigenvalue zero. Then, we note that, since ${{\,\textrm{diam}\,}}(X) \le d( X, \varLambda {\setminus } {\varLambda ^{\textrm{gap}}})^\beta$, the bound in (63) implies that

69 $$\begin{aligned} \bigl \Vert ({{\,\textrm{id}\,}}- \mathbb {E}_{X_n})\mathcal {J}_{H,g}\llbracket A \rrbracket \bigr \Vert \le C \, \Vert A \Vert \, \chi _{b,p}\bigl ( d( X, \varLambda \setminus {\varLambda ^{\textrm{gap}}}) \bigr ) . \end{aligned}$$

We continue by decomposing

70 $$\begin{aligned} \bigl ( \mathcal {J}_{H,g}\llbracket A \rrbracket - \langle \psi , \mathcal {J}_{H,g}\llbracket A \rrbracket \, \psi \rangle \bigr ) |\psi \rangle = |\phi _{\le n} \rangle + |\phi _{> n} \rangle \end{aligned}$$

where we defined

$$\begin{aligned} |\phi _{\le n} \rangle = |\phi _{\le n} \rangle _{X_n} \otimes |\psi \rangle _{X_n^c} := \bigl ( \mathbb {E}_{X_n} \mathcal {J}_{H,g}\llbracket A \rrbracket - \langle \psi , \mathbb {E}_{X_n} \mathcal {J}_{H,g}\llbracket A \rrbracket \, \psi \rangle \bigr ) |\psi \rangle \end{aligned}$$

and $|\phi _{> n} \rangle$, which is defined such that (70) holds, satisfies the bound

$$\begin{aligned} \begin{aligned} \Vert |\phi _{> n} \rangle \Vert&\le \bigl \Vert \bigl ( ({{\,\textrm{id}\,}}- \mathbb {E}_{X_n})\mathcal {J}_{H,g}\llbracket A \rrbracket -\langle \psi , ({{\,\textrm{id}\,}}- \mathbb {E}_{X_n})\mathcal {J}_{H,g}\llbracket A \rrbracket \, \psi \rangle \bigr ) |\psi \rangle \bigr \Vert \\  &\le \bigl \Vert ({{\,\textrm{id}\,}}- \mathbb {E}_{X_n})\mathcal {J}_{H,g}\llbracket A \rrbracket \bigr \Vert \le C \, \Vert A \Vert \, \chi _{b,p}\bigl ( d( X, \varLambda \setminus {\varLambda ^{\textrm{gap}}}) \bigr ) , \end{aligned} \end{aligned}$$

where in the last step we employed (69). Then, similarly to [52, Lemma 15, eq. (B50)], one can compute $\langle \phi _{\le n}, H^2 \phi _{\le n} \rangle$ and use the Paley–Zygmund inequality to show that the norm of $|\phi _{\le n} \rangle$ is essentially bounded by the norm of $|\phi _{> n} \rangle$. That is, in our case, we find

71 $$\begin{aligned} \Vert |\phi _{\le n} \rangle \Vert \le C \, \Vert A \Vert \, \chi _{b,p}\bigl ( d( X, \varLambda \setminus {\varLambda ^{\textrm{gap}}}) \bigr ) , \end{aligned}$$

as always modulo adjusting the constants C,b and p. Hence, defining the Hermitian operator

$$\begin{aligned} \widetilde{A} := \mathbb {E}_{X_n} \mathcal {J}_{H,g} \llbracket A \rrbracket - \bigl ( |\phi _{\le n} \rangle _{X_n} \langle \psi |+ \mathrm {h.c.} \bigr ) , \end{aligned}$$

supported in $X_n$ we easily see that $\widetilde{A} |\psi \rangle = \langle \psi , \mathbb {E}_{X_n} \mathcal {J}_{H,g}\llbracket A \rrbracket \, \psi \rangle |\psi \rangle =: \widetilde{E} \, |\psi \rangle$ and the bound (66) follows from (71). □

Proof of (ix): This is obvious from the definitions (25) and (27).

This concludes the proof of Proposition 3.2.

□

### Local Dynamical Gap for the Examples in Sect. 3.5

In this section, we prove the systems considered in Sect. 3.5 to have a local dynamical gap.

#### Perturbations of the Classical Ising Model with Weak Interactions

In this section, we prove the claim of a local dynamical gap from Sect. 3.5.1, where we considered perturbations of the classical Ising model with weak interactions. First, the (unique) ground state vector of (37) and the associated ground state energy is easily found as

$$\begin{aligned} \psi _* = \otimes _{x \in \varLambda } |\downarrow \rangle \quad \text {satisfying} \quad H_* \, \psi _* = \Bigl ( - |\varLambda |+ \tfrac{1}{2} \sum _{x, y \in \varLambda } \lambda (x-y) \Bigr ) \, \psi _* , \end{aligned}$$

and the associated spectral projection (ground state) is simply given by $\rho _* = P_* = |\psi _* \rangle \langle \psi _* |$. We note that this is a globally gapped eigenstate of $H_*$, since the ground state energy corresponding to $\psi _*$ is separated by a spectral gap $g \ge 2\bigl ( 1 - \Vert \lambda \Vert _1 \bigr ) > 0$ from the first excited state, as can be seen by splitting any of the spins.

For the following argument, it is important to observe that, for any given ${\varLambda ^{\textrm{gap}}}\subset \varLambda$, the ground state projection factorizes, i.e.

72 $$\begin{aligned} \rho _* = \Bigl ( \bigotimes _{x \in {\varLambda ^{\textrm{gap}}}} |\downarrow \rangle \langle \downarrow |\Bigr ) \otimes \Bigl ( \bigotimes _{x \in \varLambda \setminus {\varLambda ^{\textrm{gap}}}} |\downarrow \rangle \langle \downarrow |\Bigr ) =: \rho _*^{\varLambda ^{\textrm{gap}}}\otimes \rho _*^{\varLambda \setminus {\varLambda ^{\textrm{gap}}}} . \end{aligned}$$

Indeed, since $\Vert \lambda \Vert _1 < 1$, every ground state ρ of $H = H_* + J$, where J is a *strictly* $\varLambda {\setminus } {\varLambda ^{\textrm{gap}}}$-localized SLT Hamiltonian, as described in Sect. 3.5.1, also factorizes as

73 $$\begin{aligned} \rho = \rho _*^{\varLambda ^{\textrm{gap}}}\otimes \rho ^{\varLambda \setminus {\varLambda ^{\textrm{gap}}}} . \end{aligned}$$

In order to see this, first note that there exists an eigenbasis^11^ of H for which every eigenvector ψ of H can be written as a linear combination $\sum _j c_j \, e_j \otimes \varphi _j$, where $e_j \in \mathcal {H}_{{\varLambda ^{\textrm{gap}}}}$ are eigenvectors ofto a *common* eigenvalue and $\varphi _j \in \mathcal {H}_{\varLambda {\setminus } {\varLambda ^{\textrm{gap}}}}$. Then, to see (73), it suffices to realize that, for every $x \in {\varLambda ^{\textrm{gap}}}$, starting from the *unique* ground state vector $\psi _*\vert _{{\varLambda ^{\textrm{gap}}}} = \otimes _{x \in {\varLambda ^{\textrm{gap}}}} |\downarrow \rangle$ of $H\big \vert _{{\varLambda ^{\textrm{gap}}}}$, the energy cost for flipping the spin $|\downarrow \rangle$ to $|\uparrow \rangle$ in the first term of (37) is two, whereas the potential gain stemming from the second summand in (37) is bounded by $2\Vert \lambda \Vert _1 <2$, yielding (73). In particular, any *overall* pure ground state of H can be obtained by tensorizing the unique separate ground state vector of $H\vert _{{\varLambda ^{\textrm{gap}}}}$, i.e. $\psi _*\vert _{\varLambda ^{\textrm{gap}}}$, with an appropriate (not necessarily unique) minimizer $\varphi ^{\varLambda {\setminus }{\varLambda ^{\textrm{gap}}}}$ of

$$\begin{aligned} \min _{\begin{array}{c} \varphi \in \mathcal {H}_{\varLambda \setminus {\varLambda ^{\textrm{gap}}}} \\ \Vert \varphi \Vert = 1 \end{array}} \Bigl \langle \psi _*\vert _{{\varLambda ^{\textrm{gap}}}} \otimes \varphi , (H - H\vert _{{\varLambda ^{\textrm{gap}}}}) \, \psi _*\vert _{{\varLambda ^{\textrm{gap}}}} \otimes \varphi \Bigr \rangle , \end{aligned}$$

i.e. by conditioning on the first factor $\psi _*\vert _{{\varLambda ^{\textrm{gap}}}}$. The pure ground state is then obtained as $|\psi _*\vert _{{\varLambda ^{\textrm{gap}}}} \rangle \langle \psi _* \vert _{\varLambda ^{\textrm{gap}}}|\otimes |\varphi ^{\varLambda {\setminus }{\varLambda ^{\textrm{gap}}}} \rangle \langle \varphi ^{\varLambda {\setminus }{\varLambda ^{\textrm{gap}}}} |$.

Therefore, combining (72) and (73), we have the following: For ρ being a *pure state*, i.e. $\rho ^{\varLambda {\setminus }{\varLambda ^{\textrm{gap}}}}$ from (73) can be written as $\rho ^{\varLambda {\setminus }{\varLambda ^{\textrm{gap}}}} = |\varphi ^{\varLambda {\setminus }{\varLambda ^{\textrm{gap}}}} \rangle \langle \varphi ^{\varLambda {\setminus }{\varLambda ^{\textrm{gap}}}} |$, there exists a unitary $U \equiv U^{\varLambda {\setminus }{\varLambda ^{\textrm{gap}}}} \in \mathcal {A}_{\varLambda {\setminus } {\varLambda ^{\textrm{gap}}}}$ such that $|\varphi ^{\varLambda {\setminus }{\varLambda ^{\textrm{gap}}}} \rangle = U \otimes _{x \in \varLambda {\setminus } {\varLambda ^{\textrm{gap}}}} |\downarrow \rangle$. In particular, $\rho = U \rho _* U^*$ and hence we have a norm-preserving ∗-automorphism $\tau \llbracket A \rrbracket := U^*AU$ on $A \in \mathcal {A}$, which intertwines the ground states, i.e. $\langle \,\cdot \,\rangle _{\rho } = \langle \tau \llbracket \,\cdot \,\rrbracket \rangle _{\rho _*}$, and satisfies (32). By means of Proposition 3.2 (vi) (note that, since $\rho _*$ is spectrally gapped, it fulfills the additional assumption of Proposition 3.2 (vi) by means of Proposition 3.1), we thus find that ρ satisfies Assumption $(\textrm{LDG}_{\textrm{main}})$. Finally, for a general (*mixed*) state ρ, we conclude the desired after noticing that Assumption $(\textrm{LDG}_{\textrm{main}})$ is invariant under taking convex combinations.

#### Perturbations of Gapped Frustration Free Product States

In this section, we prove the claim of a local dynamical gap from Sect. 3.5.2, where we considered perturbations of gapped frustration free Hamiltonians with a product ground state.

Similarly to Sect. 5.3.1, one can easily verify that $H_*$ from (39) is globally gapped with its ground state vector being given by $\otimes _{x \in \varLambda }|\psi _{*,x} \rangle$. The same is true for all restrictions $H_*\vert _{\varLambda '}$.

Moreover, for ${\varLambda ^{\textrm{gap}}}\subset \varLambda$ and a fixed exponent β>0, consider $X \subset \varLambda$ satisfying ${{\,\textrm{diam}\,}}(X) \le d( X, \varLambda {\setminus }{\varLambda ^{\textrm{gap}}})^\beta$. Under Assumptions (A1), (A2), and (A3), the authors of [2] have proven the following (see [2, Displayed equation below (13)]): Let $|\psi \rangle$ be a ground state vector of $H = H_* + J$, where J is a *strongly* $\varLambda {\setminus } {\varLambda ^{\textrm{gap}}}$-localized SLT Hamiltonian, as described in Sect. 3.5.2, and $P_{*,\tilde{X}}$ denote the projection onto the ground state vector $|\psi _*\vert _{\tilde{X}} \rangle$ of $H_*\vert _{\tilde{X}}$, where we introduced the fattened set $\tilde{X}:= X_{d( X, \varLambda \setminus {\varLambda ^{\textrm{gap}}})^\beta /2}$. Then it holds that

74 $$\begin{aligned} \Vert \rho - \tilde{\rho } \Vert _{\textrm{tr}} \le C \, \exp \bigl ( - \bigl ( d( X, \varLambda \setminus {\varLambda ^{\textrm{gap}}}) \bigr )^q \bigr ) \end{aligned}$$

for some C,q>0 and $\Vert \,\cdot \,\Vert _{\textrm{tr}}$ being the trace norm. Here, $\rho \equiv P$ and $\tilde{\rho } \equiv \tilde{P}$ denote the orthogonal projections on $|\psi \rangle$ and $P_{*,\tilde{X}} |\psi \rangle$, respectively, i.e. they are *pure states*.

Due to the product structure of the ground state vector of $H_*$ and its restrictions, we easily see that $\tilde{\rho }$ can be written as

$$\begin{aligned} \tilde{\rho } = |\psi _*\vert _{\tilde{X}} \rangle \langle \psi _*\vert _{\tilde{X}} |\otimes \tilde{\rho }^{\varLambda \setminus \tilde{X}} \end{aligned}$$

for some state $\tilde{\rho }^{\varLambda {\setminus } \tilde{X}}$ on $\varLambda {\setminus } \tilde{X}$. This means that, analogously to Sect. 5.3.1, $\langle \,\cdot \,\rangle _{\rho _*}$ and $\langle \,\cdot \,\rangle _{\tilde{\rho }}$ can be intertwined by a norm preserving ∗-automorphism τ satisfying (32). In particular, by means of Proposition 3.2 (vi) (note that, since $\rho _*$ is spectrally gapped, it fulfills the additional assumption of Proposition 3.2 (vi) by means of Proposition 3.1), we thus find that $\tilde{\rho }$ satisfies Assumption $(\textrm{LDG}_{\textrm{main}})$—but only for observables supported in $X \subset \varLambda$ with ${{\,\textrm{diam}\,}}(X) \le d( X, \varLambda {\setminus }{\varLambda ^{\textrm{gap}}})^\beta /10$, say, and without $d( Y, \varLambda {\setminus } {\varLambda ^{\textrm{gap}}})$ in the argument of $\chi _{b,p}$; that is, Assumption $(\textrm{LDG}_{\textrm{weak}})$. (We point out that $\tilde{\rho }$ being an equilibrium state is *not* required for the argument in Proposition 3.2 (vi) to work.) This implies, by means of Proposition 3.2 (ii) and (v) (modified to the setting of $(\textrm{LDG}_{\textrm{weak}})$) and (74), that ρ satisfies Assumption $(\textrm{LDG}_{\textrm{weak}})$. Finally, for a general (*mixed*) state ρ, we conclude the desired by taking convex combinations (as at the end of the argument in Sect. 5.3.1).

## Construction of the NEASS: Proofs for Sect. 4

The fundamental conceptual idea behind the proof of Proposition 4.4 is a perturbative scheme, which was called *space-time adiabatic perturbation theory* in [46, 47]. Before going into this expansion in Sect. 6.2, we show that the weakened local dynamical gap condition $(\textrm{LDG}_{\textrm{weak}})$ from Sect. 3.1 carries over to SLT operators. The main technical input for carrying out the space-time adiabatic perturbation scheme, is to show that all the operations involved in the expansion preserve localization of SLT operators as required for Lemma 6.1 to apply. This is the content of several auxiliary technical results in Appendix A.

### Local Dynamical Gap Condition for SLT Operators

Throughout the proof, we will work with the weakened version of the local dynamical gap condition $(\textrm{LDG}_{\textrm{weak}})$ from Sect. 3.1. As mentioned above, we start with the following basic lemma which will heavily be used in our proof. It says that the local dynamical gap condition naturally carries over to SLT operators.

#### Lemma 6.1

Let b>0, p∈(0,1) and $H_0$ be a $\chi _{b, p}$-SLT operator. Assume that the equilibrium state $\rho _0$ of $H_0$ satisfies the weakened local gap condition, Assumption $(\textrm{LDG}_{\textrm{weak}})$ above, with gap size at least g>0 and with respect to $C_{\textrm{gap}}$, b, p, β and ℓ.

Then, there exists a constant C, such that for any (b,p,Ω)-localized SLT operator A and observable $B \in \mathcal {A}_Y$, we have that

75 $$\begin{aligned}&\Bigl |\bigl \langle \bigl [ \mathcal {L}_{H_0} \circ \mathcal {I}_{H_0,g}\llbracket A \rrbracket - A , B \bigr ] \bigr \rangle _{\rho _0} \Bigr |\nonumber \\&\quad \le C \, \bigl ( 1+{{\,\textrm{diam}\,}}(Y) \bigr )^{\ell + d} \, \Vert B \Vert \, \Vert \varPhi _A \Vert _{b,p; \varOmega } \, \chi _{b/2,p \min \{\beta , 1\}}\bigl ( d( \varOmega , \varLambda \setminus {\varLambda ^{\textrm{gap}}}) \bigr ) . \end{aligned}$$

The proof of Lemma 6.1 is presented in Appendix A.5. The principal idea is to write $A = \sum _{Z \subset \varLambda } \varPhi _A(Z)$ and then estimate only the contribution of ‘small’ Z, i.e. those with ${{\,\textrm{diam}\,}}(Z) \le d( Z, \varLambda {\setminus } {\varLambda ^{\textrm{gap}}})^\beta$, by (27). Large Z’s, i.e. those with ${{\,\textrm{diam}\,}}(Z) > d( Z, \varLambda {\setminus } {\varLambda ^{\textrm{gap}}})^\beta$, are treated using Lieb-Robinson bounds and the smallness of $\Vert \varPhi _A(Z) \Vert$ (by definition (18)).

### The Adiabatic Perturbation Scheme

For the proof of Proposition 4.4 we follow the strategy from [49]. However, since we only have a local gap, the lower order terms do not vanish exactly, but can be bounded using Assumption $(\textrm{LDG}_{\textrm{weak}})$.

The statements in Propositions 4.3–4.5 will be deduced from a time-dependent NEASS, which is part of the next Theorem. In contrast to the previous works, it will not include a time-dependent unperturbed Hamiltonian $H_0(t)$, because there is no spectral flow available. That means, we construct a time-dependent NEASS $\varPi _n^\varepsilon (t)$ specifically for the switching Hamiltonian given in (42). For times t≥0 it will turn out to be time-independent.

In order to formulate the result, we introduce time-dependent interactions

$$\begin{aligned} \varPhi :I \times \{X \subset \varLambda \} \rightarrow \mathcal {A}, \quad (t,X) \mapsto \varPhi (t,X) = \varPhi (t,X)^* \in \mathcal {A}_X \end{aligned}$$

for $I \subset \mathbb {R}$. We will assume that $t \mapsto \varPhi (t,X)$ is smooth for every $X \subset \varLambda$, and we denote the term-wise time derivatives by $\varPhi ^{(k)}$, i.e. $\varPhi ^{(k)}(t,X) = \frac{\textrm{d}^k}{\textrm{d}t^k} \varPhi (t,X)$ for every $X \subset \varLambda$. Moreover, we identify $\varPhi (t,X) = \bigl ( \varPhi (t) \bigr )(X)$, such that for every fixed t∈I, Φ(t) can be viewed as a time-independent interaction. The notion of SLT operators naturally translates to the time-dependent setting.

#### Theorem 6.2

(Time-dependent NEASS) Let $d\in \mathbb {N}$, $C_{\textrm{vol}}>0$, b>0, p∈(0,1], $C_{\textrm{int}}>0$, g>0, $C_\textrm{gap}>0$, β>0, $\ell \in \mathbb {N} _0$, $q \in (0, p \min \{1, \beta \})$, $n\in \mathbb {N}$. Then there exists a constant $C_{n}> 0$ such that the following holds:

Let $\varLambda \in \mathcal {G}(d,C_{\textrm{vol}})$ (recall (13)), ${\varLambda ^{\textrm{gap}}}, {\varLambda ^{\textrm{pert}}}\subset \varLambda$, $I\subset \mathbb {R}$ be an interval and $H_0$ and V(t) be SLT-operators, with corresponding interactions that satisfy $\Vert \varPhi _{H_0} \Vert _{b,p}<C_{\textrm{int}}$ and $\sup _{t\in I} \Vert \varPhi ^{(k)}_{V}(t) \Vert _{b,p;{\varLambda ^{\textrm{pert}}}}<C_{\textrm{int}}$ for all k≤n, respectively. Moreover, assume that the equilibrium state $\rho _0$ of $H_0$ is locally dynamically gapped in ${\varLambda ^{\textrm{gap}}}$ of size at least g>0 and with respect to $C_\textrm{gap}$, b, p, β and ℓ according to Assumption $(\textrm{LDG}_{\textrm{weak}})$. And let

76 $$\begin{aligned} H_\varepsilon (t) := H_0 + \varepsilon \, V(t) \end{aligned}$$

be the perturbed Hamiltonian.

Then, there exists a sequence $$(A_\mu ^{\delta })_{\mu \in \mathbb {N} }$$ of polynomials in $$\delta \in \mathbb {R} $$ with maximal degree μ-1 and time-dependent SLT operators as coefficients, which are $$(1,p',\varLambda ^{\textrm{pert}})$$-localized for any $p^{\prime }<p$. The $$A_\mu ^{\delta }$$ are such that the state

77 $$\begin{aligned} \varPi _n^{\varepsilon ,\eta }(t) := \textrm{e}^{\textrm{i}S_n^{\varepsilon ,\eta }(t)}\,\rho _0\,\textrm{e}^{-\textrm{i}S_n^{\varepsilon ,\eta }(t)} \quad \text {with}\quad S_n^{\varepsilon ,\eta }(t) := \sum _{\mu = 1}^n \varepsilon ^\mu A_\mu ^{\eta /\varepsilon }(t) , \end{aligned}$$

is almost-stationary for the dynamics generated by $H_\varepsilon (t)$ in the following sense: Let $t_0\in \mathbb {R}$ and let $\rho ^{\varepsilon ,\eta }_{t_0}(t)$ be the solution of the time-dependent adiabatic Schrödinger equation

78 $$\begin{aligned} \textrm{i}\eta \frac{\textrm{d}}{\textrm{d}t} \rho ^{\varepsilon ,\eta }_{t_0}(t) = \bigl [ H_\varepsilon (t) , \rho ^{\varepsilon ,\eta }_{t_0}(t) \bigr ] \quad \text {with}\quad \rho ^{\varepsilon ,\eta }_{t_0}(t_0) = \varPi _n^{\varepsilon ,\eta }(t_0) \end{aligned}$$

with adiabatic parameter $\eta \in ( 0,1 ]$. For all $B\in \mathcal {A}_Y$ with $Y\subset \varLambda$ and $t \in \mathbb {R}$, it holds that

79 $$\begin{aligned} \bigl |\langle B \rangle _{\rho ^{\varepsilon ,\eta }_{t_0}(t)} - \langle B \rangle _{\varPi _n^{\varepsilon ,\eta }(t)} \bigr |\le &   C_{n} \, \Vert B \Vert \, \bigl ( 1+{{\,\textrm{diam}\,}}(Y) \bigr )^{3d+\ell } \, \biggl ( 1+\frac{\eta ^{n}}{\varepsilon ^{n}} \biggr ) \, \varepsilon ^{n+1} \nonumber \\\times &   \, \frac{|t-t_0 |}{\eta } \, \biggl ( 1 + \frac{v\,|t-t_0 |}{\eta } \biggr )^{(\ell +2d)/p} \nonumber \\\times &   \Bigl ( 1 + \textrm{e}^{-d( {\varLambda ^{\textrm{pert}}},\varLambda \setminus {\varLambda ^{\textrm{gap}}})^q - (n+1) \log (\varepsilon )} \Bigr ) . \end{aligned}$$

Moreover, the operators $A_\mu (t)$ at time t depend only on V and its first μ derivatives at time t.

Before we prove Theorem 6.2, let us deduce the results from Sect. 4, which will follow by taking β=1 in Theorem 6.2.

#### Proof of Proposition 4.3

We choose V(t)=V, which implies that also all $A^{\eta /\varepsilon }_\mu (t) = A^{\eta /\varepsilon }_\mu$ are time-independent (i.e. the time-dependent interaction is constant). Moreover, since there is no adiabatic timescale in (47), we choose η=1. To obtain the correct scaling, we inspect the proof of Theorem 6.2: In equation (82), we expand in powers of ε and $R_j^{\varepsilon ,\eta }$ are polynomials in $\eta /\varepsilon$. Here, since $\dot{S}_n^{\varepsilon ,\eta } = 0$, these polynomials are just constants, $A^{\eta /\varepsilon }_\mu = A_\mu$, and there is no η in (82) or any of the later expressions. Hence, also the norm estimates $\Vert \varPhi _{\tilde{R}_j^{\varepsilon ,\eta }}(s) \Vert < C \, (1+\eta ^{j-1}/\varepsilon ^{j-1})$ used in the end of the proof simplify to $\Vert \varPhi _{\tilde{R}_j^{\varepsilon }} \Vert < C$ uniformly in ε. Hence, $(1+\eta ^{n}/\varepsilon ^{n})$ is replaced by 1 in (79). All other η in (79) come from the adiabatic timescale and are thus replaced by 1. □

#### Proof of Proposition 4.4

We choose V(t)=f(t)V and since V(t) is constant for t≥0, we obtain $\varPi _n^{\varepsilon ,\eta }(t) = \varPi _n^\varepsilon$ for all t≥0. To compare with the solution of (43), we choose $t_0=-1$ such that $\varPi _n^{\varepsilon ,\eta }(t_0) = \rho _0$. Then $|t-t_0 |=1+t$ and (79) gives the statement. □

#### Proof of Proposition 4.5

To prove the asymptotic expansion, we first expand (46) and obtain

$$\begin{aligned} {{\,\textrm{tr}\,}}\bigl ( \varPi _n^\varepsilon \, B \bigr ) = {{\,\textrm{tr}\,}}\bigl ( \rho _0 \, \textrm{e}^{\mathcal {L}_{S_n^\varepsilon }}\llbracket B \rrbracket \bigr ) = \sum _{k=0}^m \frac{1}{k!} \, \bigl \langle \mathcal {L}^k_{S_n^\varepsilon }\llbracket B \rrbracket \bigr \rangle _{\rho _0} + \frac{1}{(m+1)!} \, {{\,\textrm{tr}\,}}\bigl ( \rho _0 \, \textrm{e}^{ \theta \, \mathcal {L}_{S_n^{\varepsilon }}}\circ \mathcal {L}^{m+1}_{S_n^{\varepsilon }}\llbracket B \rrbracket \bigr ) \end{aligned}$$

for some $\theta \in [ 0,1 ]$. Since the $A_\mu$ and thus also the $S_n^\varepsilon$ are ${\varLambda ^{\textrm{pert}}}$-localized, we use Lemma A.4 to bound the remainder by

$$\begin{aligned} \frac{1}{(m+1)!} \, \bigl \Vert \mathcal {L}^{m+1}_{S_n^\varepsilon }\llbracket B \rrbracket \bigr \Vert \le C \, \Vert B \Vert \, |Y |^{m+1} \, \chi _{1,p'}\bigl ( d( Y,{\varLambda ^{\textrm{pert}}}) \bigr ) \, \Vert S_n^\varepsilon \Vert _{1,p';{\varLambda ^{\textrm{pert}}}}^{m+1} , \end{aligned}$$

where $\Vert S_n^\varepsilon \Vert _{1,p';{\varLambda ^{\textrm{pert}}}} \le \varepsilon \, \tilde{C}$. It is clear from the proof of Theorem 6.2, that $\tilde{C}$ depends only on $n, d,C_{\textrm{vol}}, b, p, p', C_{\textrm{int}}$, and g. We now expand $S_n^\varepsilon$ in the first term and group the terms according to the powers in ε. The zero order term clearly is $\langle B \rangle _{\rho _0}$. In first order, we obtain $\varepsilon \, \langle \mathcal {L}_{A_1}\llbracket B \rrbracket \rangle _{\rho _0} = - \varepsilon \,\textrm{i}\, \left\langle \bigl [ \mathcal {I}_{H_0,g}\llbracket V \rrbracket , B \bigr ] \right\rangle _{\rho _0}$ as can be read off from (85). All $\mathcal {K}_j$ for j≤m are constructed in this way. In the end, some higher order terms are left. They all come with multi-commutators of ${\varLambda ^{\textrm{pert}}}$-localized $A_\mu$ with B and can be bounded as the remainder above using Lemma A.4. □

We now prove the time-dependent NEASS from Theorem 6.2.

#### Proof of Theorem 6.2

For the proof we first assume the more general form $S_n^{\varepsilon ,\eta }(t):= \sum _{\mu = 1}^n \varepsilon ^\mu A_\mu ^{\varepsilon , \eta }(t)$ of (77) (it will become clear from the proof, that $A_\mu ^{\varepsilon , \eta }(t)$ is actually a polynomial in $\eta /\varepsilon$) and then iteratively choose $A_\mu ^{\varepsilon , \eta }(t)$ such that the statement holds.

Therefore, let $U_{t,t_0}^{\varepsilon ,\eta }$ be the solution of

80 $$\begin{aligned} \textrm{i}\, \eta \, \frac{\textrm{d}}{\textrm{d}t} \, U_{t,t_0}^{\varepsilon ,\eta } = H_\varepsilon (t) \, U_{t,t_0}^{\varepsilon ,\eta } \quad \quad \text {with}\quad \quad U_{t_0,t_0}^{\varepsilon ,\eta } = \textbf{1}\quad \text {for all}\quad t,t_0 \in I , \end{aligned}$$

with $H_\varepsilon$ given in (42). Then, $\rho ^{\varepsilon ,\eta }_{t_0}(t):= U^{\varepsilon ,\eta }_{t,t_0} \, \varPi _n^{\varepsilon ,\eta }(t_0) \, U^{\varepsilon ,\eta }_{t_0,t}$ is the solution of (78). To obtain (79) we use the fundamental theorem of calculus, and get

81 $$\begin{aligned} \langle B \rangle _{\rho ^{\varepsilon ,\eta }_{t_0}(t)} - \langle B \rangle _{\varPi _n^{\varepsilon ,\eta }(t)} = - \int _{t_0}^t \textrm{d}s \, \frac{\textrm{d}}{\textrm{d}s} \, \Bigl \langle \textrm{e}^{-\textrm{i}S_n^{\varepsilon ,\eta }(s)}\,U^{\varepsilon ,\eta }_{s,t}\,B\,U^{\varepsilon ,\eta }_{t,s}\,\textrm{e}^{\textrm{i}S_n^{\varepsilon ,\eta }(s)} \Bigr \rangle _{\rho _0} . \end{aligned}$$

By product rule and Duhamel’s formula, the derivative evaluates as

$$\begin{aligned} \frac{\textrm{d}}{\textrm{d}s} \, \textrm{e}^{-\textrm{i}S_n^{\varepsilon ,\eta }(s)}\,U^{\varepsilon ,\eta }_{s,t}\,B\,U^{\varepsilon ,\eta }_{t,s}\,\textrm{e}^{\textrm{i}S_n^{\varepsilon ,\eta }(s)} = -\frac{\textrm{i}}{\eta } \, \Bigl [ Q^{\varepsilon ,\eta }_n(s) , \textrm{e}^{-\textrm{i}S_n^{\varepsilon ,\eta }(s)}\,U^{\varepsilon ,\eta }_{s,t}\,B\,U^{\varepsilon ,\eta }_{t,s}\,\textrm{e}^{\textrm{i}S_n^{\varepsilon ,\eta }(s)} \Bigr ] , \end{aligned}$$

where

82 $$\begin{aligned} Q^{\varepsilon ,\eta }_n(s)&= \eta \, \int _0^1 \textrm{d}\lambda \, \textrm{e}^{-\textrm{i}\lambda S^{\varepsilon ,\eta }_n(s)}\,\dot{S}^{\varepsilon ,\eta }_n(s)\,\textrm{e}^{\textrm{i}\lambda S^{\varepsilon ,\eta }_n(s)} + \textrm{e}^{-\textrm{i}S_n^{\varepsilon ,\eta }(s)}\,\bigl ( H_0 + \varepsilon \, V(s) \bigr )\,\textrm{e}^{\textrm{i}S_n^{\varepsilon ,\eta }(s)} \nonumber \\&= \eta \, \int _0^1 \textrm{d}\lambda \, \textrm{e}^{\lambda \mathcal {L}_{S^{\varepsilon ,\eta }_n(s)}} \llbracket \dot{S}^{\varepsilon ,\eta }_n(s) \rrbracket + \textrm{e}^{\mathcal {L}_{S_n^{\varepsilon ,\eta }(s)}}\llbracket H_0 + \varepsilon \, V(s) \rrbracket \nonumber \\&= H_0 + \sum _{j=1}^n \varepsilon ^j \, R_j^{\varepsilon ,\eta }(s) + \varepsilon ^{n+1} \, R_{n+1}^{\varepsilon ,\eta }(s). \end{aligned}$$

In the last line we expanded in powers of ε and η such that $R_j^{\varepsilon ,\eta }(s)$ are polynomials in $\eta /\varepsilon$ of degree j-1 with ε- and η-independent SLT operators as coefficients. In this way, the joint power of ε and η in front of the SLT operators collected in $R_j^{\varepsilon ,\eta }$ is j and there is at least one ε. By Taylor formula with mean-value form of the remainder, there exists $\theta \in [ 0,1 ]$ such that

83 $$\begin{aligned} \textrm{e}^{\mathcal {L}_{S^{\varepsilon ,\eta }_n(s)}} \llbracket H_0 + \varepsilon \, V(s) \rrbracket= &   \sum _{k=0}^n \frac{1}{k!} \, \mathcal {L}_{S^{\varepsilon ,\eta }_n(s)}^k \llbracket H_0 + \varepsilon \, V(s) \rrbracket \nonumber \\  &   + \frac{1}{(n+1)!} \, \textrm{e}^{\theta \mathcal {L}_{S^{\varepsilon ,\eta }_n(s)}} \circ \mathcal {L}_{S^{\varepsilon ,\eta }_n(s)}^{n+1} \llbracket H_0 + \varepsilon \, V(s) \rrbracket . \end{aligned}$$

Similarly, for the first term in (82) we expand the integrand using the integral form of the remainder and obtain

84 $$\begin{aligned} \nonumber \hspace{2em}&\hspace{-2em} \eta \, \int _0^1 \textrm{d}\lambda \, \textrm{e}^{\lambda \mathcal {L}_{S^{\varepsilon ,\eta }_n(s)}} \llbracket \dot{S}^{\varepsilon ,\eta }_n(s) \rrbracket \\  &= \nonumber \begin{aligned}&\eta \, \sum _{k=0}^{n-2} \frac{\int _0^1 \textrm{d}\lambda \, \lambda ^k }{k!} \, \mathcal {L}_{S^{\varepsilon ,\eta }_n(s)}^k \llbracket \dot{S}_n^{\varepsilon ,\eta }(s) \rrbracket \\  &+ \eta \, \int _0^1 \textrm{d}\lambda \, \int _0^\lambda \textrm{d}\mu \, \frac{(\lambda -\mu )^{n-2}}{(n-2)!} \, \textrm{e}^{\mu \mathcal {L}_{S^{\varepsilon ,\eta }_n(s)}} \circ \mathcal {L}_{S^{\varepsilon ,\eta }_n(s)}^{n-1} \llbracket \dot{S}^{\varepsilon ,\eta }_n(s) \rrbracket , \end{aligned} \\  &= \begin{aligned}&\eta \, \sum _{k=0}^{n-2} \frac{1}{(k+1)!} \, \mathcal {L}_{S^{\varepsilon ,\eta }_n(s)}^k \llbracket \dot{S}_n^{\varepsilon ,\eta }(s) \rrbracket \\  &+ \eta \, \int _0^1 \textrm{d}\mu \, \frac{(1-\mu )^{n-1}}{(n-1)!} \, \textrm{e}^{\mu \mathcal {L}_{S^{\varepsilon ,\eta }_n(s)}} \circ \mathcal {L}_{S^{\varepsilon ,\eta }_n(s)}^{n-1} \llbracket \dot{S}^{\varepsilon ,\eta }_n(s) \rrbracket . \end{aligned} \end{aligned}$$

From this expansion and (77), we can read off

85 $$\begin{aligned} R_1^{\varepsilon ,\eta }(s)&= - \mathcal {L}_{H_0}\llbracket A_1^{\varepsilon ,\eta }(s) \rrbracket + V(s) , \end{aligned}$$

86 $$\begin{aligned} R_2^{\varepsilon ,\eta }(s)&= - \mathcal {L}_{H_0}\llbracket A_2^{\varepsilon ,\eta }(s) \rrbracket + \tfrac{1}{2} \, \mathcal {L}^2_{A_1^{\varepsilon ,\eta }(s)}\llbracket H_0 \rrbracket + \mathcal {L}_{A_1^{\varepsilon ,\eta }(s)}\llbracket V(s) \rrbracket + \tfrac{\eta }{\varepsilon } \dot{A}_1^{\varepsilon ,\eta }(s) , \end{aligned}$$

and more generally

87 $$\begin{aligned} R_j^{\varepsilon ,\eta }(s)&= - \mathcal {L}_{H_0}\llbracket A_j^{\varepsilon ,\eta }(s) \rrbracket + \tilde{R}_j^{\varepsilon ,\eta }(s) , \end{aligned}$$

where the $\tilde{R}_j^{\varepsilon ,\eta }(s)$ are sums of iterated commutators of the operators $A_i^{\varepsilon ,\eta }(s)$ and $\dot{A}_i^{\varepsilon ,\eta }(s)$ for i<j≤n and V(s). We can now iteratively choose

$$\begin{aligned} A_j^{\varepsilon ,\eta }(s) = \mathcal {I}_{H_0,g}\llbracket \tilde{R}_j^{\varepsilon ,\eta }(s) \rrbracket . \end{aligned}$$

Clearly, for all $p^{\prime }<p$, it holds that $$\tilde{R}_1^{\varepsilon ,\eta }(s) = V(s)$$ is a $$(b,p',{\varLambda ^{\textrm{pert}}})$$-localized SLT operator. Hence, by Lemma A.11, $$A_1^{\varepsilon ,\eta }(s)$$ is $$(b',p',{\varLambda ^{\textrm{pert}}})$$-localized for any $b^{\prime }<b$. This step only works for $p^{\prime }<p$, because Lemma A.11 requires a slightly better localization of the Hamiltonian $$H_0$$ compared to the argument of the inverse Liouvillian. Finally, by Lemma A.5 also $$\mathcal {L}_{H_0}\llbracket A_1^{\varepsilon ,\eta }(s) \rrbracket $$ and thus $$R_1^{\varepsilon ,\eta }(s)$$ are $$(b',p',{\varLambda ^{\textrm{pert}}})$$-localized SLT operator for any slightly smaller $b^{\prime }$. The same arguments hold for the higher orders $$R_j^{\varepsilon ,\eta }(s)$$ with j≤n as well, at each step lowering $b^{\prime }$. We point out that clearly the smallest $b^{\prime }$ can be chosen independently of n by choosing all intermediate $b^{\prime }$ in an n-dependent equidistant way.

With this expansion it is also immediate, that the $A^{\varepsilon ,\eta }_\mu (s)$ only depend on V and its derivatives at time s. Moreover, they are polynomials in $\eta /\varepsilon$ of degree j-1 with ε- and η-independent, time-dependent $(b',p',{\varLambda ^{\textrm{pert}}})$-localized SLT operators as coefficients. Thus, we denote them $A^{\eta /\varepsilon }_\mu (s) = A^{\varepsilon ,\eta }_\mu (s)$ in the statement.

Putting everything back together and denoting $\tau _{t,s} \llbracket B \rrbracket := U^{\varepsilon ,\eta }_{s,t}\,B\,U^{\varepsilon ,\eta }_{t,s}$, we find

88 $$\begin{aligned} \hspace{2em}&\hspace{-2em} \Bigl |\langle B \rangle _{\rho ^{\varepsilon ,\eta }_{t_0}(t)} - \langle B \rangle _{\varPi _n^{\varepsilon ,\eta }(t)} \Bigr |\\  &\le \nonumber \frac{|t-t_0 |}{\eta } \sup _{s\in [ t_0,t ]} \left( \begin{aligned}&\, \sum _{j=1}^{n} \varepsilon ^j \, \biggl |\Bigl \langle \Bigl [ \mathcal {L}_{H_0} \circ \mathcal {I}_{H_0,g}\llbracket \tilde{R}_j^{\varepsilon ,\eta }(s) \rrbracket - \tilde{R}_j^{\varepsilon ,\eta }(s) , \textrm{e}^{\mathcal {L}_{S_n^{\varepsilon ,\eta }(s)}} \circ \tau _{t,s}\llbracket B \rrbracket \Bigr ] \Bigr \rangle _{\rho _0} \biggr |\\  &+ \varepsilon ^{n+1} \, \biggl |\Bigl \langle \Bigl [ R_{n+1}^{\varepsilon ,\eta }(s) , \textrm{e}^{\mathcal {L}_{S_n^{\varepsilon ,\eta }(s)}} \circ \tau _{t,s}\llbracket B \rrbracket \Bigr ] \Bigr \rangle _{\rho _0} \biggr |. \end{aligned} \right) \end{aligned}$$

The remainder $R_{n+1}^{\varepsilon ,\eta }(s)$ collects all the remaining terms which are (a) the higher order terms from the first lines and (b) the remainder terms from the second lines of (83) and (84). The former are local by the previous arguments. The latter additionally include an evolution by the local operator $S_n^{\varepsilon ,\eta }(s)$ and are local by Lemma A.8.

To apply the local gap assumption in the form given in Lemma 6.1 to the lower order terms, we need to decompose the second entry of the commutator into strictly local operators. Therefore, we use the same decomposition as in the proof of Lemma A.7

$$\begin{aligned} \tau _{t,s}\llbracket B \rrbracket =\sum _{k=0}^\infty \varDelta _k , \end{aligned}$$

where

$$\begin{aligned} \begin{aligned} \varDelta _0&\in \mathcal {A}_{Y_{\delta }} \subset \mathcal {A}_{Y_{\delta ^{1/p}}},&\Vert \varDelta _0 \Vert&\le \Vert B \Vert , \\ \text {and for } k\in \mathbb {N} \qquad \varDelta _k&\in \mathcal {A}_{Y_{(\delta +k)^{1/p}}},&\Vert \varDelta _k \Vert&\le C_\textrm{LR}\, \textrm{e}^{b} \, \Vert B \Vert \, |Y |\, \textrm{e}^{-b k} . \end{aligned} \end{aligned}$$

For better readability we abbreviate $\delta =1+v \, |t-s |/\eta$ here. The extra 1/η is due to the scaling in (80). For the outer automorphism, we can use the decomposition from the proof of Lemma A.8, just replace $O=\varDelta _k$ there, to obtain, for each k, the decomposition

$$\begin{aligned} \textrm{e}^{\mathcal {L}_{S_n^{\varepsilon ,\eta }(s)}}\llbracket \varDelta _k \rrbracket = \sum _{l=0}^\infty \varDelta _{k,l} \end{aligned}$$

with

$$\begin{aligned} \begin{aligned} \varDelta _{k,0}&\in \mathcal {A}_{Y_{(\delta +k)^{1/p}}},&\Vert \varDelta _{k,0} \Vert&\le \Vert \varDelta _k \Vert , \quad \text {and} \\ \varDelta _{k,l}&\in \mathcal {A}_{Y_{l+(\delta +k)^{1/p}}},&\Vert \varDelta _{k,l} \Vert&\le C_\textrm{LR}\, \Vert \varDelta _k \Vert \, \bigl |Y_{(\delta +k)^{1/p}} \bigr |\, \bigl ( \textrm{e}^{c \, \Vert \varPhi _S \Vert _{d,q}} - 1 \bigr ) \, \chi _{d',j}(l) \end{aligned} \end{aligned}$$

for $l \in \mathbb {N}$. Thus, in total we have

$$\begin{aligned} \textrm{e}^{\mathcal {L}_{S_n^{\varepsilon ,\eta }(s)}} \circ \tau _{t,s}\llbracket B \rrbracket = \sum _{k,l=0}^\infty \varDelta _{k,l} , \end{aligned}$$

where the sum is actually finite on finite lattices as discussed in Lemmata A.7 and A.8. Then we use the triangle inequality and Lemma 6.1 to apply (75) and bound

$$\begin{aligned} \hspace{2em}&\hspace{-2em} \Bigl |\Bigl \langle \Bigl [ \mathcal {L}_{H_0} \circ \mathcal {I}_{H_0,g}\llbracket \tilde{R}_j^{\varepsilon ,\eta }(s) \rrbracket - \tilde{R}_j^{\varepsilon ,\eta }(s) , \textrm{e}^{\mathcal {L}_{S_n^{\varepsilon ,\eta }(s)}} \circ \tau _{t,s}\llbracket B \rrbracket \Bigr ] \Bigr \rangle _{\rho _0} \Bigr |\\  &\le C \, \Vert \varPhi _{\tilde{R}_j^{\varepsilon ,\eta }}(s) \Vert _{b',p'; {\varLambda ^{\textrm{pert}}}} \, \sum _{k,l=0}^\infty \bigl ( 1+{{\,\textrm{diam}\,}}(Y_{l+(\delta +k)^{1/p}}) \bigr )^{\ell + d} \, \Vert \varDelta _{k,l} \Vert \, \chi _{b'/2,q'}\\  &\quad \bigl ( d( {\varLambda ^{\textrm{pert}}}, \varLambda \setminus {\varLambda ^{\textrm{gap}}}) \bigr ) \\  &\le \begin{aligned}&C \, \Vert \varPhi _{\tilde{R}_j^{\varepsilon ,\eta }}(s) \Vert _{b',p'; {\varLambda ^{\textrm{pert}}}} \, \sum _{k=0}^\infty \bigl ( 1+{{\,\textrm{diam}\,}}(Y_{(\delta +k)^{1/p}}) \bigr )^{\ell + 2d} \, \Vert \varDelta _{k} \Vert \\  &\times \biggl ( 1 + \bigl ( \textrm{e}^{c \, \Vert \varPhi _S \Vert _{d,q}} - 1 \bigr ) \sum _{l=1}^\infty l^{\ell +d} \, \chi _{d',j}(l) \biggr ) \, \chi _{b'/2,q'}\bigl ( d( {\varLambda ^{\textrm{pert}}}, \varLambda \setminus {\varLambda ^{\textrm{gap}}}) \bigr ) \end{aligned} \\  &\le \begin{aligned}&C \, \Vert \varPhi _{\tilde{R}_j^{\varepsilon ,\eta }}(s) \Vert _{b',p'; {\varLambda ^{\textrm{pert}}}} \, \textrm{e}^{c \, \Vert \varPhi _S \Vert _{d,q}} \, \bigl ( 1+{{\,\textrm{diam}\,}}(Y) \bigr )^{\ell + 3d} \, \Vert B \Vert \, \delta ^{(\ell +2d)/p} \\  &\times \sum _{k=0}^\infty \, (1+k)^{(\ell +2d)/p} \, \textrm{e}^{-bk} \, \chi _{b'/2,q'}\bigl ( d( {\varLambda ^{\textrm{pert}}}, \varLambda \setminus {\varLambda ^{\textrm{gap}}}) \bigr ) \end{aligned} \\  &\le C \, \Vert \varPhi _{\tilde{R}_j^{\varepsilon ,\eta }}(s) \Vert _{b',p'; {\varLambda ^{\textrm{pert}}}} \, \textrm{e}^{c \, \Vert \varPhi _S \Vert _{d,q}} \, \bigl ( 1+{{\,\textrm{diam}\,}}(Y) \bigr )^{\ell + 3d} \, \Vert B \Vert \, \delta ^{(\ell +2d)/p} \, \chi _{b'/2,q'}\\  &\quad \bigl ( d( {\varLambda ^{\textrm{pert}}}, \varLambda \setminus {\varLambda ^{\textrm{gap}}}) \bigr ) , \end{aligned}$$

where we abbreviated $$q':= p' \, \min \{ \beta ,1 \}$$ (according to Lemma 6.1) and $b^{\prime }$ is the smallest of the $b^{\prime }$ such that all $$\varPhi _{\tilde{R}_j^{\varepsilon ,\eta }}(s)$$ are $$(b',p',{\varLambda ^{\textrm{pert}}})$$-localized. Finally, for any $q<q^{\prime }$ we bound

89 $$\begin{aligned} \chi _{b'/2,q'}\bigl ( d( {\varLambda ^{\textrm{pert}}}, \varLambda \setminus {\varLambda ^{\textrm{gap}}}) \bigr ) \le C \, \chi _{1,q}\bigl ( d( {\varLambda ^{\textrm{pert}}}, \varLambda \setminus {\varLambda ^{\textrm{gap}}}) \bigr ) \end{aligned}$$

for some constant C>0 depending only on $b^{\prime }$, $q^{\prime }$, and q. Since $p^{\prime }<p$ was arbitrary, this q can be chosen arbitrarily in the interval $$(0, p \min \{\beta , 1\})$$.

With the same local decomposition argument as above and using Lemma A.4 we can also bound the remainder

$$\begin{aligned} \hspace{2em}&\hspace{-2em} \biggl |\Bigl \langle \Bigl [ R_{n+1}^{\varepsilon ,\eta }(s) , \textrm{e}^{\mathcal {L}_{S_n^{\varepsilon ,\eta }(s)}} \circ \tau _{t,s}\llbracket B \rrbracket \Bigr ] \Bigr \rangle _{\rho _0} \biggr |\\  &\le C \, \Vert \varPhi _{R_{n+1}^{\varepsilon ,\eta }}(s) \Vert _{b',p'; {\varLambda ^{\textrm{pert}}}} \, \textrm{e}^{c \, \Vert \varPhi _S \Vert _{d,q}} \, \bigl ( 1+{{\,\textrm{diam}\,}}(Y) \bigr )^{3d} \, \Vert B \Vert \, \delta ^{2d/p} . \end{aligned}$$

We recall, that like the $R_j^{\varepsilon ,\eta }(s)$, also the $\tilde{R}_j^{\varepsilon ,\eta }(s)$ are polynomials of degree j-1 in $\eta /\varepsilon$ with ${\varLambda ^{\textrm{pert}}}$-localized SLT operators as coefficients. Hence, the interaction norms in the above equations can be bounded by a constant depending on the interaction norms of $H_0$ and V(t) times $1+(\eta /\varepsilon )^{j-1}$. With this observation, we can insert these bounds into (88) and conclude (79).

To reduce the number of constants in the formulation of the statement, we actually do the proof for $(p+p^{\prime })/2$ instead of $p^{\prime }$. In the end we then estimate $$\chi _{b',(p+p')/2} \le C \, \chi _{1,p'}$$ for the locality of the operators $$A^{\varepsilon ,\eta }_\mu (t)$$, similarly to (89). □

## Data Availability

Data sharing is not applicable to this article as no new data were created or analyzed in this study.

## Technical Lemmata

In this section, we prove the technical lemmata required for the construction of the NEASS. We begin with some general properties of the functions $\chi _{b,p}$ and Lieb-Robinson bounds for (b,p)-localized SLT-operators in Appendix A.1. In Appendix A.2, we prove that the various operations used in the construction of the NEASS preserve locality. In Appendix A.3 we recall the construction of the quasi-local inverse of the Liouvillian and prove that it also preserves locality of SLT-operators. Finally, Appendices A.4 and A.5 are concerned with the proofs of Lemmata 2.1 and 6.1, respectively.

In all proofs, C>0 is a generic constant that might change within the computations. It can in particular depend on all the parameters chosen in the statements, but it is uniform in the chosen lattice and the operators appearing.

### Properties of the Decay Function $\chi _{b,p}$

Let us first collect some properties of the decay function $\chi _{b,p}$ we use in the definition of the interaction norm. From [38, Lemma 7.2.3] we have the following Lemma, where we simplified the statements.

#### Lemma A.1

For any b≥0 and p∈(0,1], the function $\chi _{b,p}$ satisfies the following properties:

- $\chi _{b,p}$ is logarithmically superadditive, i.e. $\chi _{b,p}(x+y) \ge \chi _{b,p}(x) \, \chi _{b,p}(y)$ for all x, y≥0.
- For every b>0 and k≥0 there exists a constant C>0 such that
  $$\begin{aligned} \sup _{x \ge 0} x^k \, \chi _{b,p}(x) = C . \end{aligned}$$

As a direct consequence of Lemma A.1 (b), we get the following Lemma, which we write out to fix the constant and recall it in later proofs.

#### Lemma A.2

Let $d\in \mathbb {N}$, $C_{\textrm{vol}}>0$, b>0, p∈(0,1] and $k\in \mathbb {N}$. Then there exists a constant $\mathcal {C}_{\textrm{vol},b,p,k}>0$ such that for all lattices $\varLambda \in \mathcal {G}(d,C_{\textrm{vol}})$ and sets $Z\subset \varLambda$

$$\begin{aligned} |Z |^k \, \chi _{b,p}\bigl ( {{\,\textrm{diam}\,}}( Z ) \bigr ) \le \mathcal {C}_{\textrm{vol},b,p,k} . \end{aligned}$$

Before we state the Lieb-Robinson bound, which is a crucial ingredient in the proof, let us briefly recall the time-dependent Heisenberg evolution. For a time-dependent interaction defined on an interval $I\subset \mathbb {R}$ with corresponding SLT operator H(t), let $\tau _{t,s}$ be the unique solution of

$$\begin{aligned} -\textrm{i}\, \frac{\textrm{d}}{\textrm{d}t} \, \tau _{t,s}(A) = \tau _{t,s}\bigl ( [ H(t), A ] \bigr ) \quad \quad \text {and}\quad \quad \tau _{s,s} = \operatorname {Id} \qquad \text {for all } s \text {, } t\in I . \end{aligned}$$

This was already used with a different time scaling for the Hamiltonian $H_\varepsilon (t)$ in the proof of Theorem 6.2. Under locality assumptions on the Hamiltonian, one finds the following Lieb-Robinson bound.

#### Lemma A.3

(Lieb-Robinson bound [38, Theorem 7.3.3]) Let $$d\in \mathbb {N} $$, $$C_{\textrm{vol}}>0$$, $b^{\prime }>b>0$, p∈(0,1] and $$k\in \mathbb {N} $$. There exist constants C and c>0 such that for all lattices $$\varLambda \in \mathcal {G}(d,C_{\textrm{vol}})$$, intervals $$I\subset \mathbb {R} $$, time-dependent interactions Φ, disjoint subsets X, $$Y \subset \varLambda $$, observables $$A\in \mathcal {A}_X$$ and $$B\in \mathcal {A}_Y$$, and s, t∈I it holds that

90 $$\begin{aligned} \bigl \Vert \bigl [ \tau _{t,s}(A),B \bigr ] \bigr \Vert \le C \, \Vert A \Vert \, \Vert B \Vert \, \bigl ( \textrm{e}^{b \, v \, |t-s |}-1 \bigr ) \, D(X,Y) , \end{aligned}$$

where $v = c \, \Vert \varPhi \Vert _{b',p} / b$ is the Lieb-Robinson velocity and

$$\begin{aligned} D(X,Y)&:= \min \biggl \{ \sum _{x\in X} \chi _{b,p}\bigl ( d( x,Y ) \bigr ), \sum _{y\in Y} \chi _{b,p}\bigl ( d( y,X ) \bigr ) \biggr \} \\  &\phantom {:}\le \min \bigl \{ |X |,|Y |\bigr \} \, \chi _{b,p}\bigl ( d( X,Y ) \bigr ) . \end{aligned}$$

The Lieb-Robinson velocity is defined including the 1/b because (90) can be bounded by

$$\begin{aligned} C \, \Vert A \Vert \, \Vert B \Vert \, \min \bigl \{ |X |,|Y |\bigr \} \, \textrm{e}^{b \, \bigl ( v \, |t-s |- d( X,Y )^p \bigr )} . \end{aligned}$$

### Commutators and Dynamics of Localized SLT-Operators

#### Lemma A.4

(Commutator with local observable) Let $d\in \mathbb {N}$, $C_{\textrm{vol}}>0$, b>0, p∈(0,1] and $k\in \mathbb {N}$. There exist constants C and $C_k>0$ such that for all lattices $\varLambda \in \mathcal {G}(d,C_{\textrm{vol}})$, subsets Ω, $X \subset \varLambda$, SLT operators $A_1$ and observables $O\in \mathcal {A}_X$ it holds that

91 $$\begin{aligned} \bigl \Vert [ A_1,O ] \bigr \Vert \le 2 \, \Vert O \Vert \, |X |\, \chi _{b,p}\bigl ( d( X,\varOmega ) \bigr ) \, \Vert \varPhi _{A_1} \Vert _{b,p;\varOmega } . \end{aligned}$$

For a second observable $\tilde{O} \in \mathcal {A}_Y$, it holds that

92 $$\begin{aligned} \bigl \Vert [ [ A_1, O ], \tilde{O} ] \bigr \Vert \le 4 \, \Vert O \Vert \, \Vert \tilde{O} \Vert \, |X |\, \chi _{b,p}\bigl ( d( X,Y ) \bigr ) \, \chi _{b,p} \bigl ( d( X,\varOmega ) \bigr )\, \Vert \varPhi _{A_1} \Vert _{b,p;\varOmega } . \end{aligned}$$

Finally, if additionally also $A_2,\dotsc ,A_k$ are SLT operators, then

93 $$\begin{aligned} \bigl \Vert {{\,\textrm{ad}\,}}_{A_k}\cdots {{\,\textrm{ad}\,}}_{A_1}(O) \bigr \Vert \le C_k \, \Vert O \Vert \, |X |^k \, \chi _{b,p}\bigl ( d( X,\varOmega ) \bigr ) \, \Vert \varPhi _{A_1} \Vert _{b,p;\varOmega } \, \prod _{j=2}^k \, \Vert \varPhi _{A_j} \Vert _{b,p} . \end{aligned}$$

All three bounds, in particular, also hold for $\varOmega =\varLambda$, where $\Vert \,\cdot \,\Vert _{b,p;\varOmega } = \Vert \,\cdot \,\Vert _{b,p}$ and d(X,Ω)=0.

#### Proof

We begin with the first statement and write $A=A_1$. Since $[ \varPhi _A(Z),O ]$ vanishes whenever $Z\cap X=\varnothing$, we find

where we just overcount in the second inequality. Clearly, the same statement also holds with $\varOmega =\varLambda$. The proof of (92) is analogous to the proof of (91) and so omitted.

We conclude by proving (93) using induction. Note that the outer operators are all SLT-operators on Λ. The k=1 case is given in (91). We now assume (93) for some fixed k and with $\varOmega =\varLambda$. Then, we add a further commutator with $A_0$ to conclude

where we used Lemma A.2 in the last step. This finishes the induction. □

#### Lemma A.5

(Multi-commutators) Let $d\in \mathbb {N}$, $C_{\textrm{vol}}>0$, b>0, p∈(0,1], ε>0 and $k\in \mathbb {N}$. There exists a constant C>0 such that for all lattices $\varLambda \in \mathcal {G}(d,C_{\textrm{vol}})$, subsets $\varOmega \subset \varLambda$ and SLT operators $A_0,\dotsc ,A_k$ it holds that

94 $$\begin{aligned} \bigl \Vert \varPhi _{{{\,\textrm{ad}\,}}_{A_k}\cdots {{\,\textrm{ad}\,}}_{A_1}(A_0)} \bigr \Vert _{b,p;\varOmega } \le C \, \Vert \varPhi _{A_0} \Vert _{b+\varepsilon ,p;\varOmega } \, \prod _{j=1}^k \, \Vert \varPhi _{A_j} \Vert _{2b+\varepsilon ,p} . \end{aligned}$$

#### Proof

For the proof we first need to construct an interaction for the commutator of two SLT operators A and B. It turns out that it can be given as

Then,

where we used ${{\,\textrm{diam}\,}}(Z) \le {{\,\textrm{diam}\,}}(X) + {{\,\textrm{diam}\,}}(Y)$ and the properties of $\chi _{b,p}$. The above sum can be bounded by the sum of the terms where z∈X or z∈Y. The latter can be upper bounded by

where we used Lemma A.2 in the third step. Using $\chi _{b,p}\bigl ( d( z,\varOmega ) \bigr ) \ge \chi _{b,p}\bigl ( {{\,\textrm{diam}\,}}( X ) \bigr ) \, \chi _{b,p} \bigl ( d( x,\varOmega ) \bigr )$ for all z,x∈X, the part of the sum where z∈X can be bounded by

Both bounds together prove the claim for k=1. To proceed by induction we assume that the statement holds for some fixed k. Then, applying first the statement for k=1 and then k=k both with ε/2 we obtain

$$\begin{aligned} \bigl \Vert \varPhi _{{{\,\textrm{ad}\,}}_{A_{k+1}}\cdots {{\,\textrm{ad}\,}}_{A_1}(A_0)} \bigr \Vert _{b,p;\varOmega }&\le C \, \Vert \varPhi _{{{\,\textrm{ad}\,}}_{A_{k}}\cdots {{\,\textrm{ad}\,}}_{A_1}(A_0)} \Vert _{b+\varepsilon /2,p;\varOmega } \, \Vert \varPhi _{A_{k+1}} \Vert _{2b+\varepsilon /2,p} \\  &\le C \, \Vert \varPhi _{A_0} \Vert _{b+\varepsilon ,p;\varOmega } \, \prod _{j=1}^{k+1} \, \Vert \varPhi _{A_j} \Vert _{2b+\varepsilon ,p} . \end{aligned}$$

□

For the following statements we need to approximate the time evolution of local operators, which in principle live on the whole lattice. This can be done by a so-called conditional expectation, which is just the partial trace in our case of finite spin systems. We collect its properties in the following lemma.

#### Lemma A.6

([44, Lemma 4.1]) Let Λ be a lattice and $X\subset \varLambda$. Then there exists a unit-preserving, completely positive linear map $\mathbb {E}_{X}:\mathcal {A}_\varLambda \rightarrow \mathcal {A}_\varLambda$ satisfying

- $$\mathbb {E}_{X}( \mathcal {A}_\varLambda )\subset \mathcal {A}_X$$;
- $\mathbb {E}_{X}( ABC ) = A \, \mathbb {E}_{X}( B ) \, C$ for all $B\in \mathcal {A}_\varLambda$ and $A,C\in \mathcal {A}_{X}$; This in particular implies $\mathbb {E}_{X}( A )=A$ for all $A\in \mathcal {A}_{X}$;
- $$\Vert \mathbb {E}_{X} \Vert =1$$;
- $$\mathbb {E}_{X} \circ \mathbb {E}_{Y} = \mathbb {E}_{X\cap Y}$$, for $$X,Y\subset \varLambda $$;
- If $$A\in \mathcal {A}_\varLambda $$ satisfies
  95 $$\begin{aligned} \bigl \Vert [ A,B ] \bigr \Vert \le \eta \, \Vert A \Vert \, \Vert B \Vert \quad \text {for all}\quad B\in \mathcal {A}_{\varLambda \setminus X} , \end{aligned}$$
  for some η>0, then
  96 $$\begin{aligned} \Vert A-\mathbb {E}_{X}( A ) \Vert \le \eta \, \Vert A \Vert . \end{aligned}$$

Together with the Lieb-Robinson bound we can now obtain the following.

#### Lemma A.7

(Dynamics) Let $$d\in \mathbb {N} $$, $$C_{\textrm{vol}}>0$$, $b,b^{\prime }>0$, and $p,p^{\prime }\in \left(0,1\right]$ satisfying $p^{\prime }<p$ or $p^{\prime }=p$ and $2\;b^{\prime }<b$. There exist constants C and c>0 such that for all lattices $$\varLambda \in \mathcal {G}(d,C_{\textrm{vol}})$$, the following holds: Let $$I\subset \mathbb {R} $$ an interval, the interaction Φ generate the dynamics $$\tau _{t,s}$$ with Lieb-Robinson velocity $$v = c \, \Vert \varPhi \Vert _{b,p} / b$$. For every $$\chi _{b',p'}$$-SLT operator A, subsets Ω, $$X\subset \varLambda $$, observables $$O\in \mathcal {A}_X$$, and t,s∈I it holds that

$$\begin{aligned} \Bigl \Vert \bigl [ A,\tau _{t,s}(O) \bigr ] \Bigr \Vert \le C \, \Vert O \Vert \, |X |\, |X_{(2v \, |t-s |)^{1/p}} |\, \Vert \varPhi _A \Vert _{b',p';\varOmega } \, \chi _{b',p'}\bigl ( d( X_{(2v \, |t-s |)^{1/p}},\varOmega ) \bigr ) \end{aligned}$$

#### Proof

We use the local decomposition technique similar to [44, Section 5]. Therefore, let

$$\begin{aligned} \varDelta _0&:= \mathbb {E}_{X_{(v \, |t-s |)^{1/p}}}\bigl ( \tau _{t,s}(O) \bigr )\\ \end{aligned}$$

and

$$\begin{aligned} \varDelta _k&:= \mathbb {E}_{X_{(v \, |t-s |+k)^{1/p}}}\bigl ( \tau _{t,s}(O) \bigr ) - \mathbb {E}_{X_{(v \, |t-s |+k-1)^{1/p}}}\bigl ( \tau _{t,s}(O) \bigr ) , \end{aligned}$$

so that $\tau _{t,s}(O)=\sum _{k=0}^\infty \varDelta _k$, where the sum is finite since eventually $X_{(v \, |t-s |+k)^{1/p}}=\varLambda$. By the properties of the conditional expectation

$$\begin{aligned} \Vert \varDelta _0 \Vert&\le \Vert \tau _{t,s}(O) \Vert = \Vert O \Vert \end{aligned}$$

and for k≥1 it holds that

$$\begin{aligned} \varDelta _k&= \mathbb {E}_{X_{(v \, |t-s |+k)^{1/p}}}\Bigl ( \bigl ( 1-\mathbb {E}_{X_{(v \, |t-s |+k-1)^{1/p}}} \bigr )\,\tau _{t,s}(O) \Bigr ) \end{aligned}$$

and thus

$$\begin{aligned} \Vert \varDelta _k \Vert&\le \bigl \Vert \bigl ( 1-\mathbb {E}_{X_{(v \, |t-s |+k-1)^{1/p}}} \bigr )\,\tau _{t,s}(O) \bigr \Vert . \end{aligned}$$

Furthermore, for all $B\in \mathcal {A}_{\varLambda {\setminus } X_{(v \, |t-s |+k-1)^{1/p}}}$ by the Lieb-Robinson bound (Lemma A.3)

$$\begin{aligned} \bigl \Vert [ \tau _{t,s}(O),B ] \bigr \Vert \le C_\textrm{LR}\, \Vert O \Vert \, \Vert B \Vert \, |X |\, \textrm{e}^{b\bigl ( v \, |t-s |-(v \, |t-s |+k-1) \bigr )} = C_\textrm{LR}\, \textrm{e}^{b} \, \Vert O \Vert \, \Vert B \Vert \, |X |\, \textrm{e}^{-b k} \end{aligned}$$

because ${d( X,\varLambda {\setminus } X_{(v \, |t-s |+k-1)^{1/p}} )}\ge (v \, |t-s |+k-1)^{1/p}$, and thus by Lemma A.6

$$\begin{aligned} \Vert \varDelta _k \Vert \le C_\textrm{LR}\, \textrm{e}^{b} \, \Vert O \Vert \, |X |\, \textrm{e}^{-b k} . \end{aligned}$$

Now we can apply Lemma A.4 to each of the summands in the decomposition

$$\begin{aligned}&\bigl \Vert \bigl [ A,\tau _{t,s}(O) \bigr ] \bigr \Vert \le \sum _{k=0}^\infty \, \bigl \Vert \bigl [ A,\varDelta _k \bigr ] \bigr \Vert \\  &\le \sum _{k=0}^\infty 2 \, \Vert \varDelta _k \Vert \, \bigl |X_{(v \, |t-s |+k)^{1/p}} \bigr |\, \chi _{b',p'}\bigl ( d( X_{(v \, |t-s |+k)^{1/p}},\varOmega ) \bigr ) \, \Vert \varPhi _A \Vert _{b',p';\varOmega } \\  &\le \tilde{C} \, \Vert O \Vert \, |X |\, \Vert \varPhi _A \Vert _{b',p';\varOmega } \sum _{k=0}^\infty \bigl |X_{(v \, |t-s |+k)^{1/p}} \bigr |\, \chi _{b',p'}\bigl ( d( X_{(v \, |t-s |+k)^{1/p}},\varOmega ) \bigr ) \, \textrm{e}^{-b k} \\  &\le \begin{aligned}&\tilde{C} \, \Vert O \Vert \, |X |\, |X_{(2v \, |t-s |)^{1/p}} |\, \Vert \varPhi _A \Vert _{b',p';\varOmega } \, \chi _{b',p'}\bigl ( d( X_{(2v \, |t-s |)^{1/p}},\varOmega ) \bigr ) \\  &\times \sum _{k=0}^\infty ( 1+C_{\textrm{vol}}\, (2k)^{d/p} ) \, \chi _{b',p'}\bigl ( (2k)^{1/p} \bigr )^{-1} \, \textrm{e}^{-b k}, \end{aligned} \end{aligned}$$

where we abbreviated $$\tilde{C} = 2 \max \{ 1,C_\textrm{LR}\, \textrm{e}^{b} \}$$, used $$(v \, |t-s |+k)^{1/p} \le (2v \, |t-s |)^{1/p} + (2k)^{1/p}$$. To conclude the result, we observe that the series is bounded for $p^{\prime }<p$ or $2\;b^{\prime }<b$ if $p^{\prime }=p$. □

#### Lemma A.8

(Conjugation with unitaries) Let $d\in \mathbb {N}$, $C_{\textrm{vol}}>0$, ε>0, a, b>0, and p,q∈(0,1] satisfying p<q or p=q and $a > (2^p+1) \, b$. There exist constants C and c>0 such that for all lattices $\varLambda \in \mathcal {G}(d,C_{\textrm{vol}})$, the following holds: For SLT operators D and S and any $\varOmega \subset \varLambda$ it holds that $A:= \textrm{e}^{\textrm{i}\, S}\,D\,\textrm{e}^{-\textrm{i}\, S}$ is an SLT operator as well. More precisely, there exists an interaction $\varPhi _A$ such that

$$\begin{aligned} \Vert \varPhi _A \Vert _{b,p;\varOmega } \le C \, \textrm{e}^{c \, \Vert \varPhi _S \Vert _{a,q}} \, \bigl \Vert \varPhi _D \bigr \Vert _{b+\varepsilon ,p;\varOmega } \end{aligned}$$

#### Proof

The proof uses the same technique as the proof of Lemma A.7.

First fix $X\subset \varLambda$ and $O\in \mathcal {A}_X$ and denote $\tau (O) = \textrm{e}^{\textrm{i}S}\,O\,\textrm{e}^{-\textrm{i}S}$. Then define

$$\begin{aligned} \varDelta _0(O)&:= \mathbb {E}_{X}\bigl ( \tau (O) \bigr )\\ \end{aligned}$$

and

$$\begin{aligned} \varDelta _k(O)&:= \mathbb {E}_{X_k}\bigl ( \tau (O) \bigr ) - \mathbb {E}_{X_{k-1}}\bigl ( \tau (O) \bigr ) = \mathbb {E}_{X_k}\Bigl ( \bigl ( {{\,\textrm{id}\,}}- \mathbb {E}_{X_{k-1}} \bigr ) \, \tau (O) \Bigr ) . \end{aligned}$$

By properties of the conditional expectation, Lemma A.6, and the Lieb-Robinson bound, Lemma A.3, one can bound

$$\begin{aligned} \Vert \varDelta _0(O) \Vert \le \Vert O \Vert \end{aligned}$$

and

97 $$\begin{aligned} \Vert \varDelta _k(O) \Vert \le C_\textrm{LR}\, \Vert O \Vert \, |X |\, \bigl ( \textrm{e}^{c \, \Vert \varPhi _S \Vert _{a,q}}-1 \bigr ) \, \chi _{a',q}(k) \end{aligned}$$

for k≥1 because $$d( X,\varLambda {\setminus } X_{k-1} )=k$$ in our geometry, where we chose $a^{\prime }<a$.

We now construct an interaction for A. First note, that

where $\varDelta _k\bigl ( \varPhi _D(Z) \bigr )\in \mathcal {A}_{Z_k}$ and the sum is actually finite. For any function $f:\{ \, Y \subset \varLambda \, \} \rightarrow \mathcal {A}_{\varLambda }$ and k≥0 it holds that

Applying this with $f:Z \mapsto \varDelta _k(\varPhi _D(Z))$ for each k we find

With this interaction for A and any $z\in \varLambda$ we bound

The k=0 term is bounded by $\Vert \varPhi _D \Vert _{b,p;\varOmega }$. For k≥1 we use (97). Moreover, ${{\,\textrm{diam}\,}}(Y_k) \le {{\,\textrm{diam}\,}}(Y) + 2k$ and, since $z\in Y_k$, there exists $y\in B_k(z) \cap Y$, such that $d( z,\varOmega ) \le k + d( y,\varOmega )$. Hence, the remaining sum is bounded by

for some C>0. The remaining sum is bounded if q>p, or q=p and $$a'>(2^p+1)\,b$$. The last condition is equivalent to $$a>(2^p+1)\,b$$, by our choice of $a^{\prime }$. To conclude the statement, we choose the total constant larger than 1 and add the k=0 term $$ \bigl \Vert \varPhi _D \bigr \Vert _{b,p;\varOmega } \le \bigl \Vert \varPhi _D \bigr \Vert _{b+\varepsilon ,p;\varOmega } $$. □

### Quasi-local Inverse of the Liouvillian

In this section, we briefly recall the construction of the quasi-local inverse of the Liouvillian $\mathcal {I}$ and the related operator $\mathcal {J}$ introduced in (21) and (26). Both of them use certain properties of a *filter function* $w_g$, which one can construct explicitly.

#### Lemma A.9

(Explicit filter function, cf. Lemma 2.3 from [1]) Let g>0 and consider the sequence $(a_n)_{n \ge 1}$ of positive numbers, defined as $a_n = a_1 (n (\log n)^2)^{-1}$ for n≥2 and $a_1$ chosen such that $\sum _{n \ge 1} a_n = g/2$. Then, the positive function $w_g \in L^1( \mathbb {R} )$ defined via the infinite product

98 $$\begin{aligned} w_g(t) := c_g \prod _{n=1}^{\infty } \left( \frac{\sin (a_nt)}{a_nt} \right) ^2 \end{aligned}$$

and $c_g > 0$ chosen such that $\int _ \mathbb {R} \textrm{d}t \, w_g(t) = 1$, has Fourier transform $\widehat{w_g}$ with compact support ${{\,\textrm{supp}\,}}(\widehat{w_g}) \subset [-g,g]$ (cf. (22)) and satisfies the bound $|w_g(t) |\le C \, \textrm{e}^{-|t |^q}$ for every q<1 (cf. (23)).

Given the explicit filter function (98), the quasi-local inverse of the Liouvillian $\mathcal {I}_{H,g} \llbracket \,\cdot \,\rrbracket : \mathcal {A}\rightarrow \mathcal {A}$ of the Hamiltonian H with gap parameter g>0, acting on $A \in \mathcal {A}$, is then defined as

99 $$\begin{aligned} \mathcal {I}_{H,g}\llbracket A \rrbracket := \int _{ \mathbb {R} } \textrm{d}t \, w_g(t) \int _{0}^{t} \textrm{d}s \, \textrm{e}^{\textrm{i}Hs} \, A \, \textrm{e}^{-\textrm{i}Hs} . \end{aligned}$$

#### Remark A.10

*(On the filter function)* We point out, that, in principle and unless additional conditions are given, any map $\mathcal {I}_{H,g}$ with the properties (21)–(23) would work for all of our proofs in this paper, in particular including the statements from Sect. 3.

Together with the map $\mathcal {J}_{H,g} :\mathcal {A}\rightarrow \mathcal {A}$, again depending on the Hamiltonian H and gap parameter g>0, with action on $A \in \mathcal {A}$ defined as

$$\begin{aligned} \mathcal {J}_{H,g}\llbracket A \rrbracket := - \int _{ \mathbb {R} } \textrm{d}t \, w_g(t) \, \textrm{e}^{\textrm{i}Ht} \, A \, \textrm{e}^{- \textrm{i}Ht} , \end{aligned}$$

one then has (recalling the Liouvillian $\mathcal {L}_H\llbracket \,\cdot \,\rrbracket = -\textrm{i}\, [H, \,\cdot \,]$) the identity $\mathcal {L}_H \circ \mathcal {I}_{H,g}\llbracket A \rrbracket - A = \mathcal {J}_{H,g}\llbracket A \rrbracket$ for all $A \in \mathcal {A}$; see (26).

The inverse Liouvillian $\mathcal {I}_{H,g}$ is called *quasi-local*, since, if H satisfies the Lieb-Robinson bound from Lemma A.3, then it holds that, for $A \in \mathcal {A}_X$ and $B \in \mathcal {A}_Y$ (see e.g. [44, Example 5.7])

100 $$\begin{aligned} \bigl \Vert \bigl [ \mathcal {I}_{H,g}\llbracket A \rrbracket , B \bigr ] \bigr \Vert \le C \, \Vert A \Vert \, \Vert B \Vert \, \min \{ |X |, |Y |\} \chi _{\tilde{b}, \tilde{p}}\bigl ( d( X, Y ) \bigr ) \end{aligned}$$

for some $\tilde{b} > 0$ (depending on the Lieb-Robinson velocity v from Lemma A.3) and $\tilde{p} \in (0,1)$, which can be chosen as p from (90) if p<1. The estimate (100) holds verbatim with $\mathcal {I}$ replaced by $\mathcal {J}$.

Beside the classical quasi-locality estimate (100), the inverse Liouvillian even preserves locality of SLT operators. This is the content of the following lemma, the special case for p=1 without localization (i.e. for Λ-localized SLT operators) already appeared in [38, Theorem 7.5.6] and is based on [1, Theorem 4.8].

#### Lemma A.11

(Quasi-local inverse of the Liouvillian on SLT operators) Let $d\in \mathbb {N}$, $C_{\textrm{vol}}>0$, ε>0, a, b>0, g>0, p, q∈(0,1] satisfying q<p, and $C_{\textrm{int}}>0$. There exists a constant C>0 such that for all lattices $\varLambda \in \mathcal {G}(d,C_{\textrm{vol}})$, the following holds: For SLT operators H and D and any $\varOmega \subset \varLambda$ it holds that $\mathcal {I}_{H,g}(D)$ is an SLT operator as well. More precisely if $\Vert \varPhi _{H} \Vert _{b,p} \le C_{\textrm{int}}$, then there exists an interaction $\varPhi _{\mathcal {I}_{H,g}(D)}$ such that

$$\begin{aligned} \Vert \varPhi _{\mathcal {I}_{H,g}(D)} \Vert _{a,q;\varOmega } \le C \, \bigl \Vert \varPhi _D \bigr \Vert _{a+\varepsilon ,q;\varOmega } . \end{aligned}$$

The statement holds verbatim when replacing $\mathcal {I}$ by $\mathcal {J}$ from (26).

In the proof of the Lemma we use the equality

$$\begin{aligned} \mathcal {I}_{H,g}\llbracket A \rrbracket = \int _{ \mathbb {R} } \textrm{d}t \, \mathcal {W}_g(t) \, \textrm{e}^{\textrm{i}Ht} A \textrm{e}^{-\textrm{i}Ht} , \end{aligned}$$

where the function $\mathcal {W}_g$ is given by $\mathcal {W}_g(t) = - \int _{- \infty }^{t} \textrm{d}s \, w_g(s) + \textbf{1}_{[0, \infty )}(t)$ with $\textbf{1}_{[0,\infty )}$ being the characteristic function of [0,∞). In many works, e.g. [1, 3, 40, 44, 49], this is used as a definition for the inverse Liouvillian. It can easily be checked that, by Lemma A.9, also $\mathcal {W}_g$ satisfies $|\mathcal {W}_g(t) |\le C \, \textrm{e}^{-|t |^q}$.

#### Proof

The proof uses the same technique as the proofs of Lemma A.7 and A.8.

First fix $X\subset \varLambda$ and $O\in \mathcal {A}_X$ and denote $\tau (O) = \textrm{e}^{\textrm{i}H}\,O\,\textrm{e}^{-\textrm{i}H}$. Then, define

$$\begin{aligned} \varDelta _0(O)&= \int _{ \mathbb {R} } \textrm{d}s \, \mathcal {W}_g(s) \, \mathbb {E}_{X}\bigl ( \tau _s(O) \bigr ) \end{aligned}$$

and

$$\begin{aligned} \varDelta _k(O)&= \int _{ \mathbb {R} } \textrm{d}s \, \mathcal {W}_g(s) \, \Bigl ( \mathbb {E}_{X_{k}}\bigl ( \tau _s(O) \bigr ) - \mathbb {E}_{X_{k-1}}\bigl ( \tau _s(O) \bigr ) \Bigr ) \\  &= \int _{ \mathbb {R} } \textrm{d}s \, \mathcal {W}_g(s) \, \mathbb {E}_{X_{k}}\Bigl ( \bigl ( {{\,\textrm{id}\,}}- \mathbb {E}_{X_{k-1}} \bigr ) \bigl ( {\tau _s(O)} \bigr ) \Bigr ) \end{aligned}$$

for k≥1. Then $\mathcal {I}_{H,g}(O)=\sum _{k \ge 0} \varDelta _k(O)$ where the sum is eventually finite.

For k=0 we have

$$\begin{aligned} \Vert \varDelta _0(O) \Vert \le \Vert \mathcal {W}_g \Vert _{L^1} \, \Vert O \Vert . \end{aligned}$$

For $b^{\prime }\in \left(0,b\right)$, k≥1 and some T>0 to be chosen, Lemma A.3 and the properties of the conditional expectation, yield

$$\begin{aligned} \hspace{2em}&\hspace{-2em} \biggl \Vert \int _{-T}^T \textrm{d}s \, \mathcal {W}_g(s) \, \mathbb {E}_{X_{k}}\Bigl ( \bigl ( {{\,\textrm{id}\,}}- \mathbb {E}_{X_{k-1}} \bigr ) \bigl ( {\tau _s(O)} \bigr ) \Bigr ) \biggr \Vert \\  &\le \Vert \mathcal {W}_g \Vert _{L^\infty } \int _{-T}^T \textrm{d}s \, \bigl \Vert \bigl ( {{\,\textrm{id}\,}}- \mathbb {E}_{X_{k-1}} \bigr ) \bigl ( {\tau _s(O)} \bigr ) \bigr \Vert \\  &\le C_\textrm{LR}\, \Vert O \Vert \, |X |\, \Vert \mathcal {W}_g \Vert _{L^\infty } \, \int _{-T}^T \textrm{d}s \, ( \textrm{e}^{b' \, v \, |s |} - 1 ) \, \chi _{b',p}(k) \\  &= 2 \, C_\textrm{LR}\, \Vert O \Vert \, |X |\, \Vert \mathcal {W}_g \Vert _{L^\infty } \frac{\textrm{e}^{b' v T} - b' v T - 1}{b' \, v} \, \chi _{b',p}(k) \\  &\le 2 \, C_\textrm{LR}\, \Vert O \Vert \, |X |\, \Vert \mathcal {W}_g \Vert _{L^\infty } \, \chi _{b'/2,p}(k) / (b' \, v) \end{aligned}$$

where we chose $T=k^p/(2v)$ for the last step. Furthermore, by Lemma A.9 and after integrating twice, for any $0<\tilde{p}<1$ there exists C and $\tilde{b}>0$ such that

$$\begin{aligned} \hspace{2em}&\hspace{-2em} \biggl \Vert \int _{|s |\ge T} \textrm{d}s \, \mathcal {W}_g(s) \, \mathbb {E}_{X_{k}}\Bigl ( \bigl ( {{\,\textrm{id}\,}}- \mathbb {E}_{X_{k-1}} \bigr ) \bigl ( {\tau _s(O)} \bigr ) \Bigr ) \biggr \Vert \\  &\le 2 \, \Vert O \Vert \int _{|s |\ge T} \textrm{d}s \, |\mathcal {W}_g(s) |\\  &\le C \, \chi _{\tilde{b},\tilde{p}}(T) \\  &\le C \, \chi _{\tilde{b}',\tilde{p}p}(k) , \end{aligned}$$

where $$\tilde{b}'=\bigl ( 1/(2v) \bigr )^{\tilde{p}} \, \tilde{b}$$. Then, for all $p^{\prime }\in \left(0,p\right)$ we can choose $$\tilde{p}=p'/p<1$$ and combine the two bounds. Then, there exists C and η>0 such that

101 $$\begin{aligned} \Vert \varDelta _k(O) \Vert \le C \, |X |\, \Vert O \Vert \, \chi _{\eta ,p'}(k) \quad \quad \text {for all}\quad \quad k \ge 0 . \end{aligned}$$

An interaction for $A:=\mathcal {I}_{H,g}(D)$ is given by

It follows that

The k=0 term is bounded by $\Vert \mathcal {W}_g \Vert _{L^1} \Vert \varPhi _D \Vert _{a,q;\varOmega }$. For k≥1 and $z\in Y_k$ there exists $y\in B^{\varLambda }_k(z)\cap Y$ such that $d( z,\varOmega )\le k+d( y,\varOmega )$. Furthermore, ${{\,\textrm{diam}\,}}(Y_k) \le 2k+{{\,\textrm{diam}\,}}(Y)$. Hence, using (101) the rest of the sum is bounded by

The remaining sum is bounded if $p^{\prime }>q$, which we can ensure if p>q by choosing $p^{\prime }\in \left(q,p\right)$. Thus, there exists C such that

for some constant C depending on $\Vert \varPhi _H \Vert _{b,p}$, p, b, q, a, ε and d which finishes the proof. □

#### Remark A.12

*(Abstract properties of* $\mathcal {I}$ *needed in the proof of our main result)* For the purpose of proving our main result, Theorem 4.1, it is not necessary to work with the explicit $\mathcal {I}_{H,g}$ from (99). In fact, by inspecting the proof of Proposition 4.4 in Sect. 6, which is the key input for our main result, we realize the following: For Theorem 4.1 being valid (up to minor adjustments of constants), one only needs that there exists some operator $\widetilde{\mathcal {I}}: \mathcal {A}\rightarrow \mathcal {A}$ for which Assumption $(\textrm{LDG}_{\textrm{informal}})$ is satisfied and such that

(i) for $A \in \mathcal {A}_X$ and $B \in \mathcal {A}_Y$ it holds that (cf. (104))
  $$\begin{aligned} \Bigl |\bigl \langle \bigl [ \mathcal {L}_{H} \circ \widetilde{\mathcal {I}}\llbracket A \rrbracket - A , B \bigr ] \bigr \rangle _{\rho _0} \Bigr |\le C \, \Vert A \Vert \, \Vert B \Vert \, \bigl ( 1+{{\,\textrm{diam}\,}}(X) \bigr )^\ell \exp (-d( X,Y )^q) \end{aligned}$$
  for some positive constants C,q,ℓ>0, i.e. the composition $\mathcal {L}_{H} \circ \widetilde{\mathcal {I}}$ behaves as a quasi-local operator if tested in the above way;
(ii) Lemma A.11 holds, i.e. $\widetilde{\mathcal {I}}$ maps localized SLT operators to localized SLT operators.

These relaxed abstract conditions are, however, not sufficient for showing the relations among the various gap conditions outlined in Sect. 3.

### Localized SLT Operators: Proof of Lemma 2.1

For (i), since Φ is strictly Ω-localized, $\varPhi (Z) \ne 0$ only if $Z\subset \varOmega$ and then $\chi _{b,p}\bigl ( d( z,\varOmega ) \bigr )=1$ for all z∈Z. Hence,

Next, for (ii) and strongly Ω-localized Φ, we have that

since $d( z,\varOmega ) \le {{\,\textrm{diam}\,}}(Z)$ for z∈Z and $Z \cap \varOmega \ne \varnothing$, together with monotonicity of $\chi _{b/2, p}$ and using $(\chi _{b/2, p})^2 = \chi _{b, p}$. This concludes the proof of Lemma 2.1.

□

### Assumption $(\textrm{LDG}_{\textrm{weak}})$ for SLT Operators: Proof of Lemma 6.1

We write $A = \sum _{Z \subset \varLambda } \varPhi _A(Z)$ (cf. (15)) and estimate

102 $$\begin{aligned} \begin{aligned} \hspace{2em}&\hspace{-2em} \Bigl |\bigl \langle \bigl [ \mathcal {L}_{H_0} \circ \mathcal {I}_{H_0,g}\llbracket A \rrbracket - A , B \bigr ] \bigr \rangle _{\rho _0} \Bigr |\\  &\le \sum _{Z \subset \varLambda } \Bigl |\Bigl \langle \bigl [ \mathcal {L}_{H_0} \circ \mathcal {I}_{H_0,g}\llbracket \varPhi _A(Z) \rrbracket - \varPhi _A(Z), B \bigr ] \Bigr \rangle _{\rho _0} \Bigr |. \end{aligned} \end{aligned}$$

For the ‘small’ $Z \subset \varLambda$ satisfying ${{\,\textrm{diam}\,}}(Z) \le d( Z, \varLambda {\setminus } {\varLambda ^{\textrm{gap}}})^\beta$, we bound

103 $$\begin{aligned} \begin{aligned} \hspace{2em}&\hspace{-2em} \Bigl |\Bigl \langle \bigl [ \mathcal {L}_{H_0} \circ \mathcal {I}_{H_0,g}\llbracket \varPhi _A(Z) \rrbracket - \varPhi _A(Z), B \bigr ] \Bigr \rangle _{\rho _0} \Bigr |\\  &\le C_\textrm{gap}\, \Vert \varPhi _A(Z) \Vert \, \Vert B \Vert \, \bigl ( 1+{{\,\textrm{diam}\,}}(Y) \bigr )^\ell \, \chi _{b,p}\bigl ( d( Z, \varLambda \setminus {\varLambda ^{\textrm{gap}}}) \bigr ) \end{aligned} \end{aligned}$$

by means of Assumption $(\textrm{LDG}_{\textrm{weak}})$. Additionally, we need the following alternative estimate on (103) (recall (26)):

104 $$\begin{aligned} \hspace{2em}&\hspace{-2em} \Bigl |\Bigl \langle \bigl [ \mathcal {L}_{H_0} \circ \mathcal {I}_{H_0,g}\llbracket \varPhi _A(Z) \rrbracket - \varPhi _A(Z), B \bigr ] \Bigr \rangle _{\rho _0} \Bigr |\nonumber \\  &\le \int _ \mathbb {R} \textrm{d}t \, w_g(t) \, \bigl \Vert \bigl [ \textrm{e}^{\textrm{i}t H_0} \, \varPhi _A(Z) \, \textrm{e}^{-\textrm{i}t H_0}, B \bigr ] \bigr \Vert \nonumber \\  &\le C \, \Vert \varPhi _A(Z) \Vert \, \Vert B \Vert \, \bigl ( 1+{{\,\textrm{diam}\,}}(Z) \bigr )^d \, \biggl ( \chi _{b/2,p}\bigl ( d( Z,Y ) \bigr ) \int _I \textrm{d}t\, w_g(t) + \int _{ \mathbb {R} \setminus I} \textrm{d}t\, w_g(t) \biggr ) \nonumber \\  &\le C \, \Vert \varPhi _A(Z) \Vert \, \Vert B \Vert \, \bigl ( 1+{{\,\textrm{diam}\,}}(Z) \bigr )^d \, \chi _{b/2,p}\bigl ( d( Z,Y ) \bigr ) \end{aligned}$$

where we denoted $I:= \bigl \{ \, t \in \mathbb {R} \,\big \vert \, v|t |\le d( Z,Y )^p/3 \, \bigr \}$. Here, v is the Lieb-Robinson velocity from Lemma A.3, which we employed in the second step with b→3b/4. In the final step, we used the stretched exponential decay of $w_g$ from Lemma A.9. Note that (103) and (104) track two different relevant distances, namely those of Z to $\varLambda {\setminus } {\varLambda ^{\textrm{gap}}}$ and Y, respectively.

In fact, a weighted geometric mean of (103) and (104), that combines these two effects, can now be summed up as (neglecting the factor $C\,\Vert B \Vert \,\bigl ( 1+{{\,\textrm{diam}\,}}(Y) \bigr )^{\ell }$, which will be put back in (106))

105

For the first bound, we used logarithmic superadditivity of $\chi _{b,p}$ together with elementary monotonicity properties from Lemma A.1 (a) and estimated $\bigl ( 1+{{\,\textrm{diam}\,}}(Z) \bigr )^{d}$ by $1/\chi _{\varepsilon ,p}\bigl ( {{\,\textrm{diam}\,}}( Z ) \bigr )$. For the second bound, we used the definition of $\Vert \varPhi _A \Vert _{b,p; \varOmega }$ from (18) and the fact that, for z∈Z, we have ${{\,\textrm{diam}\,}}(Z) + d( z, \varOmega ) + d( Z, \varLambda {\setminus } {\varLambda ^{\textrm{gap}}}) \ge d( \varOmega , \varLambda {\setminus } {\varLambda ^{\textrm{gap}}})$. In the final step, we employed summability of $\chi _{\varepsilon /2,p}\bigl ( d( z,Y ) \bigr )$.

Therefore, combining (102) with (105), the contribution of those $Z \subset \varLambda$, for which ${{\,\textrm{diam}\,}}(Z) \le d( Z, \varLambda {\setminus } {\varLambda ^{\textrm{gap}}})^\beta$, to (102) is bounded by

106 $$\begin{aligned} C \, \bigl ( 1+{{\,\textrm{diam}\,}}(Y) \bigr )^{d+ \ell } \, \Vert B \Vert \, \Vert \varPhi _A \Vert _{b,p; \varOmega } \, \chi _{b-\varepsilon ,p}\bigl ( d( \varOmega , \varLambda \setminus {\varLambda ^{\textrm{gap}}}) \bigr ) . \end{aligned}$$

For the ‘large’ $Z \subset \varLambda$ that satisfy ${{\,\textrm{diam}\,}}(Z) > d( Z, \varLambda {\setminus } {\varLambda ^{\textrm{gap}}})^\beta$, we simply use the estimate from (104), which we can sum up as

107

analogously to (105). In the second step we used that ${{\,\textrm{diam}\,}}(Z) > d( Z, \varLambda {\setminus } {\varLambda ^{\textrm{gap}}})^\beta$ and elementary monotonicity properties of $\chi _{b,p}$ in b,p.

Therefore, by means of (107), also the large Z’s contribute only in a way that is controlled in terms of (106) (but with worse constants b/2 and $p \min \{\beta , 1 \}$). This concludes the proof of Lemma 6.1.

□

## Footnotes

1 This term describes adiabatic theorems for time-dependent Hamiltonians of the form $H_\varepsilon (t) = H_0(t) + \varepsilon V(t)$, where $H_0(t)$ is assumed to have a spectral gap. Now “super-adiabatic” means that for ε=0, there exists a state $\rho _0^\eta (t)$ close to the instantaneous ground state $\rho _0(t)$ of $H_0(t)$, such that the time-evolution generated by $\eta ^{-1}H_0(\,\cdot \,)$ intertwines $\rho _0^\eta (t_0)$ and $\rho _0^\eta (t)$ to any order in η. The term “generalized” means that, even for a gap-closing perturbation V, there exist super-adiabatic non-equilibrium almost-stationary states (NEASSs) $\varPi ^{\varepsilon , \eta }(t)$, which are intertwined by the time evolution generated by $\eta ^{-1} H_\varepsilon (\,\cdot \,)$ to any order in ε and η. We refer to [26, 27, 29, 40, 49] for details; see also Sects. 4.2 and 6.2.

2 For ease of notation and since there will be no perturbation εV in the current Sect. 1.2, we will drop the subscript 0 here.

3 We will follow the convention that *(orthogonal) projections* will be denoted by P (i.e. satisfying $P^2 = P$ and $P^* = P$), while *states* are denoted by ρ (i.e. satisfying $\rho = \rho ^*$ and $0 \le \rho \le 1$ with ${{\,\textrm{tr}\,}}\rho = 1$). Clearly, if P is an orthogonal projection, then $\rho := P/\dim {{\,\textrm{rank}\,}}P$ is a state.

4 Throughout this paper, $\mathcal {A}_X$ denotes the algebra of observables with support in X.

5 For ${\varLambda ^{\textrm{gap}}}= \varLambda$, this condition for all observables is actually equivalent to the usual spectral gap condition.

6 The bound in [26, eq. (4.1)] essentially means that the unperturbed system on $\varLambda \equiv \varLambda _k$ is gapped within ${\varLambda ^{\textrm{gap}}}\equiv \varLambda _{\lfloor k(1-o(1)) \rfloor }$, where $\varLambda _l$ denotes the box of side length 2l+1 in ${ \mathbb {Z} ^d}$ centered at zero.

7 We use the convention that $\widehat{f}(p):= (2 \pi )^{-1/2} \int _ \mathbb {R} \textrm{d}x \, \textrm{e}^{- \textrm{i}p x} f(x)$ for the Fourier transform.

8 This fact is used for proving $(\textrm{LDG}_{\textrm{main}})$ (which is done in Sect. 5) for one of the examples discussed in Sect. 3.5 below.

9 More precisely, this also requires that an interaction $\varPhi = \varPhi _H$, associated to the SLT Hamiltonian H, *has a thermodynamic limit* in a suitable sense (see [27, Definition 2.1], [26, Definition 3.1], and [29, Definitions 2 and 5]). In this case, the linear functional $A \mapsto {{\,\textrm{tr}\,}}( PA )$ converges in $\textrm{weak}^*$ sense to a *state* on the $C^*$-algebra of quasi-local observables on Γ. A *gap in the bulk* then means, that the naturally associated GNS Hamiltonian of the infinite system has a spectral gap above zero.

10 Following the *resummation procedure* in [27, Appendix E], one could even construct a single (i.e. n independent) state $\varPi ^{\varepsilon }$, which, for every fixed n, has the same properties as $\varPi _n^{\varepsilon }$. We will, however, refrain from doing so for brevity of the presentation.

11 This is simply a common eigenbasis of H and $H\big \vert _{{\varLambda ^{\textrm{gap}}}} \otimes \textbf{1}_{\mathcal {A}_{\varLambda {\setminus } {\varLambda ^{\textrm{gap}}}}}$, which commute.
